# In Vitro Biological Activity of Natural Products from the Endophytic Fungus *Paraboeremia selaginellae* against *Toxoplasma gondii*

**DOI:** 10.3390/antibiotics11091176

**Published:** 2022-08-31

**Authors:** Flaminia Mazzone, Viktor E. Simons, Lasse van Geelen, Marian Frank, Attila Mándi, Tibor Kurtán, Klaus Pfeffer, Rainer Kalscheuer

**Affiliations:** 1 Institute of Medical Microbiology and Hospital Hygiene, Heinrich Heine University, 40225 Duesseldorf, Germany; 2 Institute of Pharmaceutical Biology and Biotechnology, Heinrich Heine University, 40225 Duesseldorf, Germany; 3 Department of Organic Chemistry, University of Debrecen, 4002 Debrecen, Hungary

**Keywords:** *Toxoplasma gondii*, *Paraboeremia selaginellae*, endophytic fungi, natural products, bioactivity, biphenyl ether, bioxanthracene, phomalactone

## Abstract

*Toxoplasma gondii* is an apicomplexan pathogen able to infect a wide range of warm-blooded animals, including humans, leading to toxoplasmosis. Current treatments for toxoplasmosis are associated with severe side-effects and a lack efficacy to eradicate chronic infection. Thus, there is an urgent need for developing novel, highly efficient agents against toxoplasmosis with low toxicity. For decades, natural products have been a useful source of novel bioactive compounds for the treatment of infectious pathogens. In the present study, we isolated eight natural products from the crude extract of the endophytic fungus *Paraboeremia selaginellae* obtained from the leaves of the plant *Philodendron monstera*. The natural products were tested for inhibiting *Toxoplasma gondii* proliferation, and their cytotoxicity was evaluated in different human cell lines. Six natural products showed antitoxoplasma activity with low or no cytotoxicity in human cell lines. Together, these findings indicate that biphenyl ethers, bioxanthracenes, and 5*S*,6*S*-phomalactone from *P. selaginellae* are potential candidates for novel anti-toxoplasma drugs.

## 1. Introduction

*Toxoplasma gondii* is an obligate intracellular protozoan parasite member of the phylum Apicomplexa, which includes known human pathogens such as *Plasmodium* sp., *Eimeria* sp., *Neospora*, *Babesia*, *Theileria,* and *Cryptosporidium* spp., with which it shares significant biological similarities [1]. Beyond these organisms, the study of *T. gondii* has experimental advantages since its basic biology and the methodology for the genetic manipulation and quantification of its different stages are well established. Thus, *T. gondii* is considered a major model for the study of apicomplexan biology and for anti-apicomplexan drug target validation [2]. *T. gondii* infections are among the most common human zoonoses, leading to toxoplasmosis disease [3]. *T. gondii* is considered one of the world’s most successful parasites due its ability to infect a wide range of warm-blooded vertebrate intermediate hosts [4]. *T. gondii* is estimated to chronically infect one-third of the world’s human population and is acquired mainly through two ways: by ingesting oocysts shed from feline hosts (the definitive hosts) in contaminated food or water and by the consumption of raw or undercooked meat containing viable tissue cysts [5]. Waterborne and food-borne outbreaks of toxoplasmosis have been reported from countries with diverse cultural, social, and ethnic backgrounds [6]. In immunocompetent individuals, infection with *T. gondii* is usually asymptomatic or has a subclinical course with mild symptoms. In contrast, immunocompromised (i.e., acquired immune deficiency syndrome (AIDS), organ transplant or cancer) patients can develop the disease, leading to life-threatening cerebral and ocular toxoplasmosis due to a reactivation of the latent infection. Additionally, primary infection in pregnant women may result in fetal death, spontaneous abortion, and birth defects [7,8,9]. Although many gaps have been filled in the epidemiological, diagnostic, and biological fields to understand of the interaction of the parasite with the host, little progress has been made in drug discovery for the treatment of toxoplasmosis.

Current treatments of acute toxoplasmosis are largely limited to anti-folate therapy. Pyrimethamine and sulfadiazine, the current gold-standards for the treatment of toxoplasmosis, can suppress the parasite growth in the active stage of the infection by targeting the tachyzoite stage, but they have no effect in the bradyzoites stage. Additionally, they have been found to have high rates of toxic side effects, leading to discontinuation of therapy. Thus, there is an urgent need to identify novel potent candidates that would be well-tolerated to eradicate latency as well as to treat the acute infection [10,11]. Natural products profoundly impact the history of drug discovery, especially in the research of novel anti-cancer, anti-bacterial, and anti-parasitic treatments. Nature continues to provide diverse and unique chemical sources of bioactive lead compounds that inspire novel drug discoveries [12]. The antiparasitic bioactivity of natural products from various sources, especially plant-derived secondary metabolites, has been deeply investigated in in vitro and in vivo studies [13]. Many fungal metabolites have also been reported to exhibit antimicrobial properties against parasitic pathogens. However, most of these studies focused on bioactivity against *Plasmodium falciparum*, whereas there is a scarcity of investigations to explore the potential of fungi as a source of novel anti-toxoplasma agents [14].

In this study, we extracted and purified eight natural products from the crude extract of *Paraboeremia selaginellae*, an endophytic fungus isolated from the ornamental plant *Philodendron monstera*. Isolated compounds were structurally characterized and evaluated for their anti-toxoplasma activities. Biphenyl ethers, bioxanthracenes, and phomalactone showed substantial activity against *T. gondii* proliferation. Therefore, we suggest these compounds as promising candidates for novel anti-parasitic therapies.

## 2. Results

### 2.1. Isolation of Compounds from Paraboeremia selaginella

**Figure 1:**
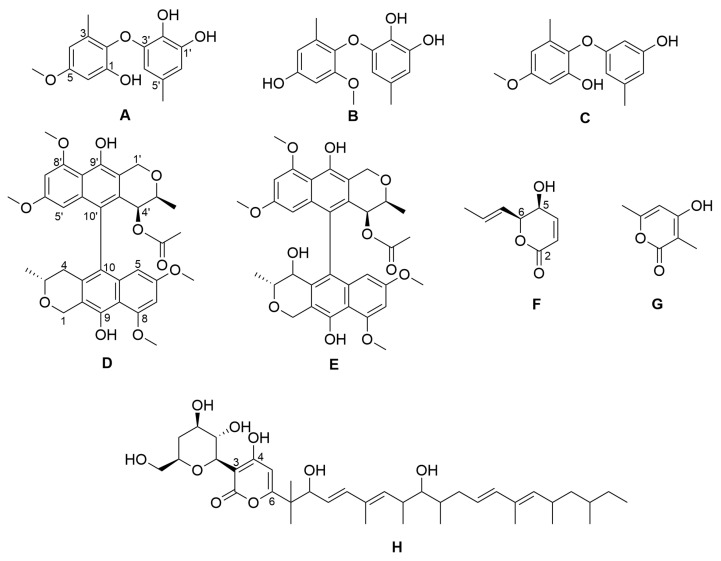
Chemical structures of the isolated compounds. NK-A 17e233 (**A**); 1,2-benzenediol, 3-(4-hydroxy-2-methoxy-6-methylphenoxy)-5-methyl-(ACI) (**B**); cyperin (**C**); ES-242-1 (**D**); ES-242-3 (**E**); 5*S*,6*S*-phomalactone (**F**); methyltriaceticlactone (**G**); S 39163/F-1 (**H**).

We isolated an endophytic fungus from fresh surface-sterilized leaves of the ornamental plant *Philodendron monstera*. The isolated strain was identified as *Paraboeremia selaginella* by the internal transcribed spacer (ITS) sequence with 99.56% identity in comparison with the ITS database of the National Center for Biotechnology Information. From the crude extract of a culture of *Paraboeremia selaginella* grown on solid rice medium, eight compounds were isolated by chromatographic methods and structurally elucidated by complementary spectroscopic analyses (Figure 1). All eight compounds have previously been reported from other sources but are reported here for the first time as natural products occurring in the genus *Paraboeremia*.

**Figure 2:**
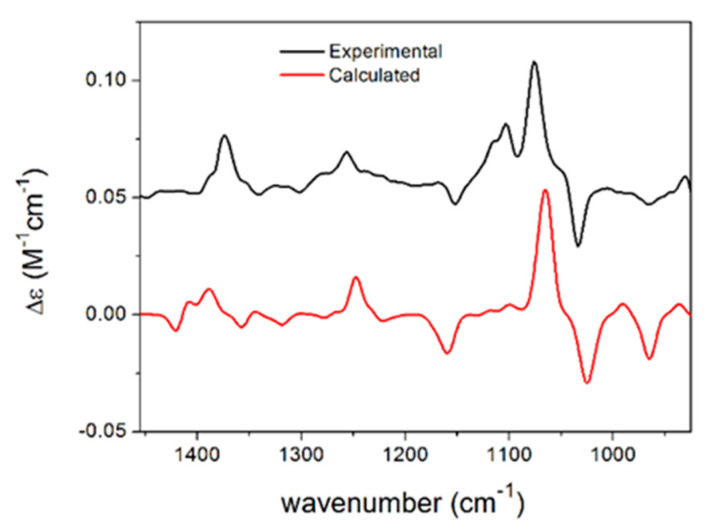
Comparison of the experimental VCD spectrum of **F** measured in CDCl_3_ and the calculated VCD spectrum of *cis*-(5*S*,6*S*)-**F** computed at the B3LYP/TZVP PCM/CHCl_3_ level for the eight lowest-energy conformers gained from the DFT optimization performed at the same level.

Different stereoisomers of phomalactone (**F**) were isolated and reported previously from various sources [15,16,17,18], and some papers did not specify the absolute configuration [19,20], while others assigned the (5*R*,6*R*) absolute configuration to the large positive specific rotation [21], which was opposite to previous studies [15,16,17,18,22]. In order to determine the absolute configuration of phomalactone independently and unambiguously, we performed TDDFT-ECD, TDDFT-OR, and DFT-VCD studies, which consistently confirmed the (+)-*cis*-(5*S*,6*S*) absolute configuration (see Supplementary Materials) [23,24]. Comparisons of the experimental and computed VCD spectra of *cis*-(5*S*,6*S*) are shown in Figure 2, which produced good agreement. Other computational results are shown in the Supplementary Materials.

### 2.2. Anti-T. gondii Activity

**Figure 3:**
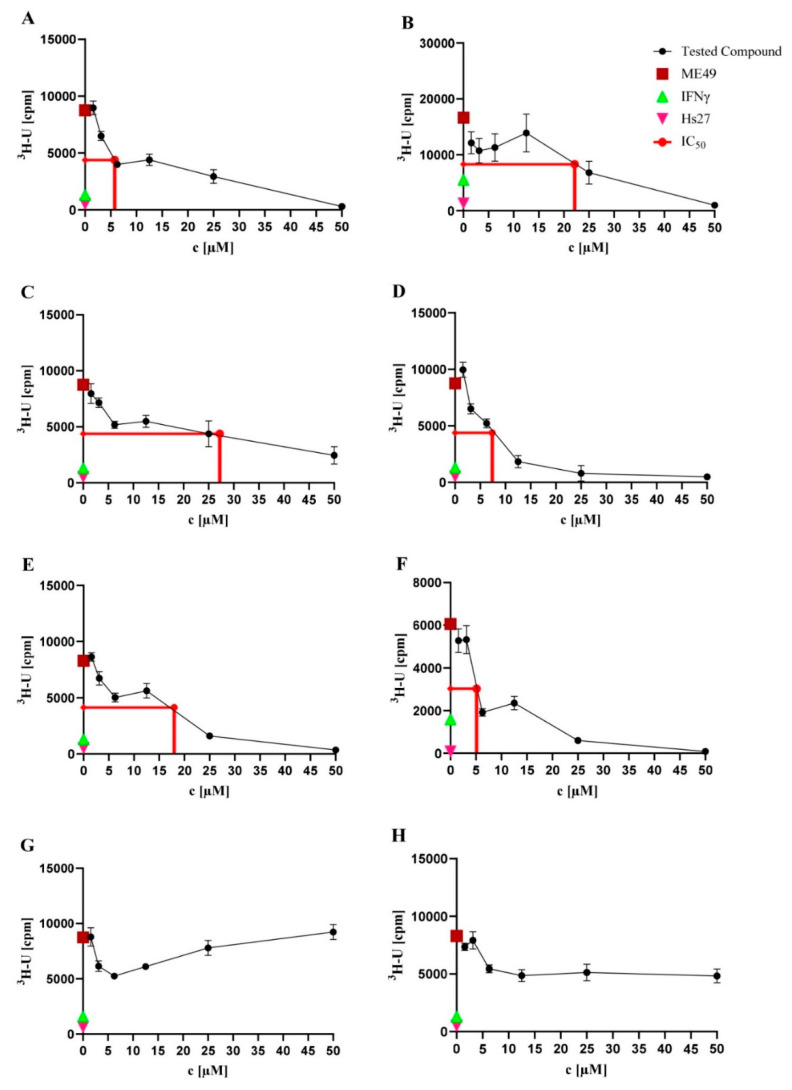
Toxoplasma proliferation assays. Toxoplasma proliferation assays were performed to investigate the activity of the natural products against *T. gondii* strain ME49. Hs27 cells were cultured in a monolayer in 96-well plates and infected with *T. gondii* (3 × 10^4^). Cultures were treated with the natural products at the concentration range of 0.56–50.00 µM for 48 h at 37 °C. Afterwards, the cultures were labelled with ^3^H-U (5 mCi, diluted 1:30) for 28–30 h at 37 °C. Based on the incorporation of ^3^H-U into the parasite nucleic acid, the parasite growth was quantified. As controls, uninfected Hs27 cells without treatment (pink triangles) and IFNγ pre-stimulated infected Hs27 cells (green triangles) were used, while untreated *T. gondii*-infected Hs27 cells (red squares) served as a negative control. Values shown in (**A**–**H**) represent the means of three independent experiments each done in duplicate (*n* = 6) ± SEM. The mean of the IC_50_ values (red line) of each compound is shown. Activity of NK-A 17e233 (**A**); 1,2-benzenediol, 3-(4-hydroxy-2-methoxy-6-methylphenoxy)-5-methyl-(ACI) (**B**); cyperin (**C**); ES-242-1 (**D**); ES-242-3 (**E**); 5*S*,6*S*-phomalactone (**F**); methyltriaceticlactone (**G**); S 39163/F-1 (**H**).

The eight natural products isolated from *P. selaginellae* were tested for anti-*T. gondii* activity. Interestingly, **A**–**F** showed activity against *T. gondii* growth, with IC_50_ values of 5.75, 22.16, 27.22, 7.38, 17.99, and 5.13 µM, respectively (Table 1 and Figure 3). Therefore, we further explored the in vitro cytotoxicity of the natural compounds in different human cell lines.

### 2.3. Cytotoxicity Assays

**Figure 4:**
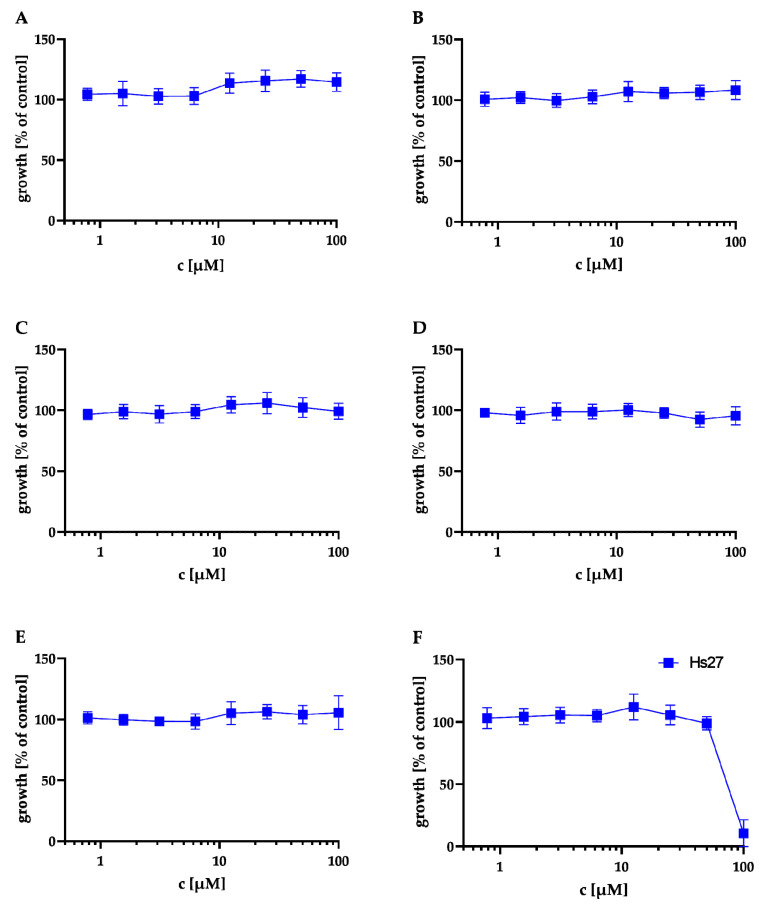
Effect of the natural products on the metabolic activity of Hs27 cells via MTT assay. Hs27 cells were plated in 96-well plates and grown to confluence prior to incubation at 37 °C for 24 h with the natural products in the concentration range of 0.56–100.00 μM. The cultures were incubated with 10 μL of the 12 mM MTT stock solution for approximately 4 h. Afterwards, 100 μL of SDS dissolved in HCl was added to each well and incubated again for 4 h at 37 °C. Finally, the absorbance was measured at 570 nm by spectrophotometry. Values shown in (**A**–**F**) represent the means of three independent experiments each done in duplicate (*n* = 6) ± SEM. Cytotoxicity in Hs27 cells of NK-A 17e233 (**A**); 1,2-benzenediol, 3-(4-hydroxy-2-methoxy-6-methylphenoxy)-5-methyl-(ACI) (**B**); cyperin (**C**); ES-242-1 (**D**); ES-242-3 (**E**); 5*S*,6*S*-phomalactone (**F**).

First, we evaluated the cytotoxicity of compounds **A** to **F** in an MTT assay against Hs27 human fibroblasts (same cell type used for the *T. gondii* proliferation assay). The results of the MTT assay are shown in Figure 4 and Table 2. **A**–**E** showed no cytotoxicity at 100 μM against Hs27 cells. Only **F** showed moderate cytotoxicity with a cytotoxic concentration CC_50_ = 81 ± 2.16 µM.

**Figure 5:**
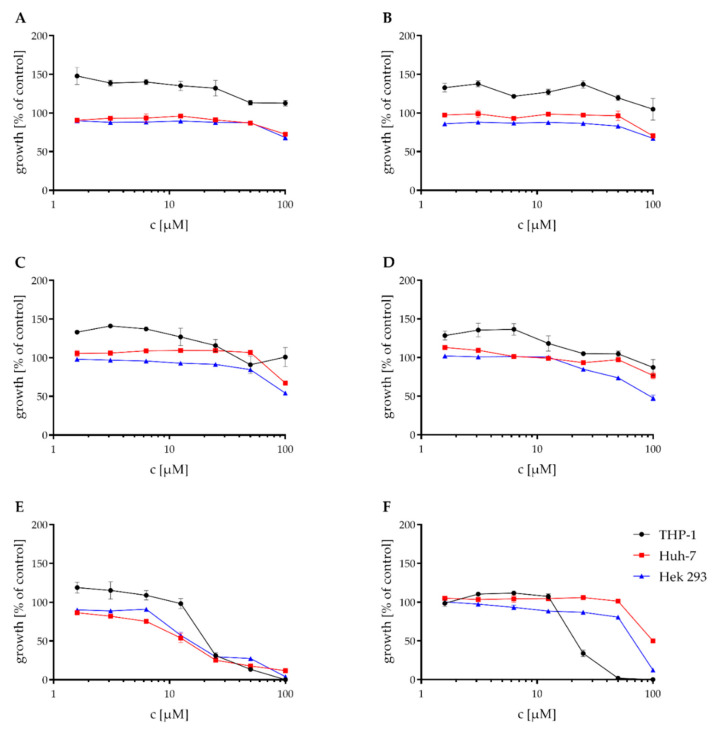
Effect of the natural products on the viability of THP-1, Huh-7, and HEK-293 cells. Cytotoxic effect of NK-A17e233 (**A**); 1,2-benzenediol,3-(4-hydroxy-2-methoxy-6-methylphenoxy)-5-methyl-(ACI) (**B**); cyperin (**C**); ES-242-1 (**D**); ES-242-3 (**E**); 5*S*,6*S*-phomalactone (**F**) against the human cell lines THP-1, Huh-7, and HEK-293 as determined by resazurin assay. 100% growth control DMSO, 0% growth control cycloheximide. Values represent the means of triplicates ± SEM.

These compounds also were tested against the THP-1, Huh-7, and Hek 293 cell lines in a Resazurin assay. The mean IC_50_ values of the Resazurin assay are shown in Table 3. While compounds **A**–**C** showed no cytotoxic effect in concentrations < 100 μM against any of the tested cell lines, **D** had only a weak cytotoxic activity against the Hek 293 cell line with an IC_50_ of 93.8 μM. **E** showed moderate cytotoxic activity against all of the three tested cell lines and thus was the most cytotoxic of the tested compounds. **F** showed no or weak cytotoxic effects against the Huh-7 and Hek-293 cell lines. The cytotoxic effect against the THP-1 cell line was higher, with an IC_50_ of 24.3 μM. The graphs for the Resazurin assay are shown in Figure 5.

### 2.4. Determination of Anti-Bacterial Activity

In our ongoing research for antibacterial and particularly antitubercular compounds, **A** to **H** were also tested in a minimal inhibitory concentration assay against *S. aureus* ATCC 700699, *P. aeruginosa* ATCC 87110, and *M. tuberculosis* H37Rv. Compounds **A** to **H** had no inhibitory effect on *S. aureus* ATCC 700699 and *P. aeruginosa* ATCC 87110, except for compound **F**, which showed a weak inhibitory effect on *P. aeruginosa* ATCC 87110 with an MIC_90_ of 100 µM. Compounds **A**, **B**, **C**, **F**, **G**, and **H** had no inhibitory effect on the growth of *M. tuberculosis*, and compounds **D** and **E** showed a weak inhibitory effect with an MIC_90_ of 50 and 100 μM, respectively. The results are shown in Table 4, highlighting that compounds **A** to **F** had a specific anti-toxoplasma effect and were devoid of broad, unspecific antimicrobial activity.

## 3. Discussion

Natural products have played an important role in the history of drug discovery for infectious disease. In the quest for new anti-*T. gondii* drugs, natural products have been proven to exhibit high potential for the discovery and development of new lead compounds with strong anti-*T. gondii* activity [25,26]. In this study, we isolated eight natural products from the crude extract of the endophytic fungus *P. selaginellae*. A previous report on the inhibitory activity of one of these compounds (phomalactone, **F**) against the apicomplexan parasite *Plasmodium falciparum* with an IC_50_ of 84.32 µM [27] prompted us to test the natural products for anti-*T. gondii* activity. Interestingly, six compounds showed activity against *T. gondii* proliferation with no or low cytotoxicity in different human cell lines and no or low antibacterial activity against a gram-positive, a gram-negative, and a mycobacterial representative, revealing reasonable anti-*T. gondii* specificity and promising therapeutic windows. These results establish diphenyl ethers, bioxanthracenes, and lactones from *P. selaginellae* as potential candidates for further preclinical development of novel anti-toxoplasma therapeutics.

Some of the isolated compounds share similar structural elements, which give insights into a structure–activity relationship of the natural products against the tested *T. gondii* strain ME49. Compounds **A**, **B**, and **C** are biphenyl ether derivatives that differ either in the position of the methoxy group or in the number of substituted hydroxyl groups. The most potent of these compounds is **A** (IC_50_ = 5.75 µM), followed by **B** (IC_50_ = 19.35 µM) and **C** (IC_50_ = 27.22 µM). While **A** only differs from **B** by a switch in the position of the methoxylated hydroxyl group from position 2 to 4, it differs from **C** only by an additional hydroxyl group in position 2′, which it shares with **B**. Because of the higher potency of **A** in the toxoplasma proliferation assay compared to **B** and **C**, the position of the methoxy group in 4 and the amount and position of hydroxyl groups in 2′ and 3′ both are likely to have an influence on the antitoxoplasma activity of these derivatives. This suggestion, nevertheless, needs further experimental evidence. Furthermore, diphenyl ethers **A**, **B**, and **C** are structurally related to triclosan, a well-known broad spectrum antifungal and antibacterial agent targeting lipid synthesis [28]. It has been shown that triclosan also inhibits the growth of apicomplexans by inhibition of the enoyl reductase ENR (FabI) enzyme, the second reductive step in the type II fatty acid biosynthesis pathway. Nevertheless, due the poor solubility of triclosan, there is considerable interest in finding novel potent triclosan analogs with improved properties such as solubility, activity, and toxicity [29,30]. The mechanism of action of **A**, **B**, and **C** may be similar to that of triclosan, but further studies are necessary to explore and confirm their mode of action and cellular target. Furthermore, in vitro and in vivo pharmacokinetic characterization is needed to reveal whether any of the compounds reported here has superior properties compared to triclosan.

Compounds **D** and **E** represent bioxanthracenes belonging to the ES-242 class and share the same structure, differing only in position 4′ by the hydroxyl group that is present only in **E**. The IC_50_ values in the toxoplasma proliferation assay were 7.38 µM and 17.99 µM for **D** and **E**, respectively, suggesting a reduction in the antitoxoplasma activity if position 4′ is substituted by a hydroxyl group. The bioxanthracenes **D** and **E** were previously isolated from *Verticillium* spp. and are well-known to act as *N*-methyl-*D*-aspartate receptor antagonists [31]. Both compounds were also found to be active against the apicomplexan parasite *P. falciparum* with IC_50_ values of 8.44 and 13.22 µM, respectively [32]. Interestingly, the activities of **D** and **E** against *T. gondii* in this study were comparable to their reported activity in *P. falciparum* with IC_50_ values of 7.38 µM and 17.99 µM (see Table 1). Nevertheless, the mechanism of action of **D** and **E** on apicomplexans is still unknown and is probably independent from their activity as NMDA receptor antagonists [32].

Compounds **F** and **G** are small δ-lactonic molecules; 5*S*,6*S*-phomalactone (**F**) differs from methyltriaceticlactone (**G**) in the length of the sidechain in position 6, the hydroxyl group in position 5, and in the absence of the methyl group that is present in methyltriaceticlactone in position 2. Interestingly, antitoxoplasmal activity was observed for **F**, but not for **G**, suggesting that one or more of these structural differences and not only the presence of the δ-lactonic base structure plays a crucial role in the bioactivity against *T. gondii*. Phomalactone (**F**) is a frequent fungal metabolite and was first isolated from the plant-pathogenic fungus *Nigrospora* sp. [16]. It has a wide range of activities such as antifungal, immunomodulating, insecticide, nematocidal, and phytotoxic activity [15,19,33,34,35]. In addition, it has been found to be active against the apicomplexan parasite *P. falciparum,* with an IC_50_ of 84.32 µM [27]. In the present study, we tested **F** for inhibition of *T. gondii* proliferation and, interestingly, it showed a more potent activity with an IC_50_ of 5.13 µM (see Table 1). No target or mode of action has been suggested for phomalactone in *P. falciparum*, and the target of this compound in *T. gondii* also remains elusive and has to be determined in the future. Importantly, the newly identified natural products with inhibitory activity against *T. gondii* showed very little in vitro toxicity and should be evaluated in in vivo infection model systems in the future. In general, this study highlights the potential of endophytic fungi as a promising source for novel antitoxoplasma compounds.

## 4. Materials and Methods

### 4.1. General Experimental Procedures

Optical rotations were measured on a Jasco P-2000 polarimeter (Jasco, Pfungstadt, Germany). UV-spectra were obtained by the use of a Dionex P580 system in combination with a diode array detector (UVD340S) and an Eurosphere 10 C18 column (125 mm × 4 mm). ECD spectra were measured on a JASCO J-810 spectropolarimeter. VCD spectra were recorded on a BioTools Chiral-IR-2X at a resolution of 4 cm^−1^ under ambient temperature for 18 × 3000 scans. Samples were dissolved in CDCl_3_, and the solution was placed in a 100 µm BaF_2_ cell. 1D and 2D NMR spectra were recorded on a Bruker Avance III (^1^H, 600 MHz; ^13^C 150 MHz) spectrometer. Mass spectra were measured on a Finnigan LCQ Deca (Thermo Quest, Egelsbach, Germany) mass spectrometer and for HRESIMS, on a UHR-QTOF maXis 4G (Bruker Daltonics, Bremen, Germany) mass spectrometer. Semipreperative HPLC was performed on a Lachrom-Merck Hitachi system (pump L7100, UV-detector L7400, Eurospher 100 C18 column 300 mm × 8 mm, Knauer, Everswinkel, Germany). VLC and non-vacuum-column chromatography were accomplished using Macherey Nagel silica gel 60M (0.04–0.063 mm). Precoated TLC silica gel 60 F254 plates (Merck, Darmstadt, Germany) were used for tracking separation using detection under UV light at 254 and 365 nm wavelengths or spraying anisaldehyde–sulfuric acid reagent. Sephadex LH20 (GE Healthcare Bio.Sciences AB, Uppsala, Sweden) was used as a stationary phase for column chromatography. The measurement of optical rotations was accomplished by using spectral grade solvents.

### 4.2. Fungal Material

The fungus was obtained from the leaves of the plant *Philodendron monstera* as an endophyte. A single leaf was surface sterilized by soaking it with 70% ethanol for 30 s and letting it dry under sterile conditions. With a heat-sterilized scalpel, the leaf was cut into pieces and put onto a YPD agar plate, which was enriched with 100 mg/L chloramphenicol to suppress bacterial growth. After seven days of incubation at room temperature, distinct fungal growth was observed on the plate. A 1 cm^2^ piece of the fungus was cut out of the agar medium using a heat-sterilized scalpel under sterile conditions and was transferred onto a new sterile YPD agar plate to isolate a pure organism. The isolated strain was identified as *Paraboeremia selaginella* by the internal transcribed spacer (ITS) sequence with 99.56% identity in comparison with the ITS database of the National Center for Biotechnology Information (GenBank Accession ON231784).

### 4.3. Fermentation and Extraction

The fungus was fermented on solid rice medium. Ten Erlenmeyer flasks were used; 100 g of rice and 100 mL of demineralized water were added to each flask and autoclaved at 121 °C for 15 min. Under sterile conditions, 1 cm^2^ of fungal material was cut out of an agar plate using a sterile scalpel and transferred onto the autoclaved rice medium. The fungus was grown for 4 weeks under static conditions at room temperature. Each flask was soaked with 250 mL of ethylacetate for at least 12 h. The rice medium was then cut into small pieces and shaken for 8 h at 150 rpm. The liquid crude extract was filtrated into round flasks and evaporated using a rotary evaporator to yield 14.66 g of dry crude extract.

### 4.4. Isolation

The crude extract (14.66 g) obtained from the fermentation was separated using vacuum liquid chromatography with silica gel as a stationary phase. A step gradient from 100% hexane to 100% ethylacetate followed by a step gradient from 100% dichloromethane to 100% methanol gave 18 fractions (V1–V18). Two fractions (V4 and V6) were chosen based on initial bioactivity observed against *Candida albicans*. However, this bioactivity was lost during the purification process. Fraction V4 (200.7 mg) was further separated using a Sephadex LH20 column with MeOH as eluent to give five subfractions (V4-S1–S5). Fraction V4S3 (47.3 mg) was subjected to semipreparative HPLC using a MeOH-H_2_O step gradient from 50% to 80% MeOH followed by a washing step with 100% MeOH to yield **A** (20.7 mg), **B** (2.8 mg), and **C** (7.7 mg). Fraction V6 (1010 mg) was purified using a Sephadex LH20 column with CH_2_Cl_2_ and MeOH (50/50) as eluent to yield six subfractions (V6-S1–S6). Subfraction S2 (72.0 mg) was purified using a silica column with 40% hexane and 60% ethylacetate to elute **D** (25.0 mg) and **E** (8.6 mg) as pure compounds. Subfraction V6-S4 (516 mg) was further purified by using a Sephadex LH20 column with MeOH as eluent to yield five subfractions (V6S4-S1–S5). Subfraction V6S4S2 (496 mg) was subjected to a silica column with a mixture of CH_2_Cl_2_ and MeOH (95/5) as eluent to give four subfractions (V6S4S2-K1–K4). Silica subfraction V6S4S2K2 (47 mg) was then purified by semipreparative HPLC using a MeOH-H_2_O step gradient from 10% to 30% MeOH followed by a washing step with 100% MeOH to yield **F** (22.2 mg) and **G** (4.5 mg). Fraction V12 (744.1 mg) was separated using a Sephadex LH20 column with 50% MeOH and 50% CH_2_Cl_2_ to yield six subfractions (V12-S1–S6). Subfraction S2 (180.3 mg) was then further separated using a silica column with 10% MeOH and 90% CH_2_Cl_2_ as eluent to give seven subfractions (V12S2-K1–K7). Silica subfraction K7 (56.1 mg) was subjected to semipreparative HPLC using a step gradient from 70% to 100% MeOH to yield **H** (5.0 mg).

**NK-A 17e233 (A):** Brown oil; UV (MeOH) λ_max_ 220.0, 234.3, 279.7 nm; ^1^H NMR (DMSO-d6) see Supplementary Materials Figure S1; HRESIMS *m/z* 277.1075 [M + H]^+^ (calcd. for C_15_H_17_O_5_ 277.1071 *m/z*).

**3-(4-Hydroxy-2-methoxy-6-methylphenoxy)-5-methylbenzene-1,2-diol (B):** Brown oil; UV (MeOH) λ_max_ 211.7, 286.3 nm; ^1^H NMR (CDCl_3_), see Supplementary Materials Figure S6; HRESIMS *m/z* 277.1065 [M + H]^+^ (calcd. for C_15_H_17_O_5_ 277.1071 *m/z*).

**Cyperin (C):** Brown oil; UV (MeOH) λ_max_ 212.1, 279.8 nm; ^1^H NMR (CDCl_3_), see Supplementary Materials Figure S10; HRESIMS *m/z* 261.1126 [M + H]^+^ (calcd. for C_15_H_17_O_4_ 261.1121 *m/z*).

**ES-242-1 (D):** Brown amorphous powder; [*α*]^24^_*D*_ +*18* (c 1.0, MeOH); UV (MeOH) λ_max_ 239.0, 309.8, 345.8 nm; ^1^H NMR (CDCl_3_) and ^13^C NMR (CDCl_3_), see Supplementary Materials Figures S14 and S15; HRESIMS *m/z* 622.2644 [M + NH_4]_^+^ (calcd. for C_34_H_40_NO_10_ 622.2647 *m/z*).

**ES-242-3 (E):** Brown amorphous powder; [*α*]^24^_*D*_ +*66* (c 1.0, CHCl_3_); UV (MeOH) λ_max_ 239.2, 298.6, 309.4 nm; ^1^H NMR (CDCl_3_) and ^13^C NMR (CDCl_3_), see Supplementary Materials Figures S19 and S20; HRESIMS *m/z* 638.2588 [M + NH_4]_^+^ (calcd. for C_34_H_40_NO_11_ 638.2596 *m/z*).

**Phomalactone (F):** light yellowish oil; [*α*]^24^_*D*_ +*172* (c 1.0, EtOH); UV (MeOH) λ_max_ 216.0 nm; For the details of VCD, ECD, and OR calculations, see Supplementary Materials*;* ^1^H NMR (CDCl_3_) and ^13^C NMR (CDCl_3_), see Supplementary Materials Figures S24 and S25; HRESIMS *m/z* 155.0702 [M + H]^+^ (calcd. for C_8_H_11_O_3_ 155.0703 *m/z*) and *m/z* 137.0597 [M − OH^−^]^+^ (calcd. for C_8_H_9_O_2_ 137.0597 *m/z*).

**Methyltriaceticlactone (G):** White amorphous powder; UV (MeOH) λ_max_ 290.5 nm; ^1^H NMR (DMSO-d6) and ^13^C NMR (DMSO-d6), see Supplementary Materials Figures S29 and S30; HRESIMS *m/z* 141.0549 [M + H]^+^ (calcd. for C_7_H_9_O_3_ 141.0546 *m/z*).

**S 39163/F-1 (H):** Brown amorphous gum; [*α*]^24^_*D*_ −*11* (c 1.0, MeOH); UV (MeOH) λ_max_ 218.2, 238.8, 291.9 nm; ^1^H NMR (CDCl_3_), see Supplementary Materials Figure S34; HRESIMS *m/z* 661.4312 [M + H]^+^ (calcd. for C_38_H_61_O_9_ 661.4310 *m/z*).

### 4.5. Preparation of Compounds for T. gondii Proliferation Assay

The purified natural products **A**–**F** and pyrimethamine [36] were dissolved in DMSO as 10 mM stocks and stored at −20 °C. The compounds were diluted in Iscove’s Modified Dulbecco’s medium (Gibco–Thermo Fisher Scientific, Braunschweig, Germany) immediately prior to use.

### 4.6. Parasites and Cell Culture for T. gondii Proliferation Assay

*T. gondii* ME49 tachyzoites (ATCC/LGC Standards GmbH, Wesel, Germany) were cultured in human foreskin fibroblast Hs27 cells (ATCC/LGC Standards GmbH, Wesel, Germany) as host cells as described previously [37]. The cells were maintained in Iscove’s modified Dulbecco’s medium (Gibco–Thermo Fisher Scientific, Braunschweig, Germany) supplemented with 10% heat-inactivated fetal bovine serum (Invitrogen, Karlsruhe, Germany) and 50 mM 2-mercaptoethanol (Gibco–Thermo Fisher Scientific, Braunschweig, Germany) and were grown in a humidified incubator at 37 °C with 5% CO_2_ in air atmosphere. For toxoplasma propagation, 25 cm^2^ cell culture flasks, containing a confluent monolayer of Hs27 cells, were infected with 5 × 10^6^ *T. gondii* tachyzoites after medium change. After three days, the supernatant of the cell culture containing parasites was harvested and transferred to a 15 mL centrifuge tube and centrifuged at 700 rpm for five minutes and resuspended in cell culture medium. The number of parasites was counted using a hemocytometer.

### 4.7. T. gondii Proliferation Assay

Microtiter plates (96-well) with a final volume of 200 μL per well were used for the assay. Hs27 fibroblast monolayers were infected with 3 × 10^4^ freshly harvested tachyzoites per well (MOI = 1) and incubated for 48 h at 37 °C, after which various concentrations of the tested compounds (0.04, 0.09, 0.19, 0.39, 0.78, 1.5, 3.12, 6.25, 12.5, 25, 50 µM) in culture medium were added to the cells. Pyrimethamine (0.007, 0.01, 0.03, 0.06, 0.125, 0.25, 0.5, 1 µM) was added under identical conditions as a positive drug control [37]. Hs27 cells were pre-stimulated for 24 h with IFNγ (300 U/mL) and infected with *T. gondii* cells without further treatment as the growth inhibition control. After 48 h, proliferating toxoplasma parasites were radioactively labelled with tritiated uracil (5 mCi, Hartmann Analytic, Braunschweig, Germany) and diluted 1:30 (10 μL per 200 μL total culture volume per well) in order to determine parasite proliferation [38]. After 28–30 h, the microtiter plates were frozen at −20 °C. To evaluate the assay, the microtiter plates were thawed at room temperature. Cells were transferred to glass-fiber filters (Printed Filtermat A 102 mm × 258 mm, PerkinElmer, Waltham, MA, USA) using a cell harvester (Basic96 Harvester, Zinsser Analytic, Skatron Instruments, Northridge, CA, USA). The filters were dried for 20 min at 130 °C in a drying cabinet and were then soaked in 10 mL of scintillation fluid (Betaplate Scint, PerkinElmer, Waltham, MA, USA) and shrink-wrapped in plastic covers (Sample Bag for Betaplate, PerkinElmer, Waltham, MA, USA). The filters were then clamped in cassettes and evaluated using a beta-counter device (Betaplate Liquid Scintillation Counter 1205, LKB-WALLAK, Melbourne, Australia) to measure the Cherenkov radiation, which refers to the amount of incorporation of tritiated uracil into the RNA of *T. gondii*. IC_50_ values, the concentration of inhibitors necessary to inhibit the growth of tachyzoites by 50%, were determined for each experiment with the use of Prism GraphPad version 9.2.0 software.

### 4.8. Cell Viability Assay against Hs27 Cells

The 3-[4,5-dimethylthiazole-2-yl]-2,5-diphenyltetrazolium bromide (MTT) test was used to assess cell viability of the isolated active compounds against Hs27 cells. The MTT assay is a colorimetric reaction based on the enzymatic reduction of MTT to MTT-formazan, which is catalyzed by mitochondrial succinate dehydrogenase [39].

In brief, Hs27 cells were seeded 96-well plates in a monolayer in Iscove’s modified Dulbecco’s medium (Gibco–Thermo Fisher Scientific, Braunschweig, Germany) and incubated at 37 °C with different concentrations of the tested natural products (1.56, 3,12, 6.25, 12,5, 25, 50, 100 µM) in the culture media. Staurosporine (0.007, 0.01, 0.03, 0.06, 1.25, 0.25, 0.5, 1 µM), a well-known cytotoxicity-inducing kinase inhibitor [40], untreated Hs27 cells, and DMSO were used as controls. After 24 h, the medium of the culture was removed and replaced with 100 μL of DMEM without red phenol (Gibco–Thermo Fisher Scientific, Braunschweig, Germany) plus 10% heat-inactivated fetal bovine serum (Invitrogen, Karlsruhe, Germany), and 50 mM 2-mercaptoethanol (Gibco–Thermo Fisher Scientific, Braunschweig, Germany). Afterwards, the 12 mM MTT solution was added to each well according to the manufacturer’s instruction (Vybrant MTT Cell Proliferation Assay Kit, Thermo Fisher Scientific, Braunschweig, Germany). The OD value of each well was assayed at the wavelength of 570 nm on a microplate reader (TECAN Sunrise, Männedorf, Switzerland). The 50% cytotoxic concentration (CC_50_ values) of the tested natural products on Hs27 cells was calculated and all data were analyzed using Prism GraphPad version 9.2.0 software.

### 4.9. Determination of the Minimal Inhibitory Concentration against Different Pathogenic Bacteria

Testing for antibacterial activity was done as described previously [37]. Briefly, a single colony of Methicillin-resistant *Staphylococcus aureus* (MRSA strain Mu50, ATCC 700699) or *Pseudomonas aeruginosa* (strain PAO1, ATCC 87110) were grown in Mueller-Hinton broth (MHB) at 37 °C shaking at 120 rpm to reach an optical density of approx. 0.4. The cell suspension was adjusted to 10^6^ CFU/mL, of which 50 µL was seeded into a prepared 96-well polystyrene round-bottom plate containing test compounds diluted in MHB in a 1:1 serial dilution ranging from 100 µM to 0.78 µM. The plates were incubated at 37 °C statically for 24 h, and readout was performed using the BacTiter Glo assay (Promega) following the manufacturer’s instructions. Briefly, BacTiter Glo reagent was added to a white flat-bottom 96-well plate, and an equal volume of bacteria suspension was added to each well and mixed carefully. After 5 min, the luminescence was measured using a TECAN plate reader. The growth was calculated in regard to the vehicle (DMSO) and sterile control. Moxifloxacin and cefuroxime were used as a positive and negative control, respectively. All compounds were tested in triplicate.

For the testing against *M. tuberculosis* H37Rv, the Minimal Inhibitory Concentration (MIC) was determined in 96-well microtiter plates containing a total volume of 100 µL employing a resazurin reduction assay [41]. Briefly, a 96-well plate was prepared containing 7H9 medium supplemented with 10% ADS (0.81% NaCl, 5% BSA, 2% dextrose), 0.5% glycerol, and 0.05% tyloxapol. Compounds were two-fold serially diluted with the highest tested concentration of 100 µM. A *M. tuberculosis* culture was pre-grown to an OD_600 nm_ of approx. 0.4–0.6 by shaking at 37 °C in PETG square bottles (ThermoFisher Scientific, Braunschweig, Germany) containing 10 mL supplemented 7H9 medium. The cell density was adjusted to an OD_600 nm_ of 0.08 (10^6^ CFU/mL, and 5 × 10^4^ CFU were added to each well). Rifampicin and DMSO were used as a positive and solvent control, respectively. The 96-well plates were incubated for 5 days at 37 °C and 5% CO_2_ in humidified atmosphere. Afterwards, 10 µL of a 100 mg/mL resazurin solution was added to each well and resuspended carefully. After another 24 h at room temperature, the cells were fixed by adding 100 µL of a 10% formalin solution to each well. The readout was performed using a TECAN plate reader at 535 nm excitation and 590 nm emission. The growth was calculated in relation to the solvent control being 100% growth. The experiment was performed in triplicate.

### 4.10. Cytotoxicity Assay against Different Human Cell Lines

The cytotoxicity study was carried out using the THP-1 (human monocytic leukemia cell line), Huh-7 (Human liver carcinoma cell line), and HEK293 (human embryonic kidney cell line) cell lines as described before [37]. The THP-1 cells were cultured using RPMI 1640 medium containing 2 mM l-glutamine and supplemented with 10% fetal calf serum (FCS) and 1% sodium pyruvate. Huh-7 cells were cultured using a 1:1 mixture of RPMI 1640 medium containing 2 mM L-glutamine and 10% FCS medium and DMEM containing 10% FCS and 1% sodium pyruvate. The HEK-293 cells were cultured with DMEM including 2 mM l-glutamine and supplemented with 1% NE amino acids, 1% 1.0 mM sodium pyruvate and 10% FCS. All three cell lines were then incubated at 37 °C in an atmosphere of 5% CO_2_ under humid conditions for 2 weeks while renewing the medium twice weekly. Subsequently, the cells were suspended and adjusted to a density of 2 × 10^5^ cells/mL. In a 96-well flat-bottom microtiter plate, the cells were adjusted to a total volume of 100 μL containing 2-fold serial dilutions of the tested compounds **A**–**F** ranging from 100 to 1.56 μM. Cycloheximide (4, 2, 1, 0.5, 0.25, 0.13, 0.06, 0.03 µg/mL) was used as a positive control. After an incubation time of 48 h at 37 °C in an atmosphere of 5% CO_2_ under humid conditions, 10 μL resazurin solution (100 μg/mL) was added to each well and incubated for another 4 h. The fluorescence was then quantified using a Tecan Infinite 200pro microplate reader (excitation 540 nm, emission 590 nm). The residual growth was calculated relative to non-inoculated conditions (0% growth) and controls treated with DMSO (100% growth).

## Figures and Tables

**Table 1:** In vitro activity (IC_50_ values) of the natural compounds (**A**–**H**) from *P. selaginellae* against the *T. gondii* strain ME49. All experiments were conducted in triplicate.

Compound	IC_50_ (µM)
NK-A 17e233 (**A**)	5.75
1,2-benzenediol, 3-(4-hydroxy-2-methoxy-6-methylphenoxy)-5-methyl-(ACI) (**B**)	22.16
cyperin (**C**)	27.22
ES-242-1 (**D**)	7.38
ES-242-3 (**E**)	17.99
5*S*,6*S*-phomalactone (**F**)	5.13
methyltriaceticlactone (**G**)	Not active
S 39163/F-1 (**H**)	Not active
pyrimethamine	0.06

**Table 2:** In vitro cytotoxicity (CC_50_ values) of the natural compounds (**A**–**F**) from *P. selaginellae* against human fibroblasts Hs27. Concentration >100 µM indicates no activity in the experimental setup. All experiments were conducted in triplicate.

Compound	CC_50_ (µM)
**A**	>100
**B**	>100
**C**	>100
**D**	>100
**E**	>100
**F**	81
Pyrimethamine	44

**Table 3:** Mean IC_50_ values of compounds **A**–**F** against human cell lines THP-1, Huh-7, and Hek293. All concentrations are shown in µM. Concentration >100 µM indicates no activity in the experimental setup. All experiments were conducted in triplicate. The IC_50_ values were calculated using GraphPad Prism 7.

Compound	Mean IC_50_ [μM]		
	THP1	Huh-7	HEK-293
**A**	>100	>100	>100
**B**	>100	>100	>100
**C**	>100	>100	>100
**D**	>100	>100	93.8
**E**	21.9	13	16.95
**F**	24.3	100	66.9

**Table 4:** MIC_90_ against *S. aureus* ATCC 700699, *P. aeruginosa* ATCC 87110, and *M. tuberculosis* H37Rv. All concentrations are shown in µM. Concentration >100 µM indicates no activity in the experimental setup. All experiments were conducted in triplicate.

Compound	MIC_90_ [μM]		
	*S. aureus* ATCC 700699	*P. aeruginosa* ATCC 87110	*M. tuberculosis* H37Rv
**A**	>100	>100	>100
**B**	>100	>100	>100
**C**	>100	>100	>100
**D**	>100	>100	50
**E**	>100	>100	100
**F**	>100	100	>100
**G**	>100	>100	>100
**H**	>100	>100	>100

## Data Availability

All data presented in this study are contained within the article and the supplementary materials. The internal transcribed spacer (ITS) sequence for *Paraboeremia selaginellae* has been deposited in the National Center for Biotechnology Information (NCBI) GenBank under accession number ON231784.

## Supplementary Materials

The following supporting information can be downloaded at: https://www.mdpi.com/article/10.3390/antibiotics11091176/s1, Figures S1–S58: Spectroscopic data used for the structure elucidation of compounds A–H; Figure S59: Comparison of the experimental ECD spectrum of F measured in MeCN and the calculated ECD spectra of (5S,6S)-F computed at various levels of theory for the 10 lowest-energy ωB97X/TZVP PCM/MeCN conformers; Figure S60: Geometries of the low-energy ωB97X/TZVP PCM/MeCN conformers of (5S,6S)-F; Figure S61: Geometries of the low-energy B3LYP/TZVP PCM/CHCl3 conformers of (5S,6S)-F; Table S1: Boltzmann populations and specific optical rotations of the low-energy conformers of (5S,6S)-F computed at various levels for the low-energy ωB97X conformers. References [42,43,44,45] are cited in supplementary materials.

## References

[1] Frolich S., Entzeroth R., Wallach M. (2012). Comparison of protective immune responses to apicomplexan parasites. J. Parasitol. Res..

[2] Kim K., Weiss L.M. (2004). Toxoplasma gondii: The model apicomplexan. Int. J. Parasitol..

[3] (2009). Toxoplasmosis of Animals and Man. By J.P. Dubey and C. P. Beattie. 220 pages. ISBN 0 8493 4618 5. CRC Press, Boca Raton, 1988. £108.00. Parasitology.

[4] Dubey J.P. (2016). Toxoplasmosis of Animals and Humans.

[5] Saadatnia G., Golkar M. (2012). A review on human toxoplasmosis. Scand. J. Infect. Dis..

[6] Dubey J.P. (2021). Outbreaks of clinical toxoplasmosis in humans: Five decades of personal experience, perspectives and lessons learned. Parasites Vectors.

[7] Furtado J.M., Smith J.R., Belfort R., Gattey D., Winthrop K.L. (2011). Toxoplasmosis: A global threat. J. Glob. Infect. Dis..

[8] de Jong P.T. (1989). Ocular toxoplasmosis; common and rare symptoms and signs. Int. Ophthalmol..

[9] Elbez-Rubinstein A., Ajzenberg D., Dardé M.L., Cohen R., Dumètre A., Yera H., Gondon E., Janaud J.C., Thulliez P. (2009). Congenital toxoplasmosis and reinfection during pregnancy: Case report, strain characterization, experimental model of reinfection, and review. J. Infect. Dis..

[10] Dunay I.R., Gajurel K., Dhakal R., Liesenfeld O., Montoya J.G. (2018). Treatment of Toxoplasmosis: Historical Perspective, Animal Models, and Current Clinical Practice. Clin. Microbiol. Rev..

[11] Gopalakrishnan A.M., López-Estraño C. (2010). Comparative analysis of stage specific gene regulation of apicomplexan parasites: Plasmodium falciparum and Toxoplasma gondii. Infect. Disord. Drug Targets.

[12] Newman D.J., Cragg G.M. (2020). Natural Products as Sources of New Drugs over the Nearly Four Decades from 01/1981 to 09/2019. J. Nat. Prod..

[13] Cheraghipour K., Masoori L., Ezzatpour B., Roozbehani M., Sheikhian A., Malekara V., Niazi M., Mardanshah O., Moradpour K., Mahmoudvand H. (2021). The Experimental Role of Medicinal Plants in Treatment of Toxoplasma gondii Infection: A Systematic Review. Acta Parasitol..

[14] Lenzi J., Costa T.M., Alberton M.D., Goulart J.A.G., Tavares L.B.B. (2018). Medicinal fungi: A source of antiparasitic secondary metabolites. Appl. Microbiol. Biotechnol..

[15] Fukushima T., Tanaka M., Gohbara M., Fujimori T. (1998). Phytotoxicity of three lactones from Nigrospora sacchari. Phytochemistry.

[16] Evans R.H., Ellestad G.A., Kunstmann M.P. (1969). Two new metabolites from an unidentified nigrospora species. Tetrahedron Lett..

[17] Gusmao A.S., Abreu L.S., Tavares J.F., de Freitas H.F., Silva da Rocha Pita S., Dos Santos E.G., Caldas I.S., Vieira A.A., Silva E.O. (2021). Computer-Guided Trypanocidal Activity of Natural Lactones Produced by Endophytic Fungus of Euphorbia umbellata. Chem. Biodivers.

[18] Hussain H., Kock I., Al-Harrasi A., Al-Rawahi A., Abbas G., Green I.R., Shah A., Badshah A., Saleem M., Draeger S. (2014). Antimicrobial chemical constituents from endophytic fungus Phoma sp.. Asian Pac. J. Trop. Med..

[19] Khambay B.P.S., Bourne J.M., Cameron S., Kerry B.R., Zaki M.J. (2000). A nematicidal metabolite from Verticillium chlamydosporium. Pest Manag. Sci. Former. Pestic. Sci..

[20] Meepagala K.M., Johnson R.D., Techen N., Wedge D.E., Duke S.O. (2015). Phomalactone from a Phytopathogenic Fungus Infecting ZINNIA elegans (ASTERACEAE) Leaves. J. Chem. Ecol..

[21] Trisuwan K., Rukachaisirikul V., Sukpondma Y., Preedanon S., Phongpaichit S., Sakayaroj J. (2009). Pyrone derivatives from the marine-derived fungus *Nigrospora* sp. PSU-F18. Phytochemistry.

[22] Wu S.H., Chen Y.W., Shao S.C., Wang L.D., Yu Y., Li Z.Y., Yang L.Y., Li S.L., Huang R. (2009). Two new solanapyrone analogues from the endophytic fungus *Nigrospora* sp. YB-141 of Azadirachta indica. Chem. Biodivers.

[23] Mandi A., Kurtan T. (2019). Applications of OR/ECD/VCD to the structure elucidation of natural products. Nat. Prod. Rep..

[24] Szabo Z., Paczal A., Kovacs T., Mandi A., Kotschy A., Kurtan T. (2022). Synthesis and Vibrational Circular Dichroism Analysis of N-Heterocyclic Carbene Precursors Containing Remote Chirality Centers. Int. J. Mol. Sci.

[25] Atanasov A.G., Zotchev S.B., Dirsch V.M., Orhan I.E., Banach M., Rollinger J.M., Barreca D., Weckwerth W., Bauer R., Bayer E.A. (2021). Natural products in drug discovery: Advances and opportunities. Nat. Rev. Drug Discov..

[26] Guo H.-Y., Jin C., Zhang H.-M., Jin C.-M., Shen Q.-K., Quan Z.-S. (2019). Synthesis and Biological Evaluation of (+)-Usnic Acid Derivatives as Potential Anti-Toxoplasma gondii Agents. J. Agric. Food Chem..

[27] Jiménez-Romero C., Ortega-Barría E., Arnold A.E., Cubilla-Rios L. (2008). Activity against *Plasmodium falciparum* of Lactones Isolated from the Endophytic Fungus *Xylaria* sp.. Pharm. Biol..

[28] McMurry L.M., Oethinger M., Levy S.B. (1998). Triclosan targets lipid synthesis. Nature.

[29] McLeod R., Muench S.P., Rafferty J.B., Kyle D.E., Mui E.J., Kirisits M.J., Mack D.G., Roberts C.W., Samuel B.U., Lyons R.E. (2001). Triclosan inhibits the growth of Plasmodium falciparum and Toxoplasma gondii by inhibition of apicomplexan Fab I. Int. J. Parasitol..

[30] Tipparaju S.K., Muench S.P., Mui E.J., Ruzheinikov S.N., Lu J.Z., Hutson S.L., Kirisits M.J., Prigge S.T., Roberts C.W., Henriquez F.L. (2010). Identification and development of novel inhibitors of Toxoplasma gondii enoyl reductase. J. Med. Chem..

[31] Toki S., Ando K., Kawamoto I., Sano H., Yoshida M., Matsuda Y. (1992). ES-242-2, -3, -4, -5, -6, -7, and -8, novel bioxanthracenes produced by *Verticillium* sp., which act on the N-methyl-D-aspartate receptor. J. Antibiot..

[32] Jaturapat A., Isaka M., Hywel-Jones N.L., Lertwerawat Y., Kamchonwongpaisan S., Kirtikara K., Tanticharoen M., Thebtaranonth Y. (2001). Bioxanthracenes from the insect pathogenic fungus. Cordyceps pseudomilitaris BCC 1620. I. Taxonomy, fermentation, isolation and antimalarial activity. J. Antibiot..

[33] Komai S.-I., Hosoe T., Nozawa K., Okada K., de Campos Takaki G.M., Fukushima K., Miyaji M., Horie Y., Kawai K.-I. (2003). Antifungal activity of pyranone and furanone derivatives, isolated from *Aspergillus* sp. IFM51759, against *Aspergillus fumigatus*. MYCOTOXINS-TOKYO-.

[34] Krasnoff S.B., Gupta S. (1994). Identification of the antibiotic phomalactone from the entomopathogenic fungusHirsutella thompsonii var. synnematosa. J. Chem. Ecol..

[35] Krivobok S., Thomasson F., Seigle-Murandi F., Steiman R., Bottex-Gauthier C. (1994). 6-Allyl-5, 6-dihydro-5-hydroxypyran-2-one, a lactone produced by a new Drechslera species: Specified 1H and 13C NMR assignments, mutagenic and immunomodulating testings. Die Pharm..

[36] Kumarihamy M., Ferreira D., Croom E.M., Sahu R., Tekwani B.L., Duke S.O., Khan S., Techen N., Nanayakkara N.P.D. (2019). Antiplasmodial and Cytotoxic Cytochalasins from an Endophytic Fungus, *Nemania* sp. UM10M, Isolated from a Diseased *Torreya taxifolia* Leaf. Molecules.

[37] Meier D., Hernandez M.V., van Geelen L., Muharini R., Proksch P., Bandow J.E., Kalscheuer R. (2019). The plant-derived chalcone Xanthoangelol targets the membrane of Gram-positive bacteria. Bioorg Med. Chem..

[38] Pfefferkorn E.R., Pfefferkorn L.C. (1977). Specific Labeling of Intracellular Toxoplasma gondii with Uracil. J. Protozool..

[39] Mosmann T. (1983). Rapid colorimetric assay for cellular growth and survival: Application to proliferation and cytotoxicity assays. J. Immunol. Methods.

[40] Tamaoki T., Nomoto H., Takahashi I., Kato Y., Morimoto M., Tomita F. (1986). Staurosporine, a potent inhibitor of phospholipid/Ca++dependent protein kinase. Biochem. Biophys. Res. Commun..

[41] Rehberg N., Akone H.S., Ioerger T.R., Erlenkamp G., Daletos G., Gohlke H., Proksch P., Kalscheuer R. (2018). Chlorflavonin Targets Acetohydroxyacid Synthase Catalytic Subunit IlvB1 for Synergistic Killing of *Mycobacterium tuberculosis*. ACS Infect. Dis..

[42] (2015). MacroModel; Schrödinger LLC. http://www.schrodinger.com/MacroModel.

[43] Frisch M.J., Trucks G.W., Schlegel H.B., Scuseria G.E., Robb M.A., Cheeseman J.R., Scalmani V., Barone G., Mennucci B., Petersson G.A. (2013). Gaussian 09 (Revision E.01).

[44] Stephens P.J., Harada N. (2009). ECD cotton effect approximated by the Gaussian curve and other methods. Chirality.

[45] Varetto U. (2009). Molekel 5.4.

