# Supplementary material for: In Vitro Biological Activity of Natural Products from the Endophytic Fungus Paraboeremia selaginellae against Toxoplasma gondii
Source: Antibiotics (Basel). 2022 Aug 31;11(9):1176. doi: 10.3390/antibiotics11091176 (PMC9495004; doi:10.3390/antibiotics11091176)
Supplement: Supplementary file 1 [file antibiotics-11-01176-s001.zip › antibiotics-1888462-supplementary.pdf]

Supplementary Materials:

Figure **S1** ^{1}H-NMR spectrum (DMSO-d6 – 600 MHz) of Compound A……4
Figure **S2.** COSY NMR spectrum (DMSO-d6 – 600 MHz) of compound A……5
Figure **S3.** HMBC NMR spectrum (DMSO-d6 – 600 MHz) of compound A……6
Figure **S4.** HSQC NMR spectrum (DMSO-d6 – 600 MHz) of compound A……7
Figure **S5.** NOESY NMR spectrum (DMSO-d6 – 600 MHz) of compound A……8
Figure **S6.** ^{1}H NMR spectrum ($CDCl_3$ - 600 MHz) of compound B……9
Figure **S7.** COSY NMR spectrum ($CDCl_3$ - 600 MHz) of compound B……10
Figure **S8.** HMBC NMR spectrum ($CDCl_3$ - 600 MHz) of compound B……11
Figure **S9.** NOESY NMR spectrum ($CDCl_3$ - 600 MHz) of compound B……12
Figure **S10.** ^{1}H NMR spectrum ($CDCl_3$ - 600 MHz) of compound C……13
Figure **S11.** COSY NMR spectrum ($CDCl_3$ - 600 MHz) of compound C……14
Figure **S12.** HMBC NMR spectrum ($CDCl_3$ - 600 MHz) of compound C……15
Figure **S13.** NOESY NMR spectrum ($CDCl_3$ - 600 MHz) of compound C……16
Figure **S14.** ^{1}H NMR spectrum ($CDCl_3$ - 600 MHz) of compound D……17
Figure **S15.** ^{13}C NMR spectrum ($CDCl_3$ - 150 MHz) of compound D……18
Figure **S16.** COSY NMR spectrum ($CDCl_3$ - 600 MHz) of compound D……19
Figure **S17.** HSQC NMR spectrum ($CDCl_3$ - 600 MHz) of compound D……20
Figure **S18.** HMBC NMR spectrum ($CDCl_3$ - 600 MHz) of compound D……21
Figure **S19.** ^{1}H NMR spectrum ($CDCl_3$ - 600 MHz) of compound E……22
Figure **S20.** ^{13}C NMR spectrum ($CDCl_3$ - 150 MHz) of compound E……23
Figure **S21.** COSY NMR spectrum ($CDCl_3$ - 600 MHz) of compound E……24
Figure **S22.** HSQC NMR spectrum ($CDCl_3$ - 600 MHz) of compound E……25
Figure **S23.** HMBC NMR spectrum ($CDCl_3$ - 600 MHz) of compound E……26
Figure **S24.** ^{1}H NMR spectrum ($CDCl_3$ - 600 MHz) of compound F……27

Figure **S25.** ^{13}C NMR spectrum ($CDCl_3$ - 150 MHz) of compound F……28
Figure **S26.** COSY NMR spectrum ($CDCl_3$ - 600 MHz) of compound F……29
Figure **S27.** HSQC NMR spectrum ($CDCl_3$ - 600 MHz) of compound F……30
Figure **S28.** HMBC NMR spectrum ($CDCl_3$ - 600 MHz) of compound F……31
Figure **S29.** ^{1}H NMR spectrum (DMSO-d6 – 600 MHz) of compound G……32
Figure **S30.** ^{13}C NMR spectrum (DMSO-d6 – 150 MHz) of compound G……33
Figure **S31.** COSY NMR spectrum (DMSO-d6 – 600 MHz) of compound G……34
Figure **S32.** HSQC NMR spectrum (DMSO-d6 – 600 MHz) of compound G……35
Figure **S33.** HMBC NMR spectrum (DMSO-d6 – 600 MHz) of compound G……36
Figure **S34.** ^{1}H NMR spectrum ($CDCl_3$ - 600 MHz) of compound H……37
Figure **S35.** HRESIMS (MeOH) of compound A……38
Figure **S36.** HRESIMS (MeOH) of compound B……39
Figure **S37.** HRESIMS (MeOH) of compound C……40
Figure **S38.** HRESIMS (MeOH) of compound D……41
Figure **S39.** HRESIMS (MeOH) of compound E……42
Figure **S40.** HRESIMS (MeOH) of compound F……43
Figure **S41.** HRESIMS (MeOH) of compound G……44
Figure **S42.** HRESIMS (MeOH) of compound H……45
Figure **S43.** HPLC chromatogram (MeOH) of compound A……46
Figure **S44.** UV-Vis spectrum (MeOH) of compound A……46
Figure **S45.** HPLC chromatogram (MeOH) of compound B……47
Figure **S46.** UV-Vis spectrum (MeOH) of compound B……47
Figure **S47.** HPLC chromatogram (MeOH) of compound C……48
Figure **S48.** UV-Vis spectrum (MeOH) of compound C……48
Figure **S49.** HPLC chromatogram (MeOH) of compound D……49
Figure **S50.** UV-Vis spectrum (MeOH) of compound D……49
Figure **S51.** HPLC chromatogram of (MeOH) compound E……50
Figure **S52.** UV-Vis spectrum (MeOH) of compound E……50

Figure **S53.** HPLC chromatogram (MeOH) of compound F……………………………………………………………………………………………………51
Figure **S54.** UV-Vis spectrum (MeOH) of compound F……51
Figure **S55.** HPLC chromatogram (MeOH) of compound G……………………………………………………………………………………………………52
Figure **S56.** UV-Vis spectrum (MeOH) of compound G………………………………………………………………………………………………………...52
Figure **S57-** HPLC-chromatogram (MeOH) of compound H…………………………………………………………………………………………………..53
Figure **S58.** UV-Vis spectrum (MeOH) of compound H………………………………………………………………………………………………………..53
Stereochemical Studies of compound F……….54-58

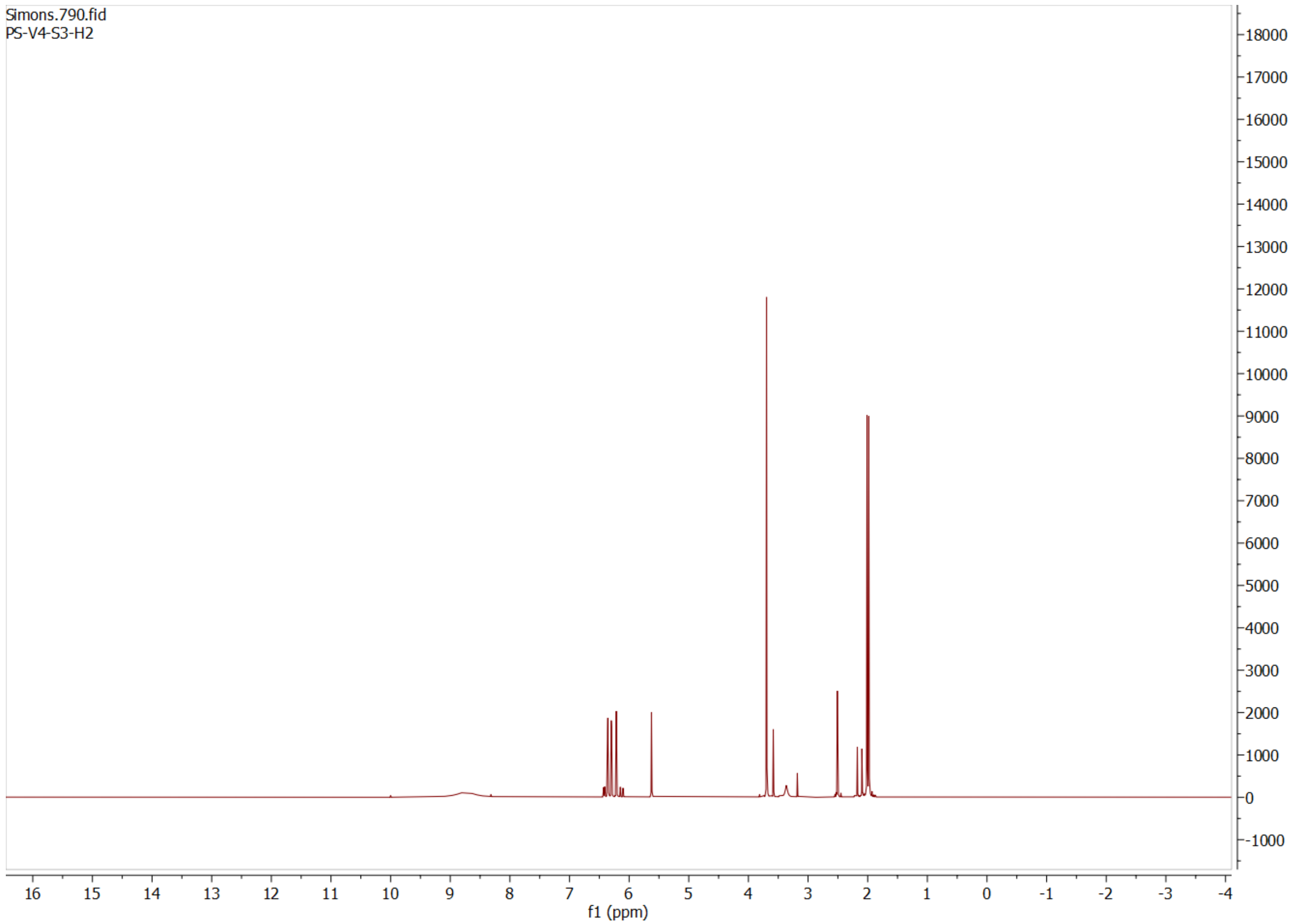

Figure **S1.** ^{1}H-NMR spectrum (DMSO-d6 – 600 MHz) of Compound A

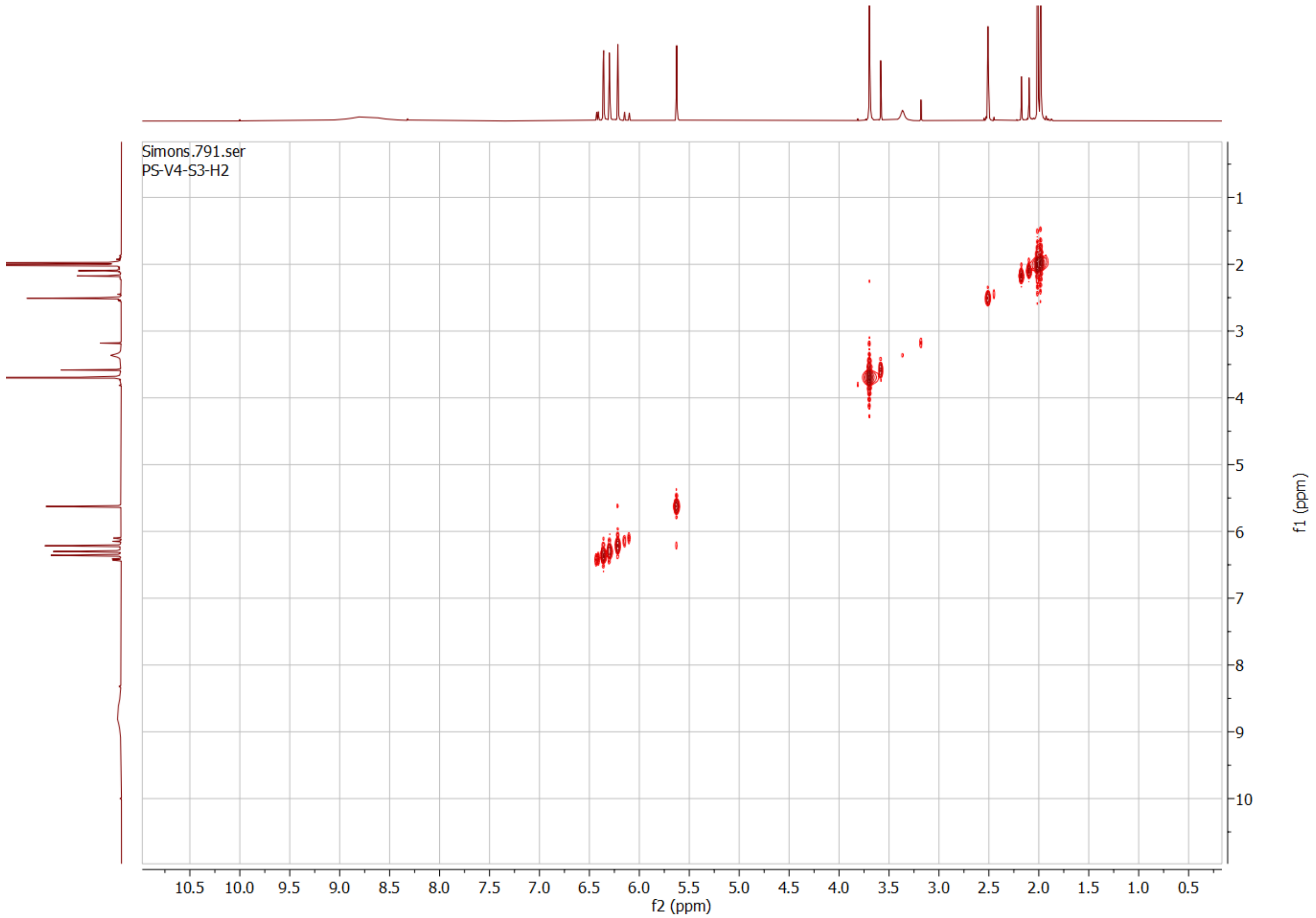

Figure **S2.** COSY NMR spectrum (DMSO-d6 – 600 MHz) of compound A

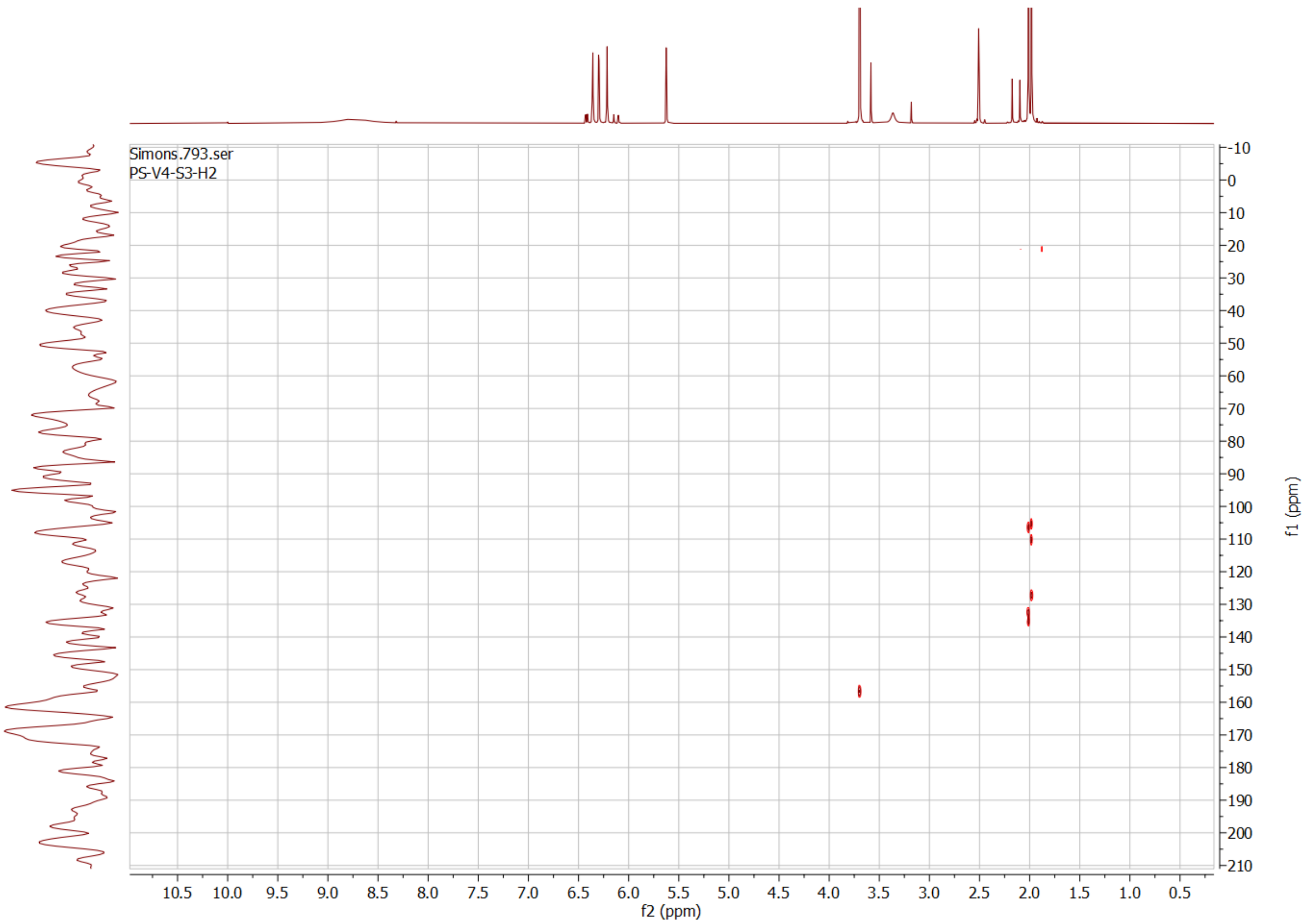

Figure **S3.** HMBC NMR spectrum (DMSO-d6 – 600 MHz) of compound A

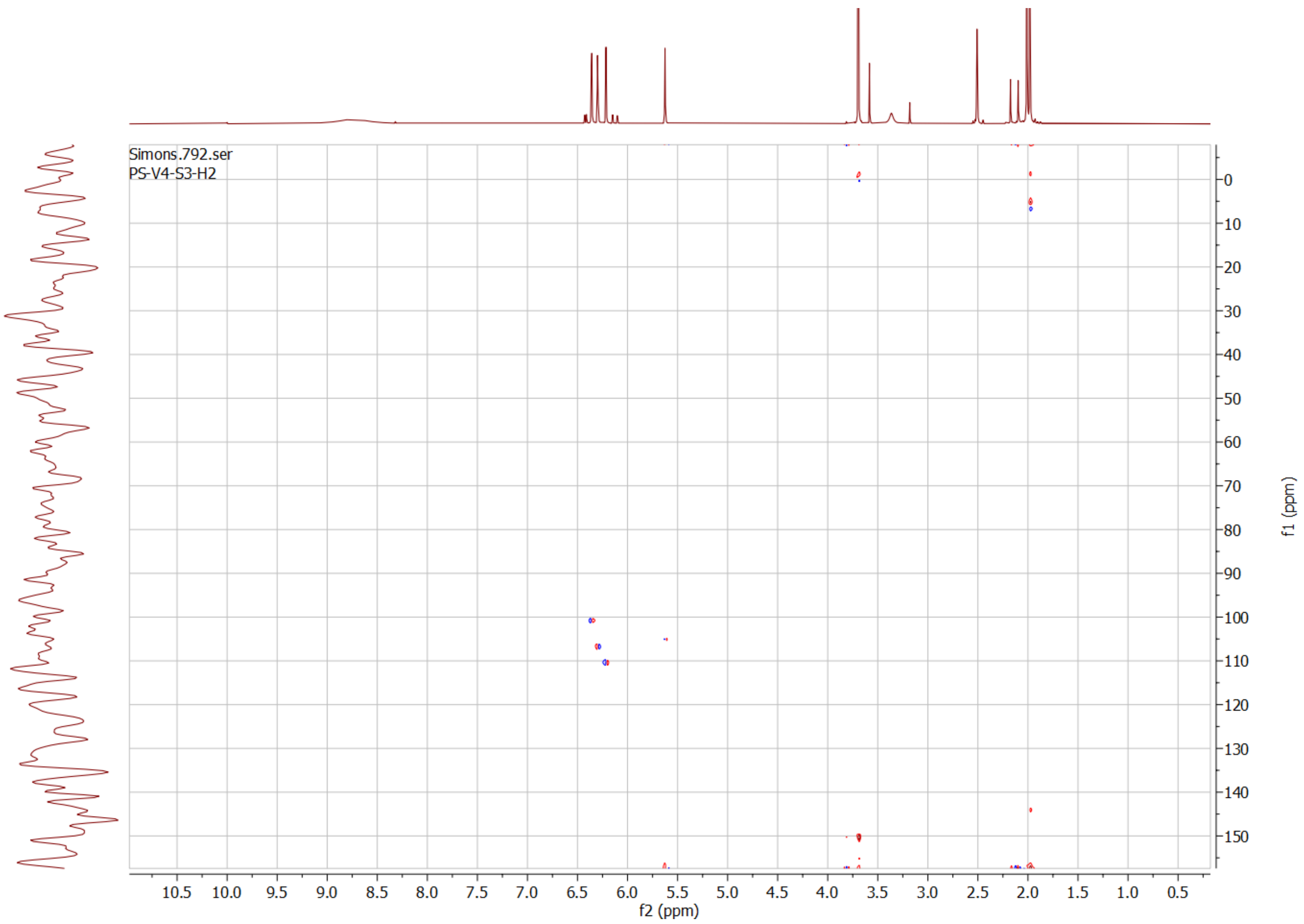

Figure **S4.** HSQC NMR spectrum (DMSO-d6 – 600 MHz) of compound A

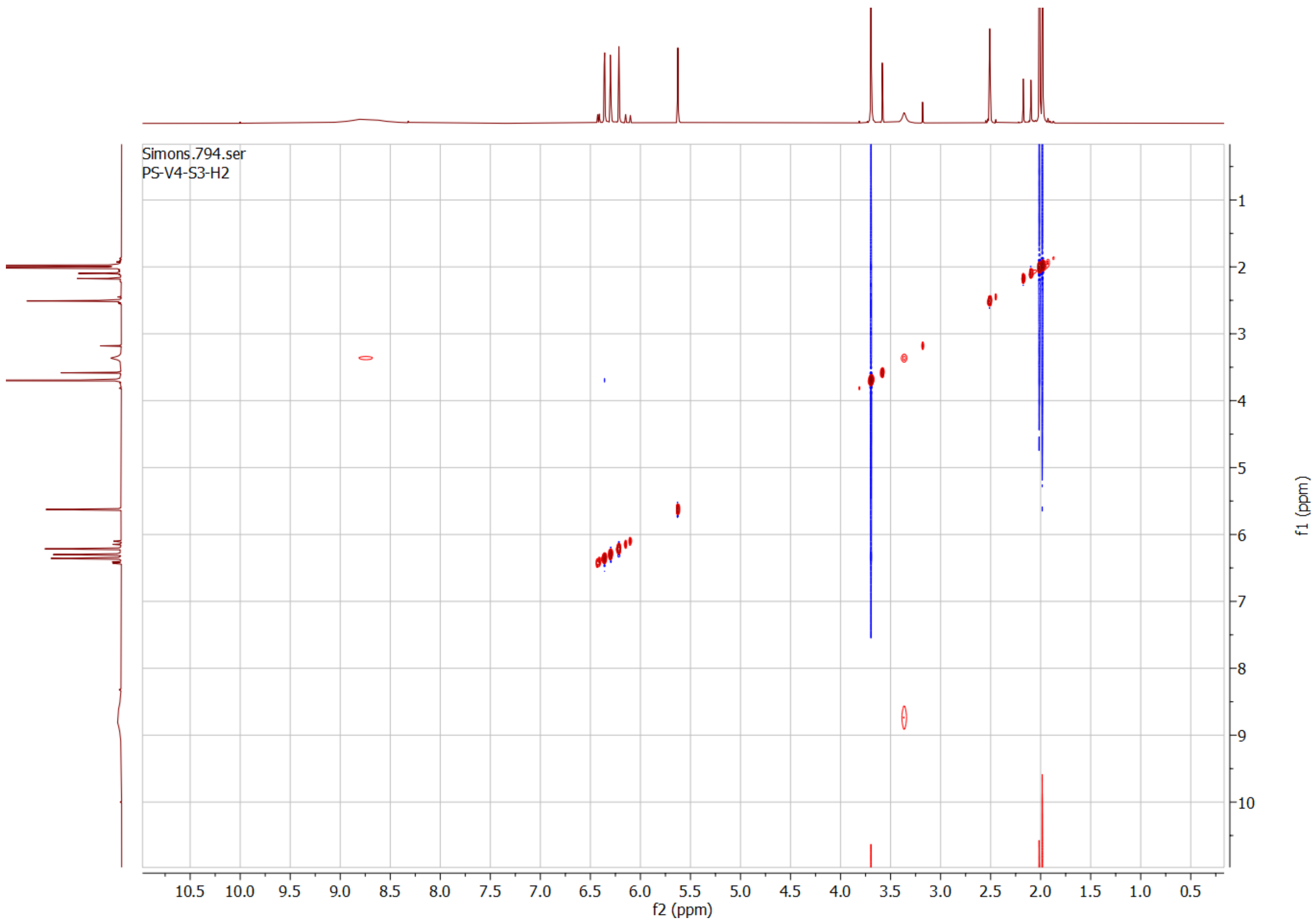

Figure **S5.** NOESY NMR spectrum (DMSO-d6 – 600 MHz) of compound A

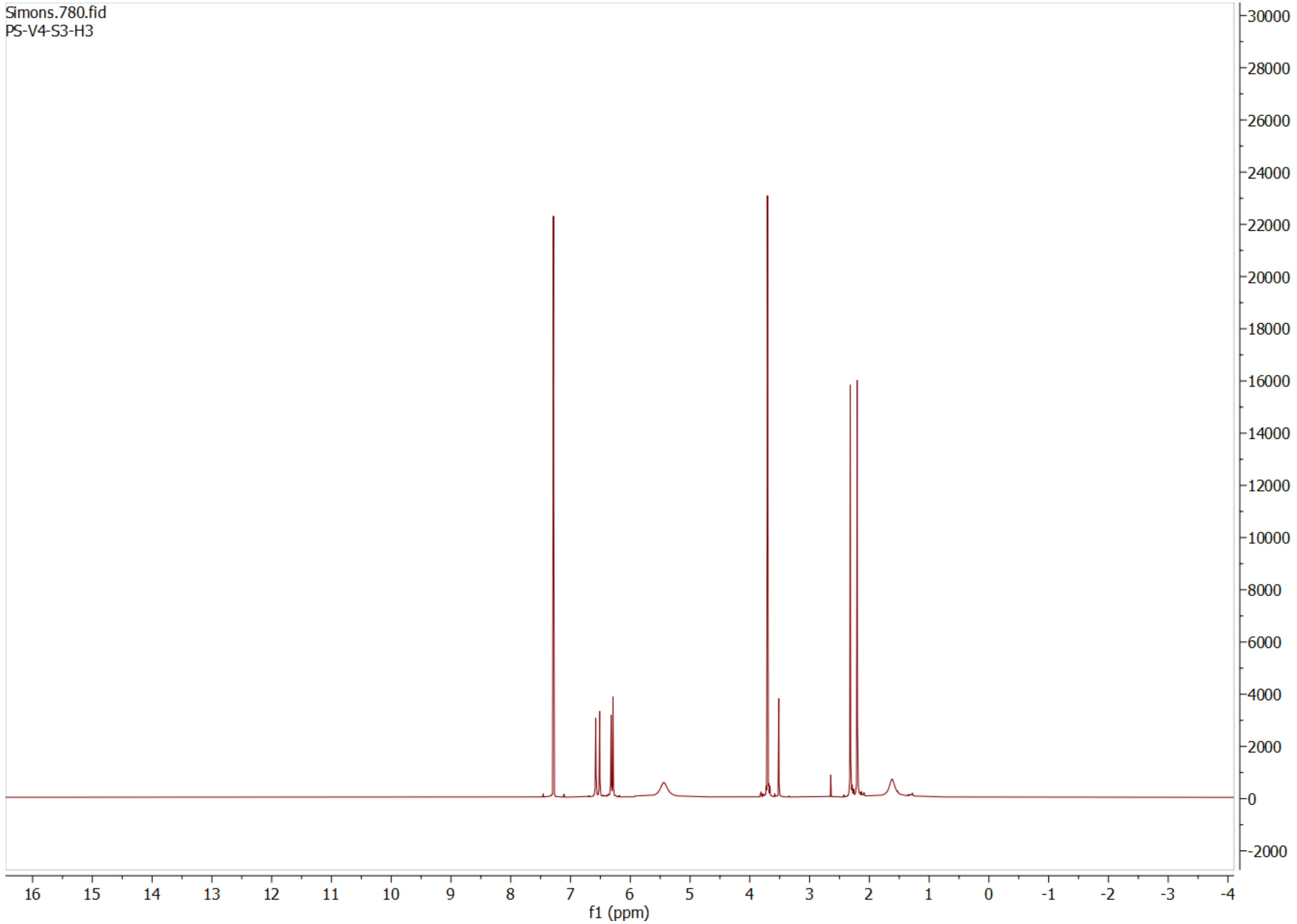

Figure **S6.** ^{1}H NMR spectrum ($CDCl_3$ - 600 MHz) of compound B

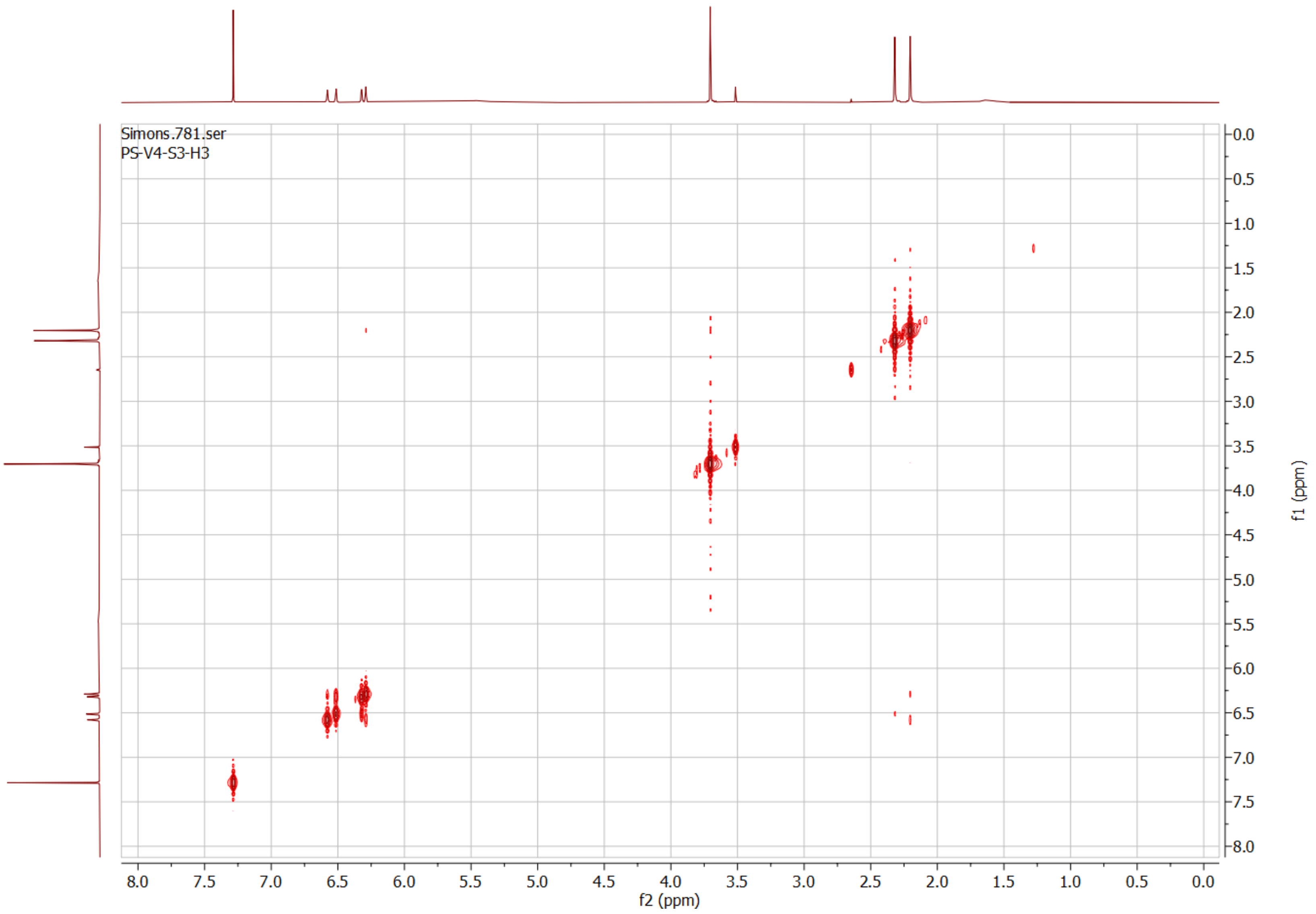

Figure **S7.** COSY NMR spectrum ($CDCl_3$ - 600 MHz) of compound B

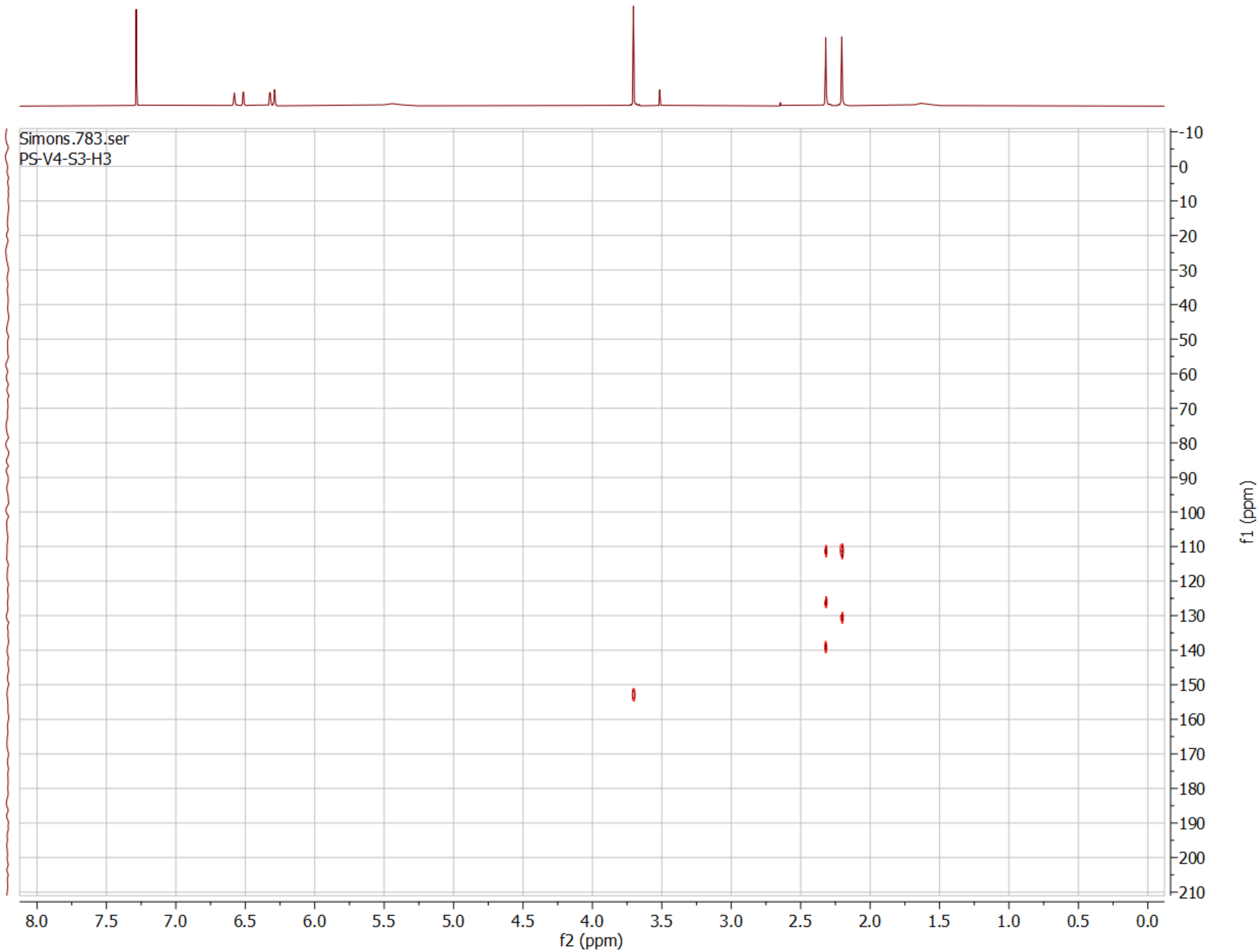

Figure **S8.** HMBC NMR spectrum ($CDCl_3$ - 600 MHz) of compound B

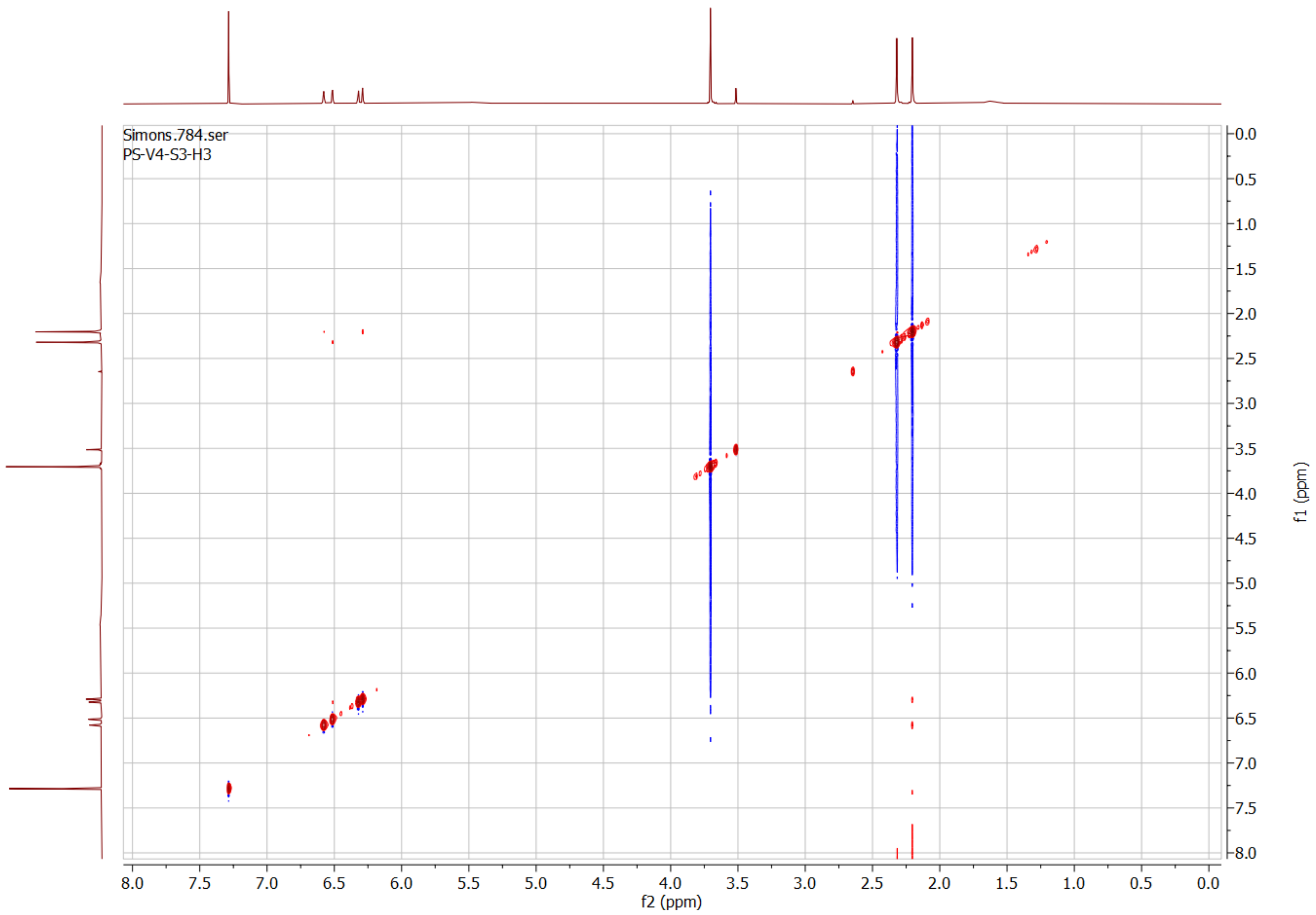

Figure **S9.** NOESY NMR spectrum ($CDCl_3$ - 600 MHz) of compound B

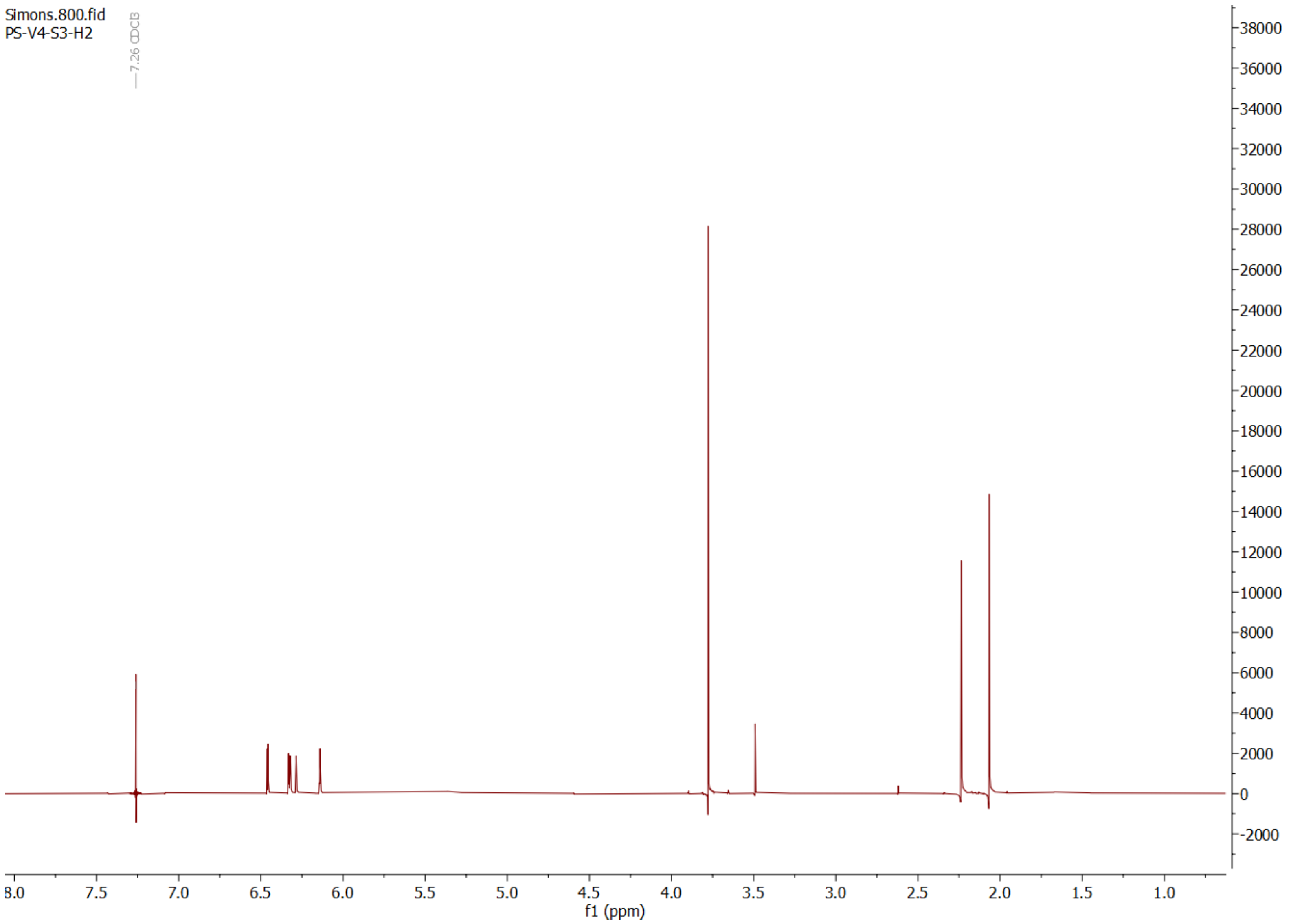

Figure **S10.** ^{1}H NMR spectrum ($CDCl_3$ - 600 MHz) of compound C

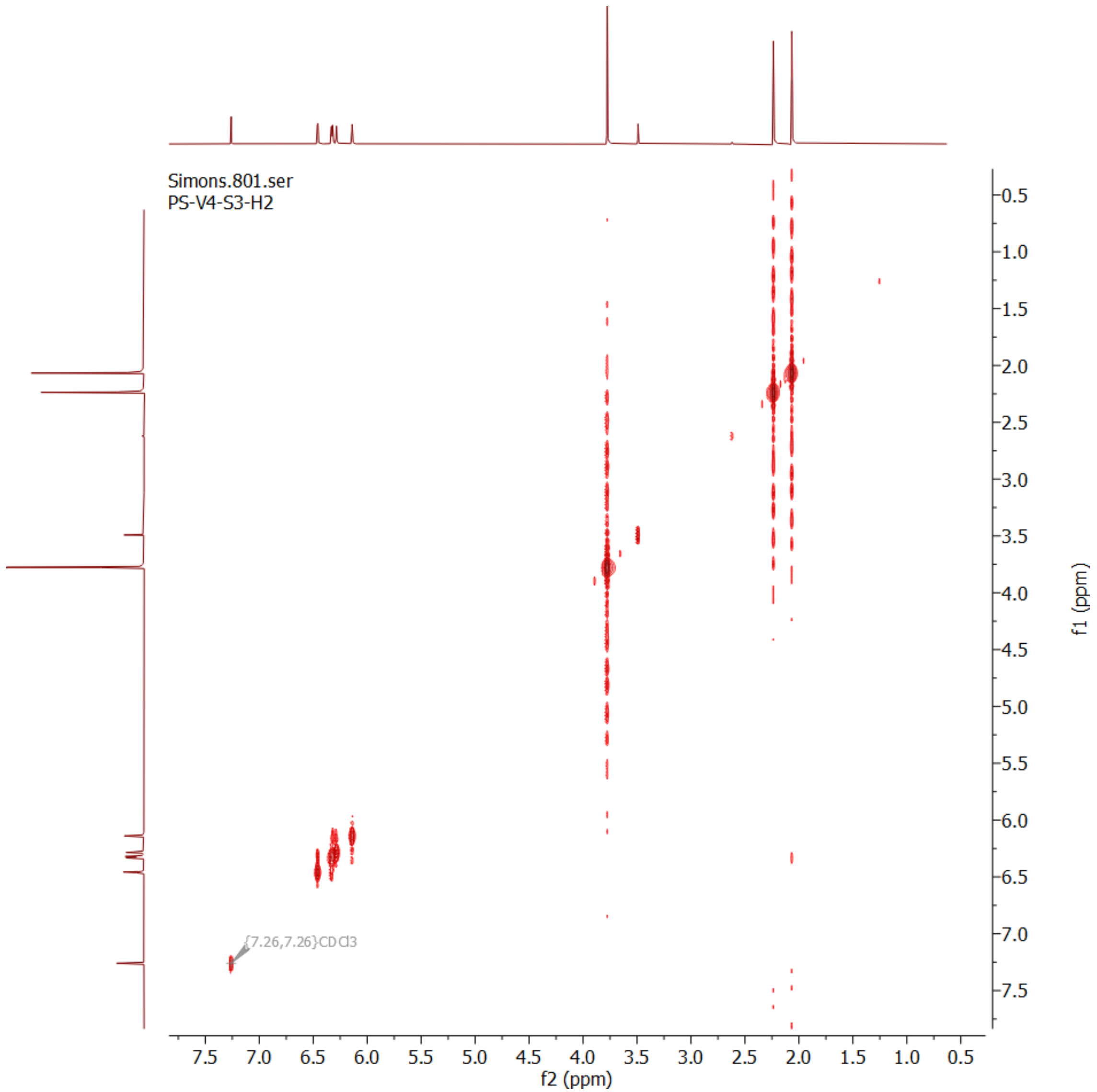

Figure **S11.** COSY NMR spectrum ($CDCl_3$ - 600 MHz) of compound C

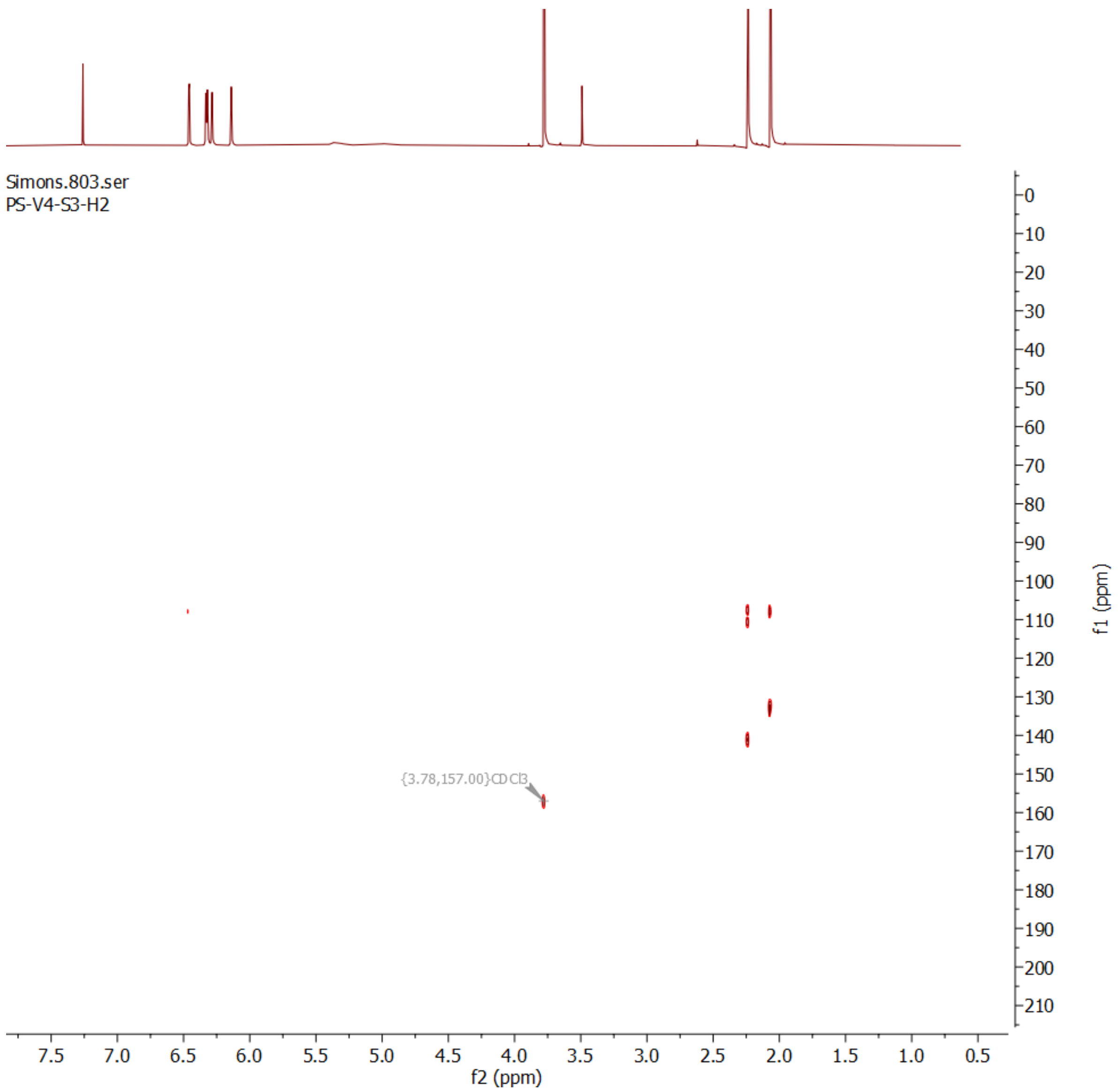

Figure **S12.** HMBC NMR spectrum ($CDCl_3$ - 600 MHz) of compound C

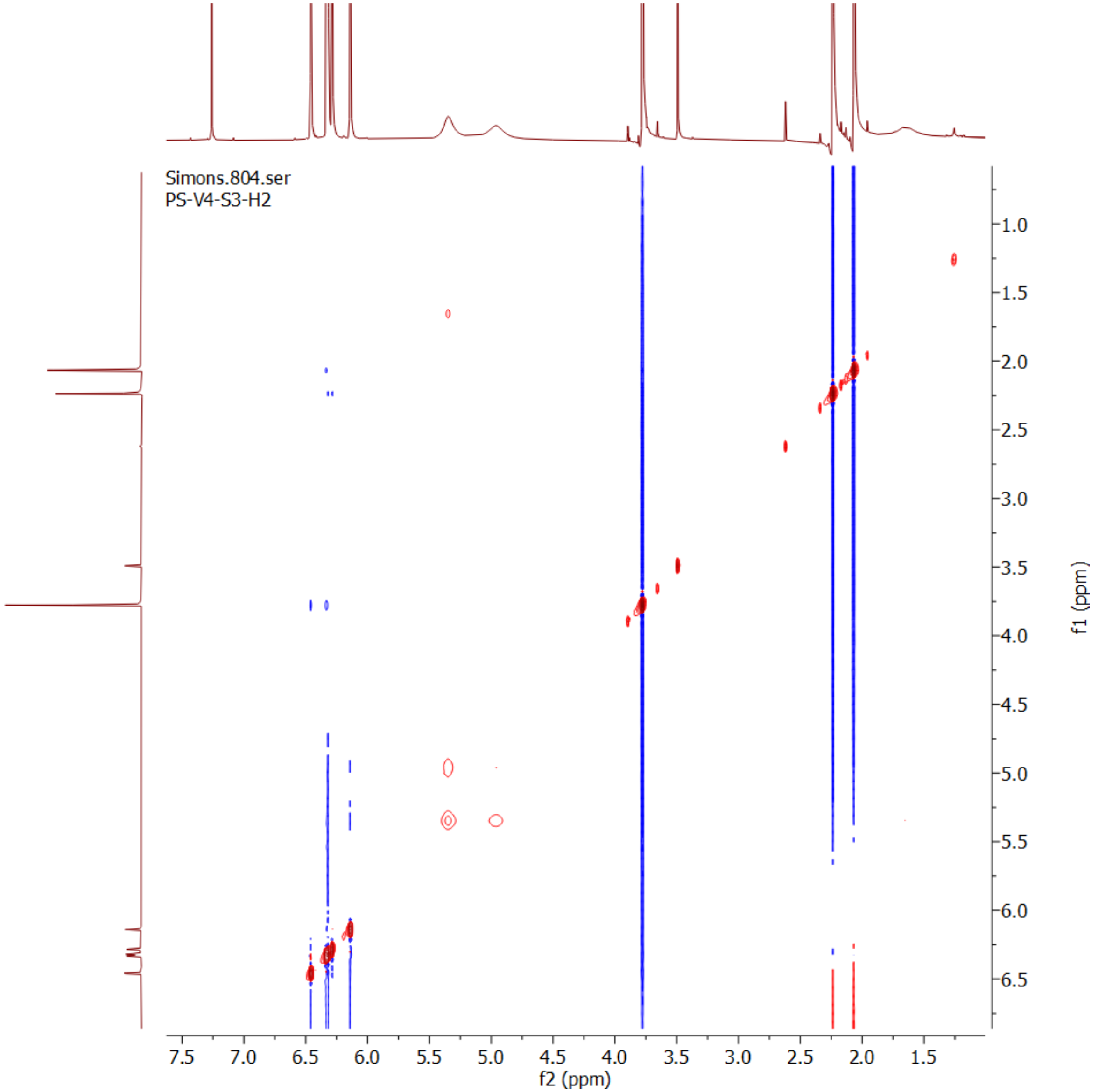

Figure **S13.** NOESY NMR spectrum ($CDCl_3$ - 600 MHz) of compound C

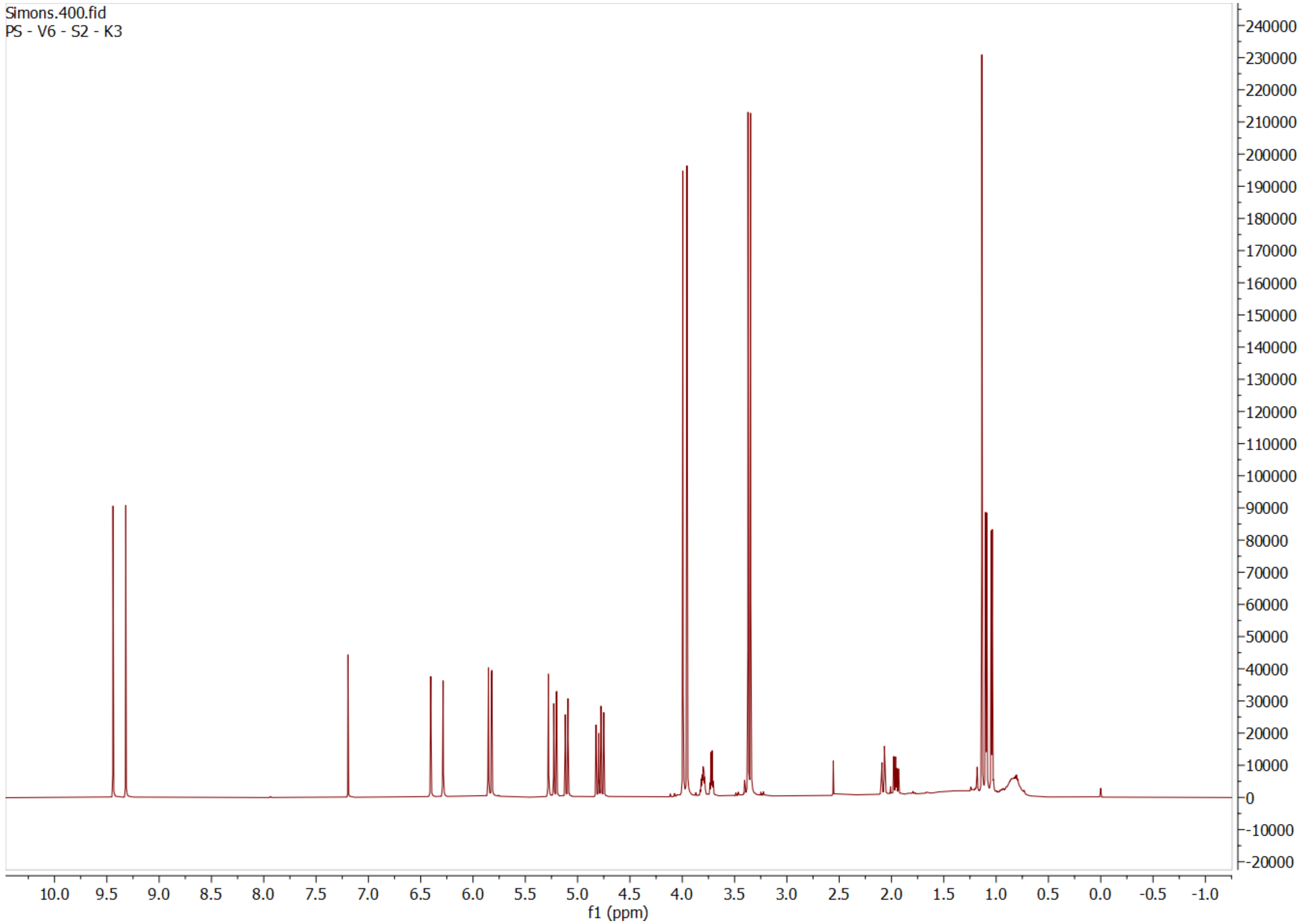

Figure S14. ^{1}H NMR spectrum ($CDCl_3$ - 600 MHz) of compound D

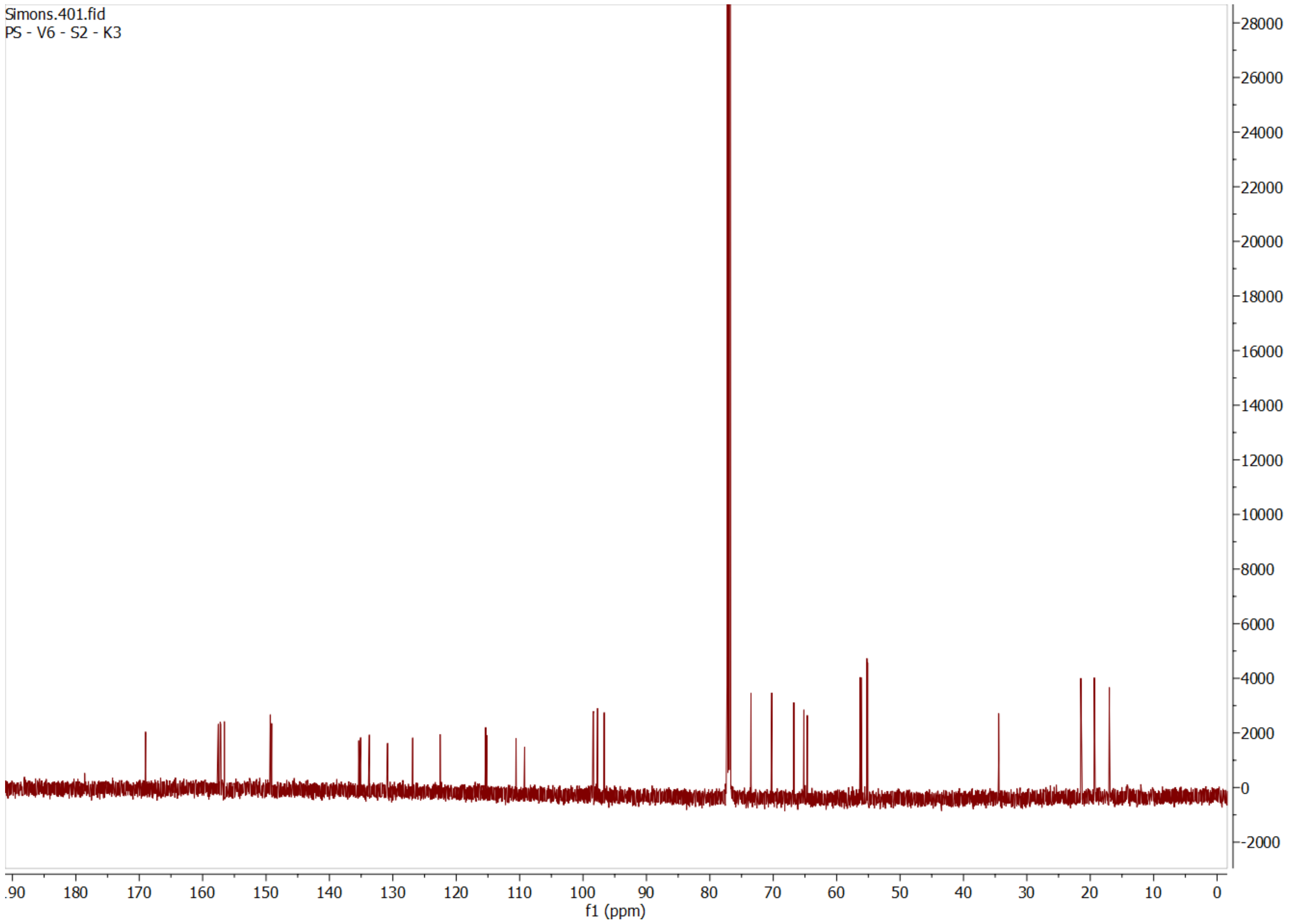

Figure **S15.** ^{13}C NMR spectrum ($CDCl_3$ - 150 MHz) of compound D

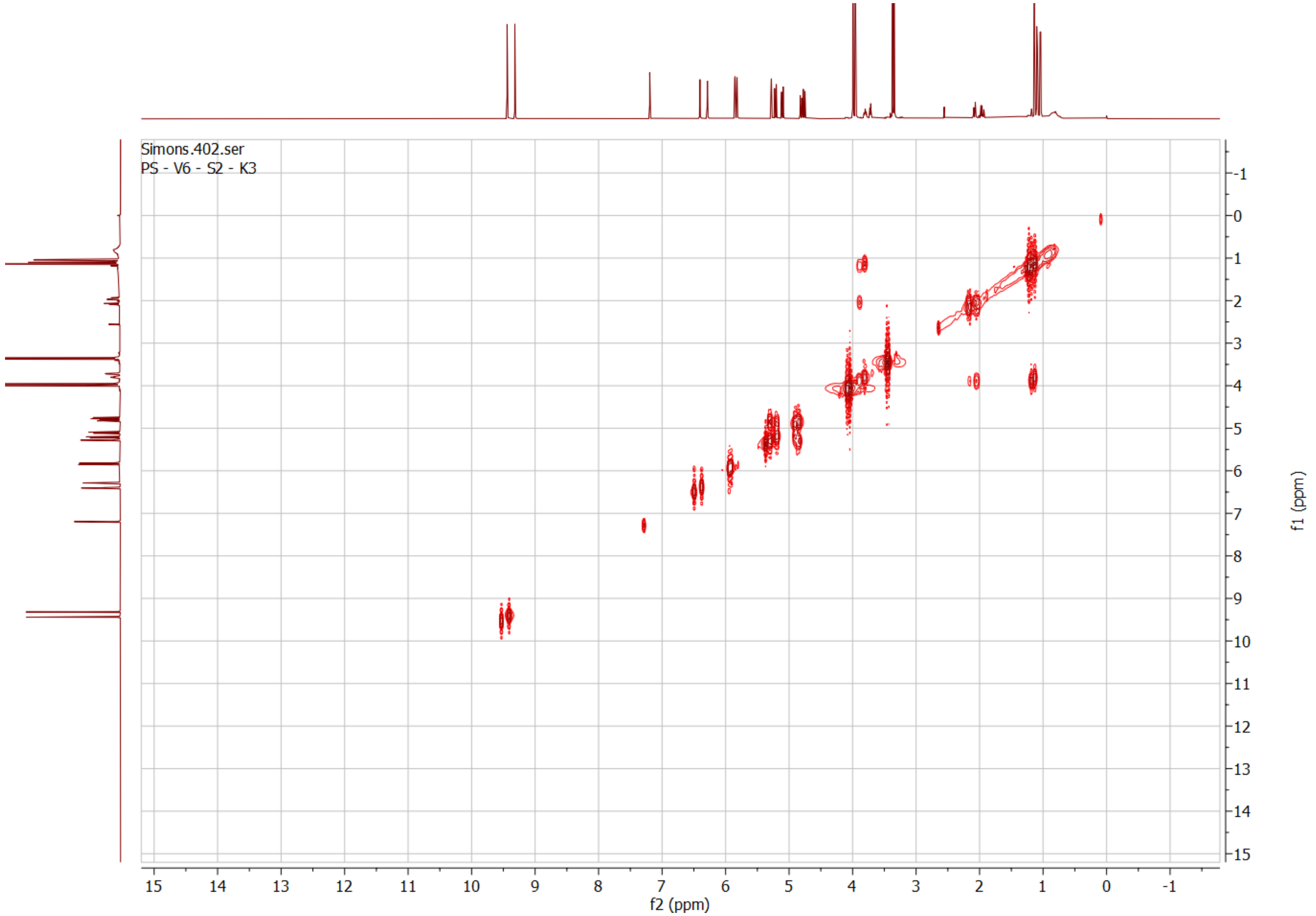

Figure **S16.** COSY NMR spectrum ($CDCl_3$ - 600 MHz) of compound D

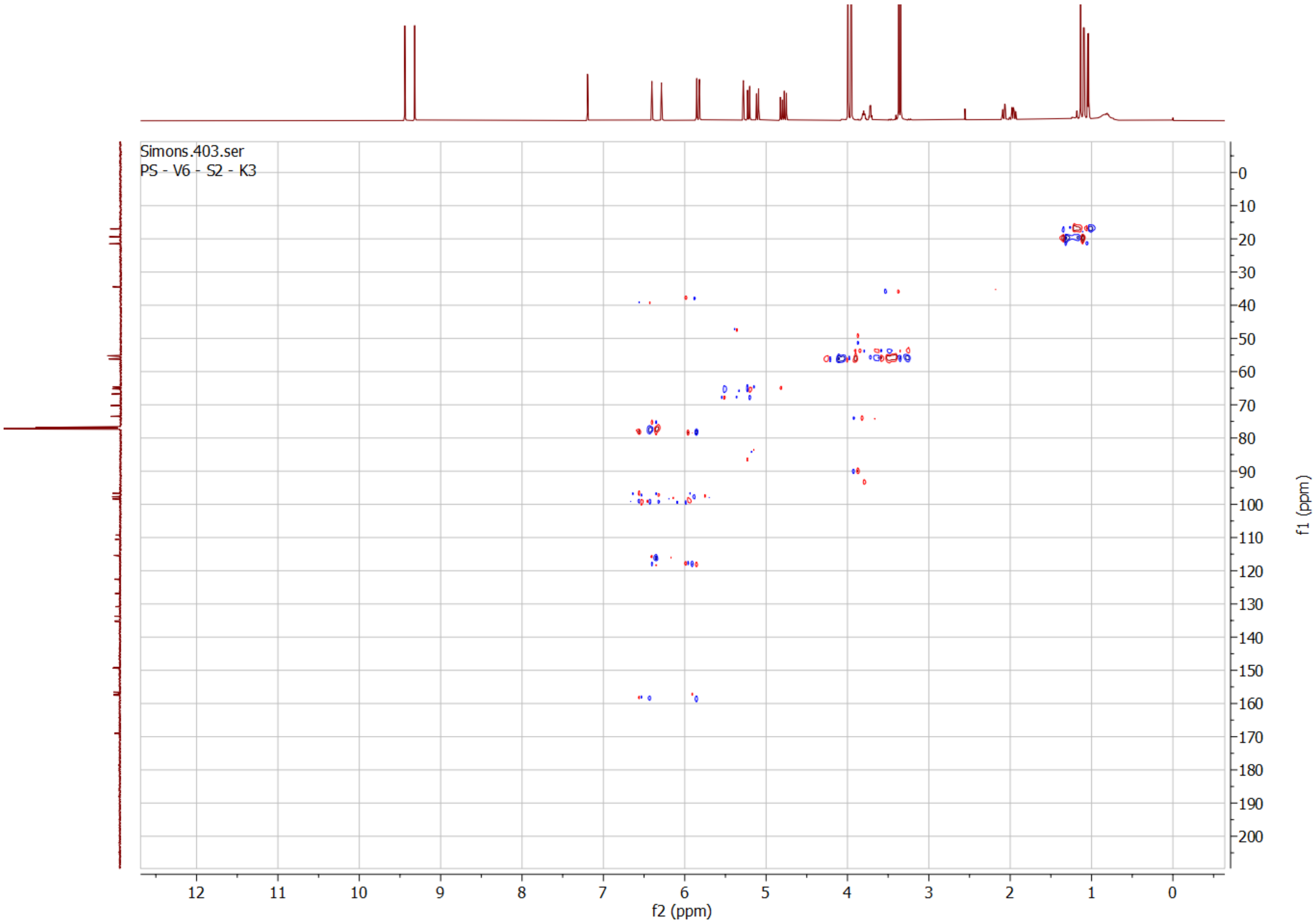

Figure **S17.** HSQC NMR spectrum ($CDCl_3$ - 600 MHz) of compound D

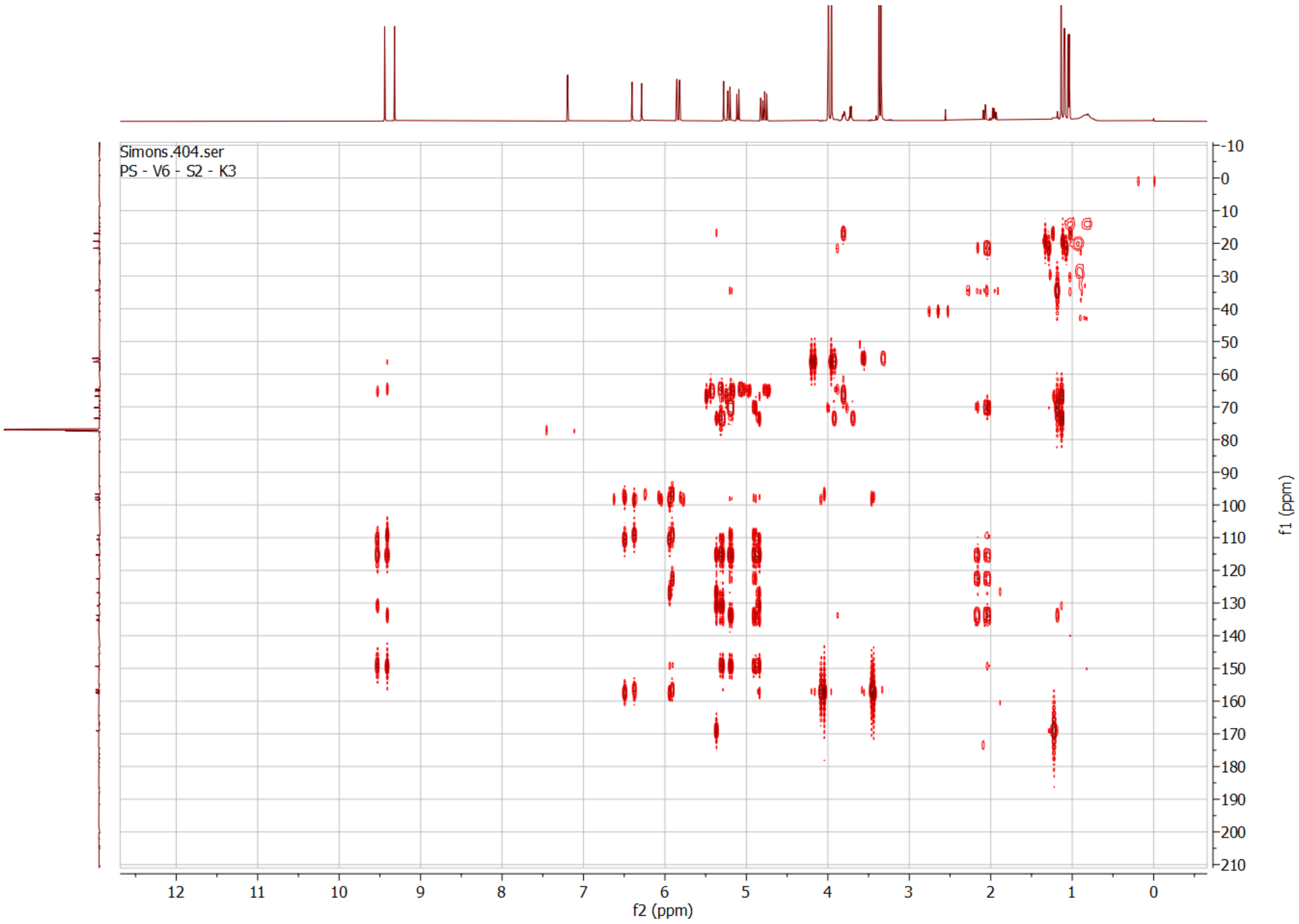

Figure **S18.** HMBC NMR spectrum ($CDCl_3$ - 600 MHz) of compound D

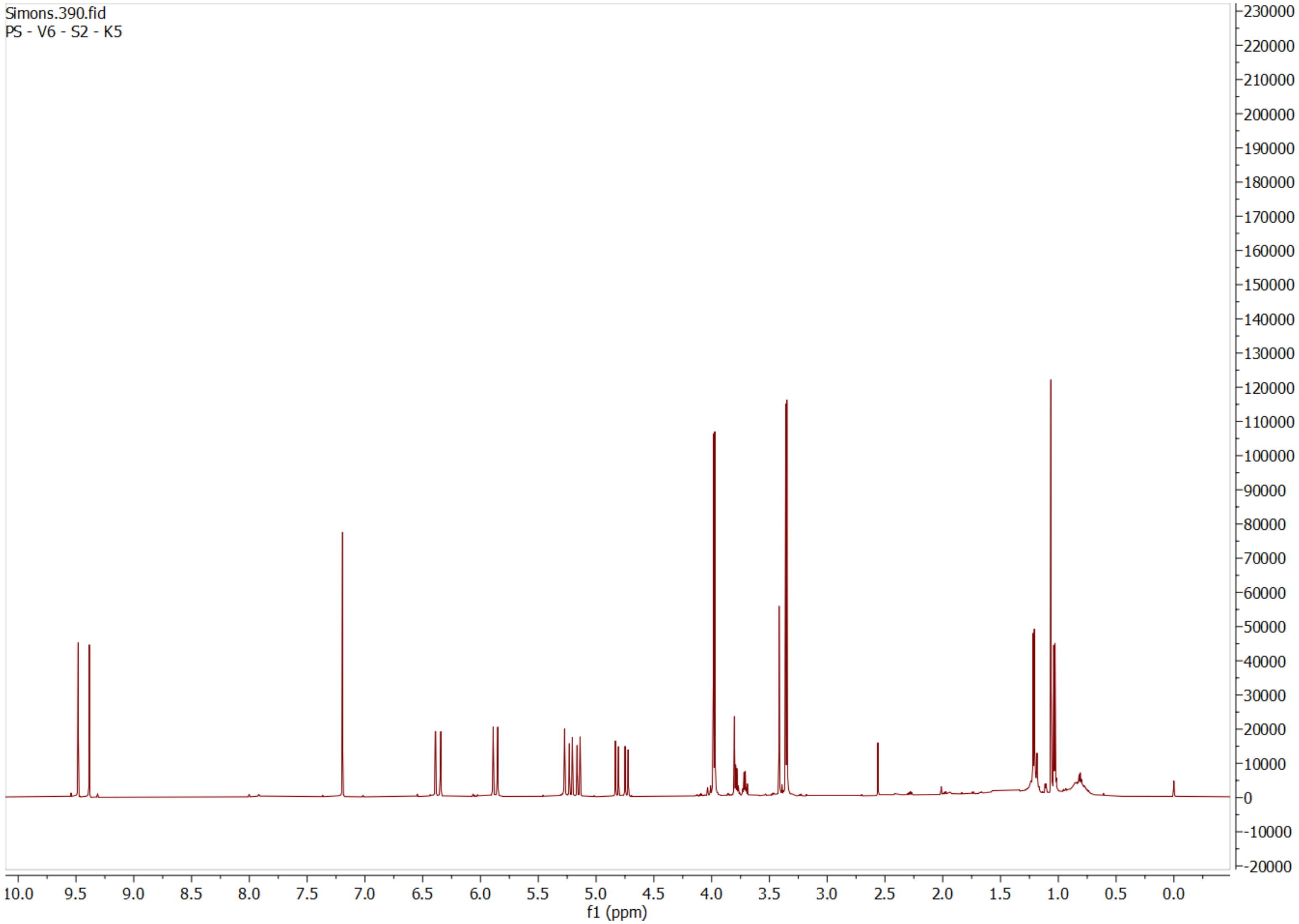

Figure **S19.** ^{1}H NMR spectrum ($CDCl_3$ - 600 MHz) of compound E

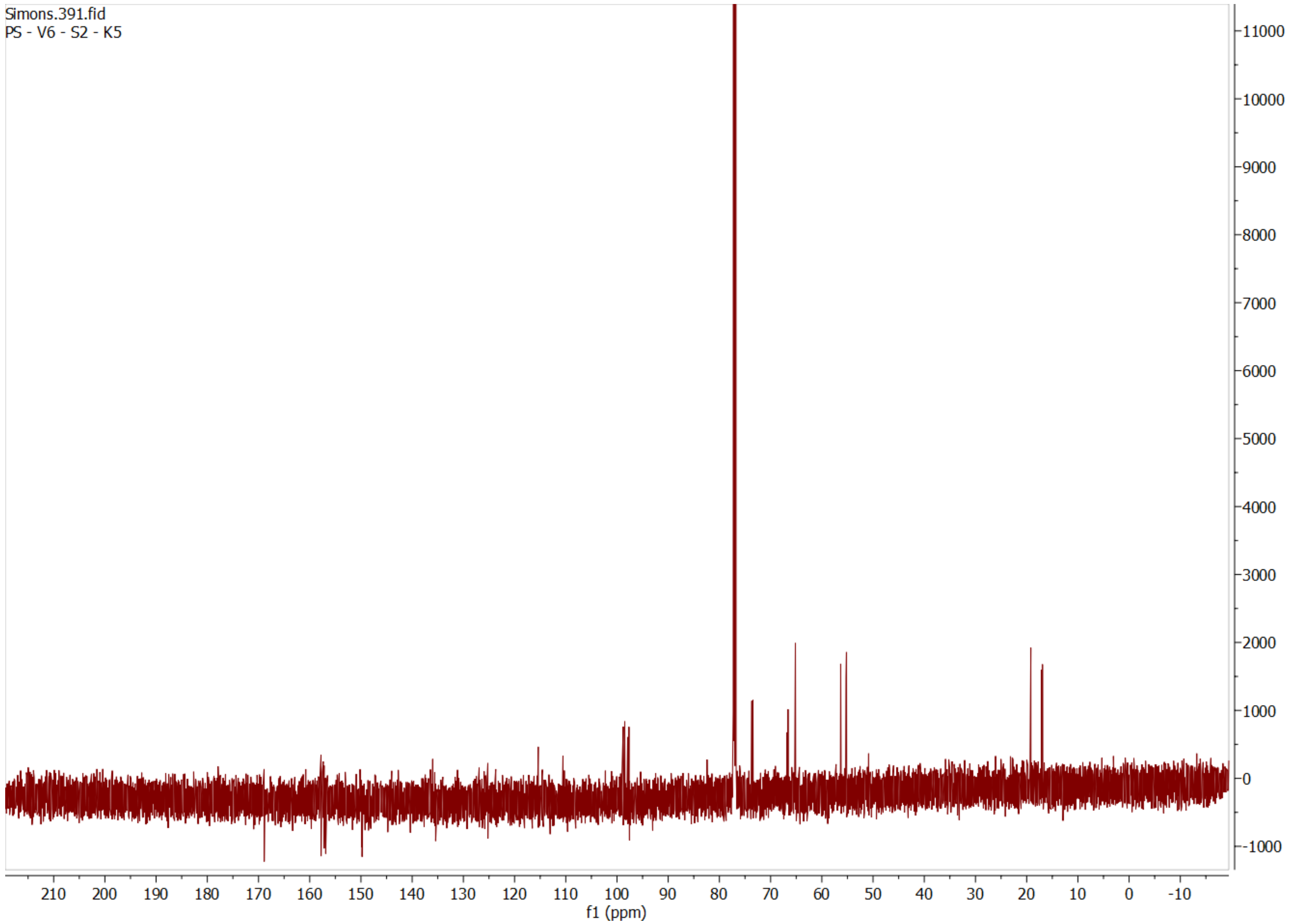

Figure **S20.** ^{13}C NMR spectrum ($CDCl_3$ - 150 MHz) of compound E

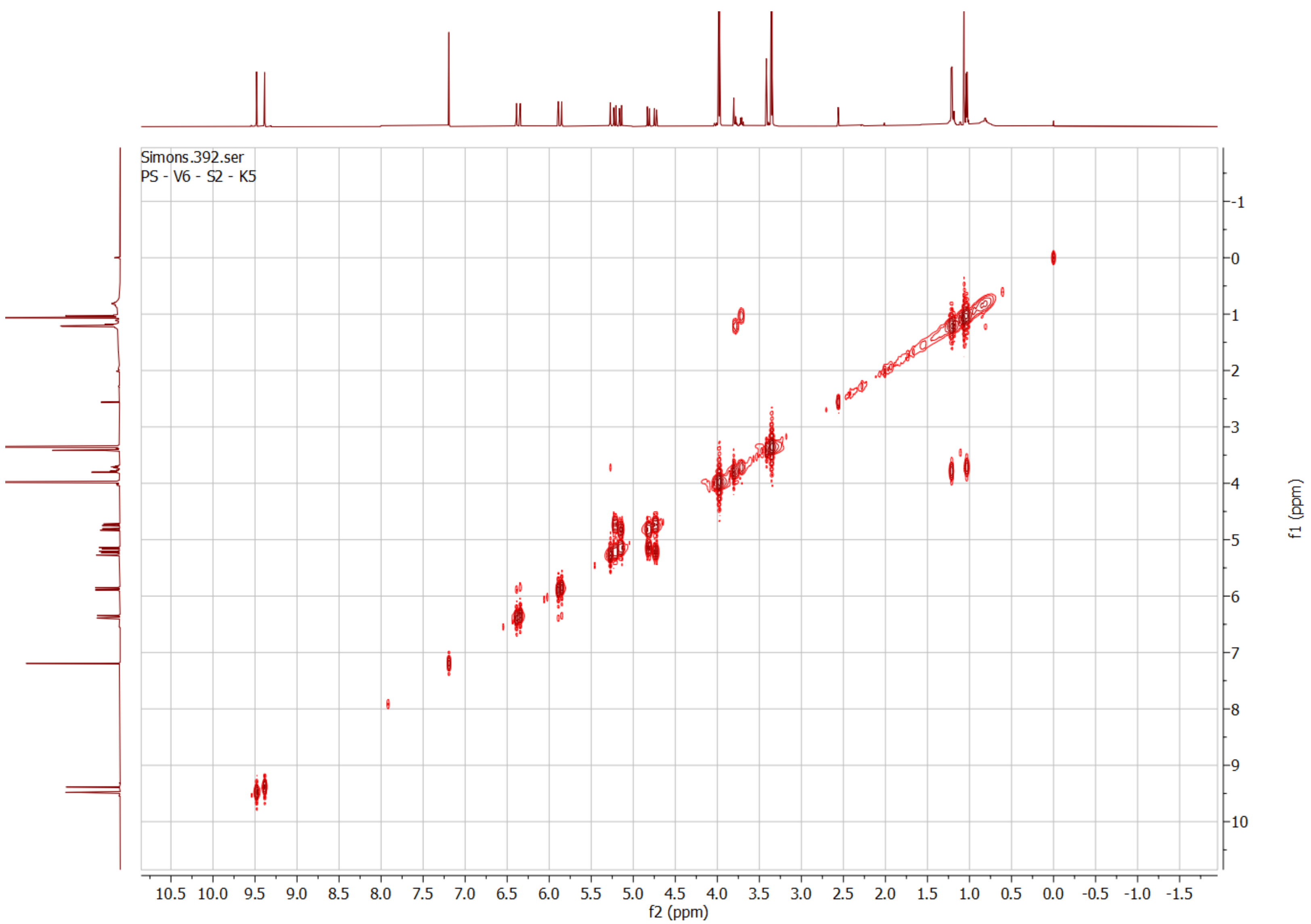

Figure **S21.** COSY NMR spectrum ($CDCl_3$ - 600 MHz) of compound E

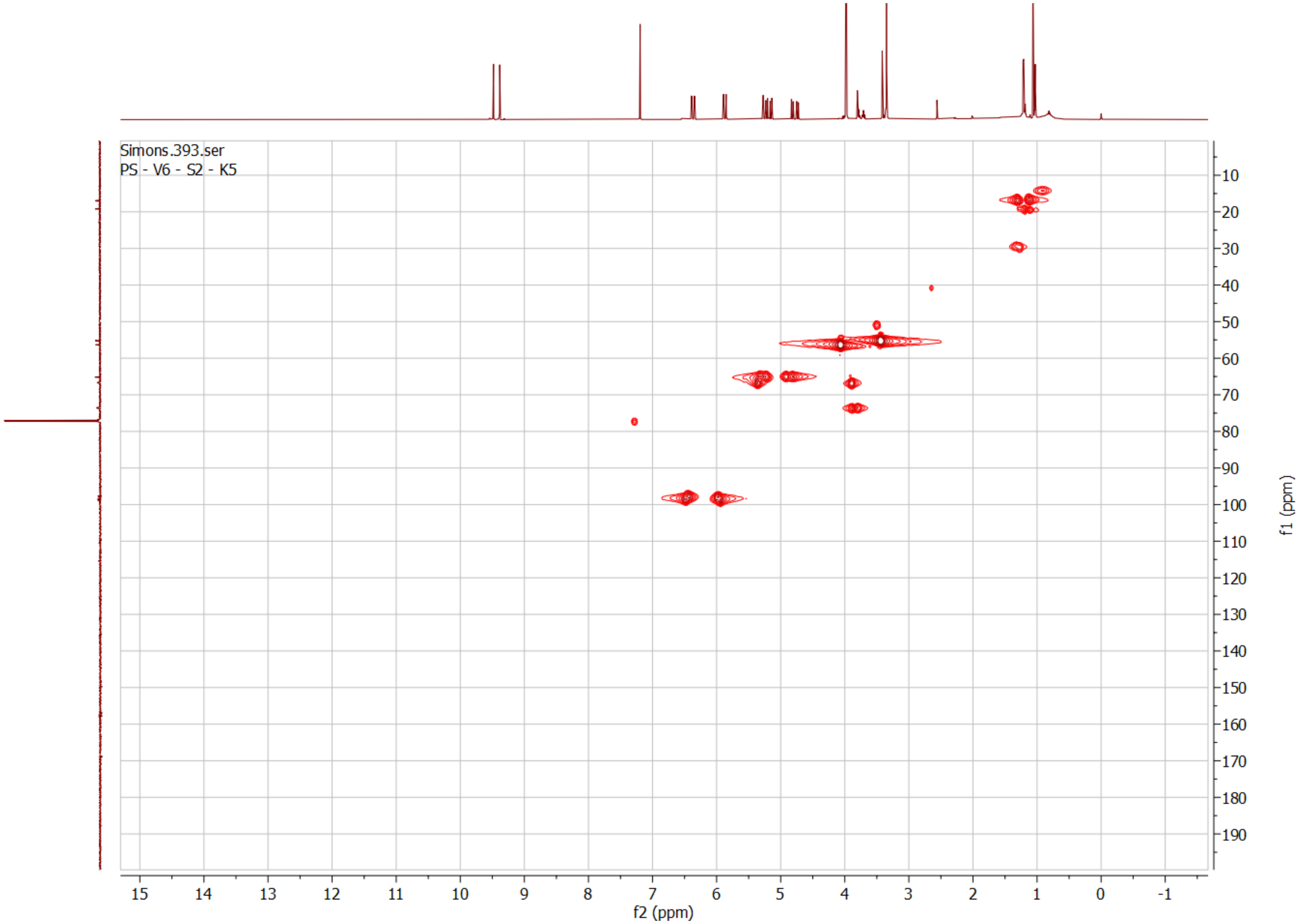

Figure **S22.** HSQC NMR spectrum ($CDCl_3$ - 600 MHz) of compound E

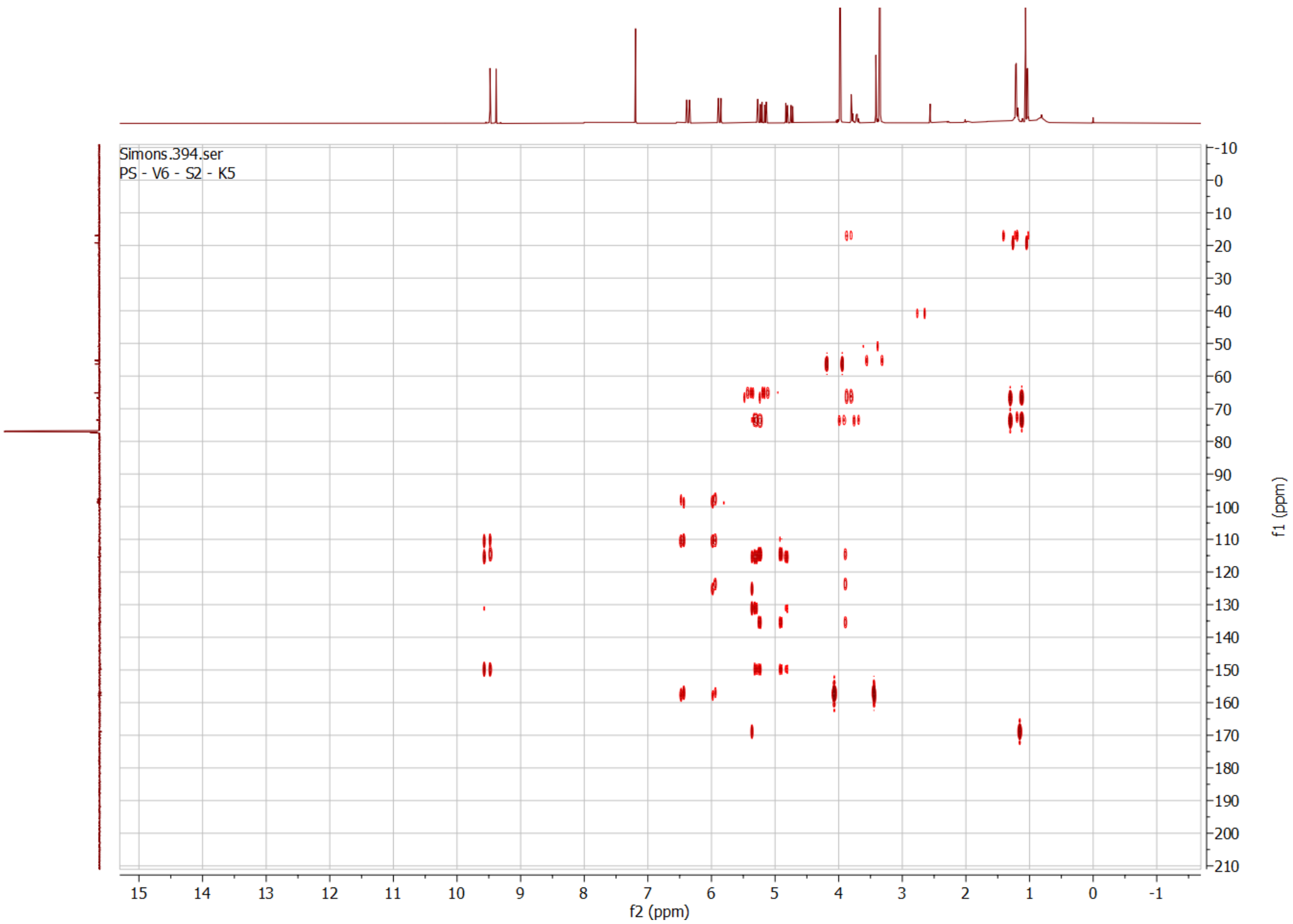

Figure **S23.** HMBC NMR spectrum ($CDCl_3$ - 600 MHz) of compound E

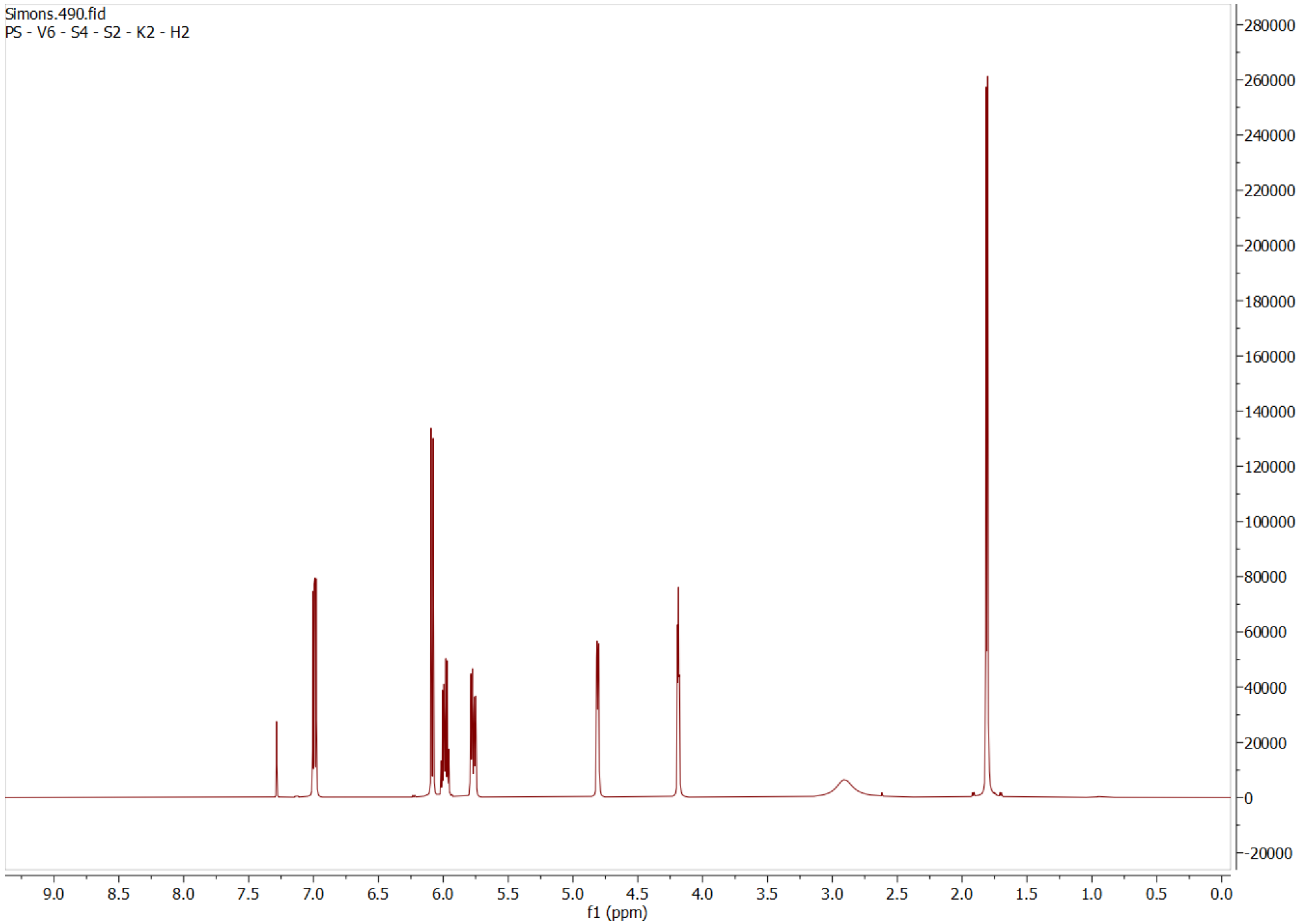

Figure **S24.** ^{1}H NMR spectrum ($CDCl_3$ - 600 MHz) of compound F

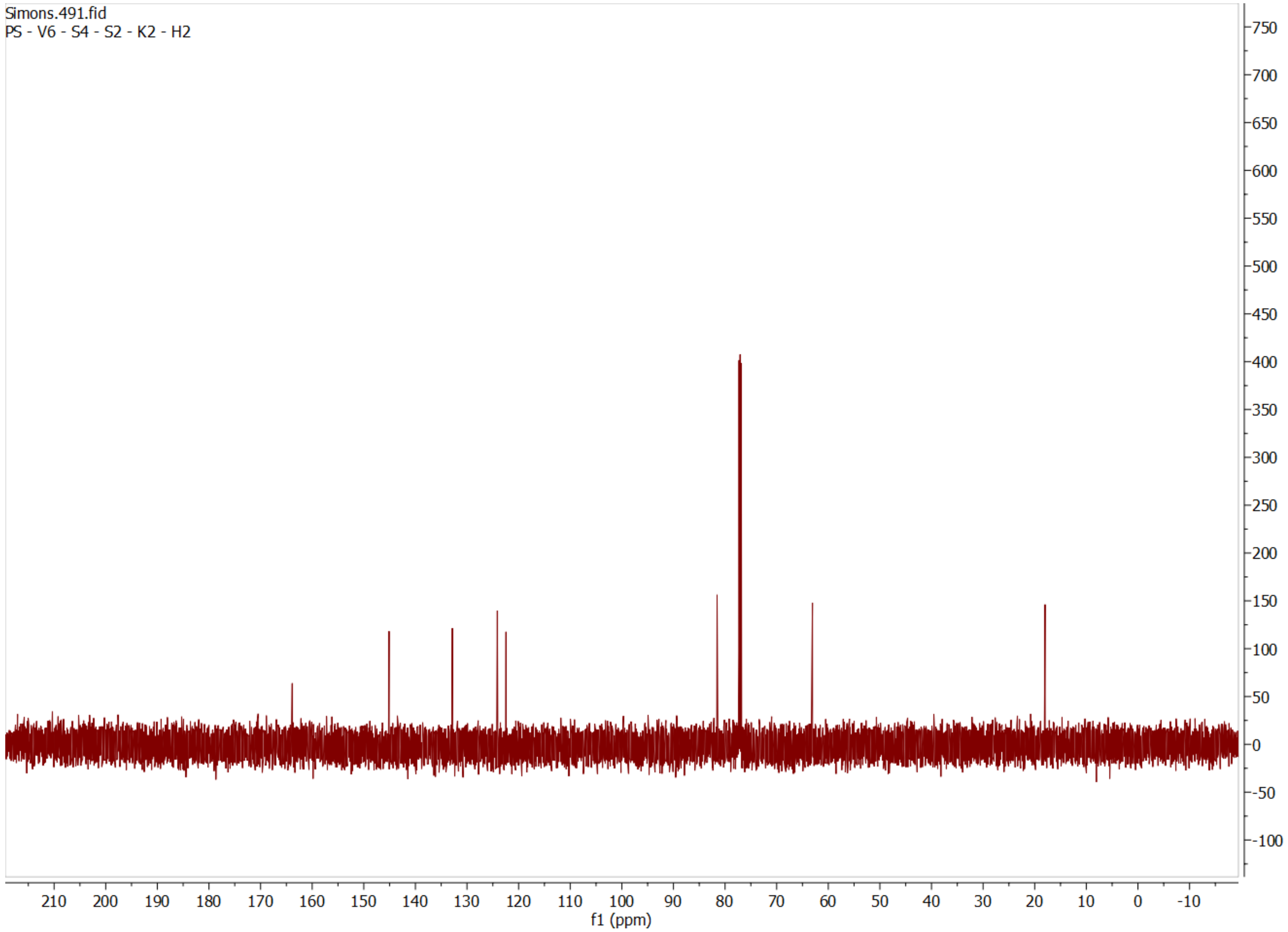

Figure **S25.** ^{13}C NMR spectrum ($CDCl_3$ - 150 MHz) of compound F

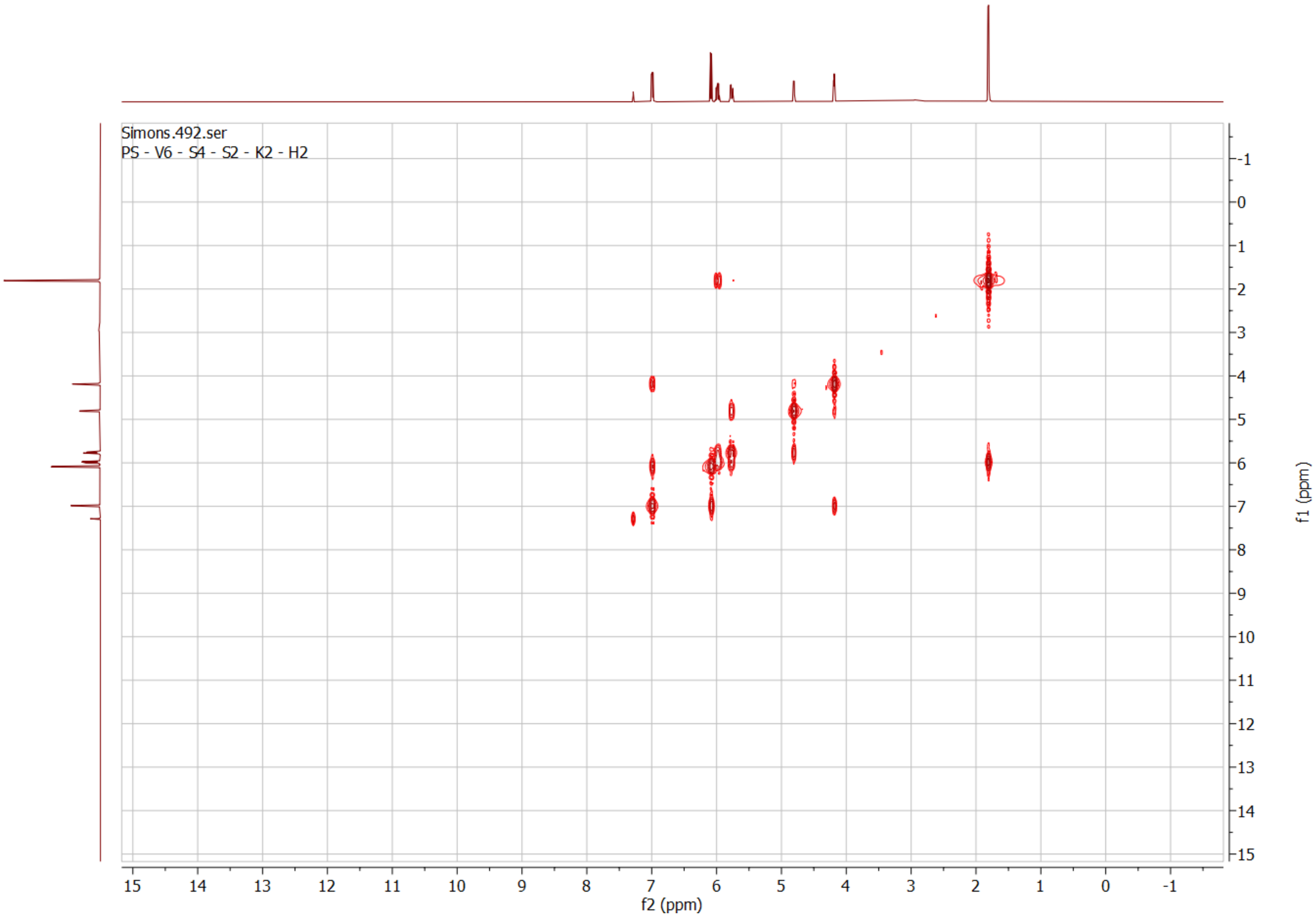

Figure **S26.** COSY NMR spectrum ($CDCl_3$ - 600 MHz) of compound F

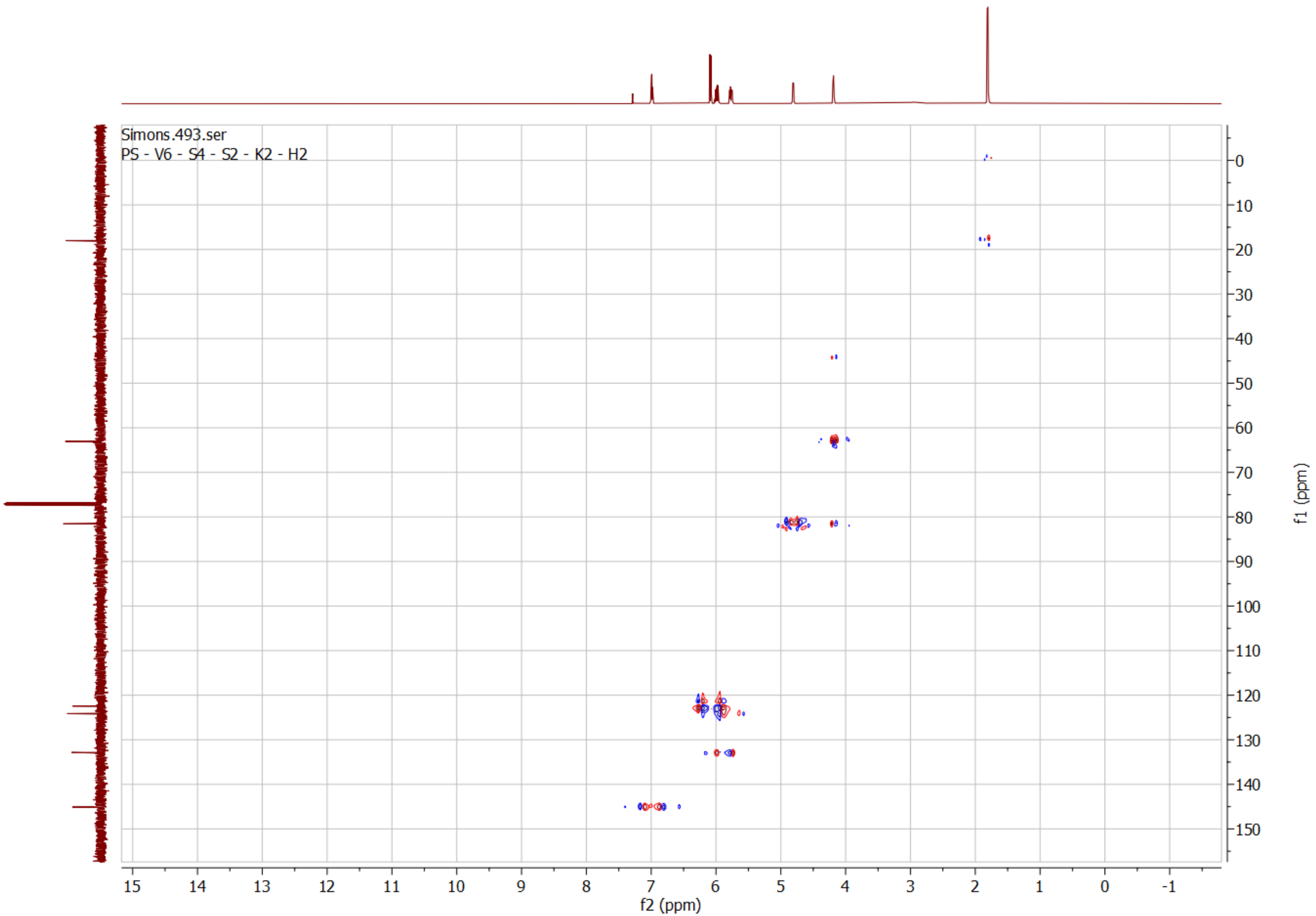

Figure **S27.** HSQC NMR spectrum (CDCl3 - 600 MHz) of compound F

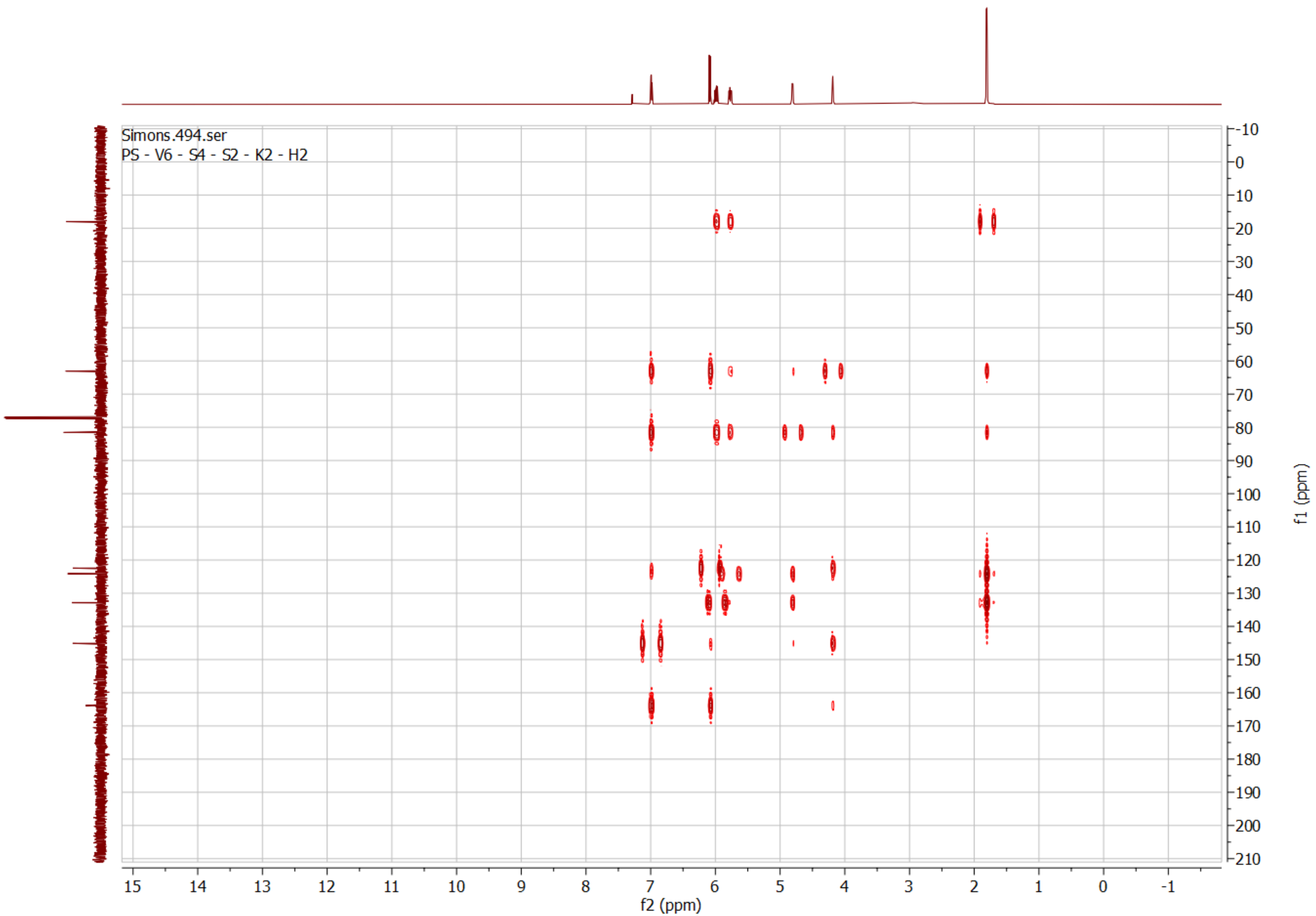

Figure **S28.** HMBC NMR spectrum ($CDCl_3$ - 600 MHz) of compound F

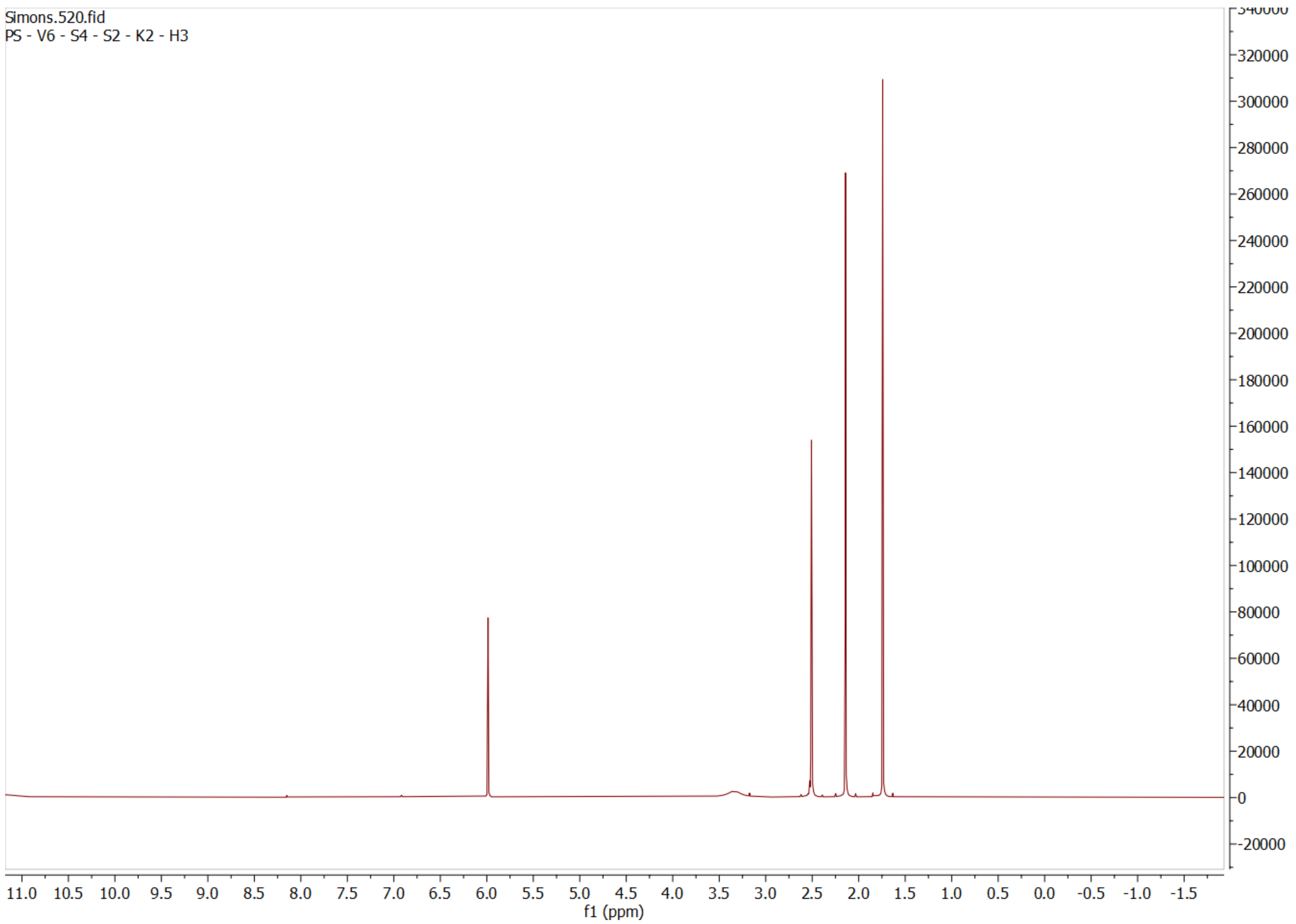

Figure **S29.** ^{1}H NMR spectrum (DMSO-d6 – 600 MHz) of compound G

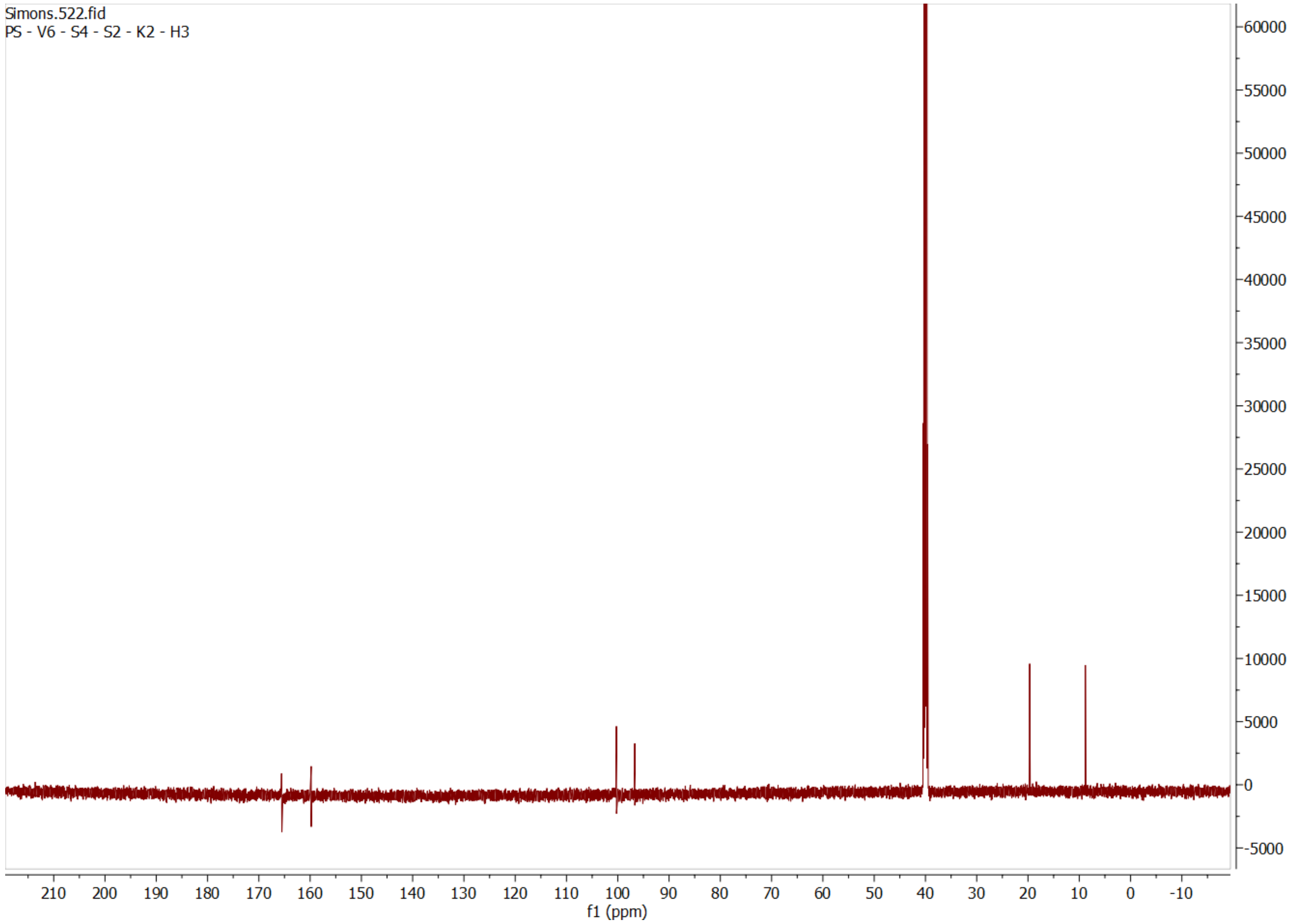

Figure **S30.** ^{13}C NMR spectrum (DMSO-d6 – 150 MHz) of compound G

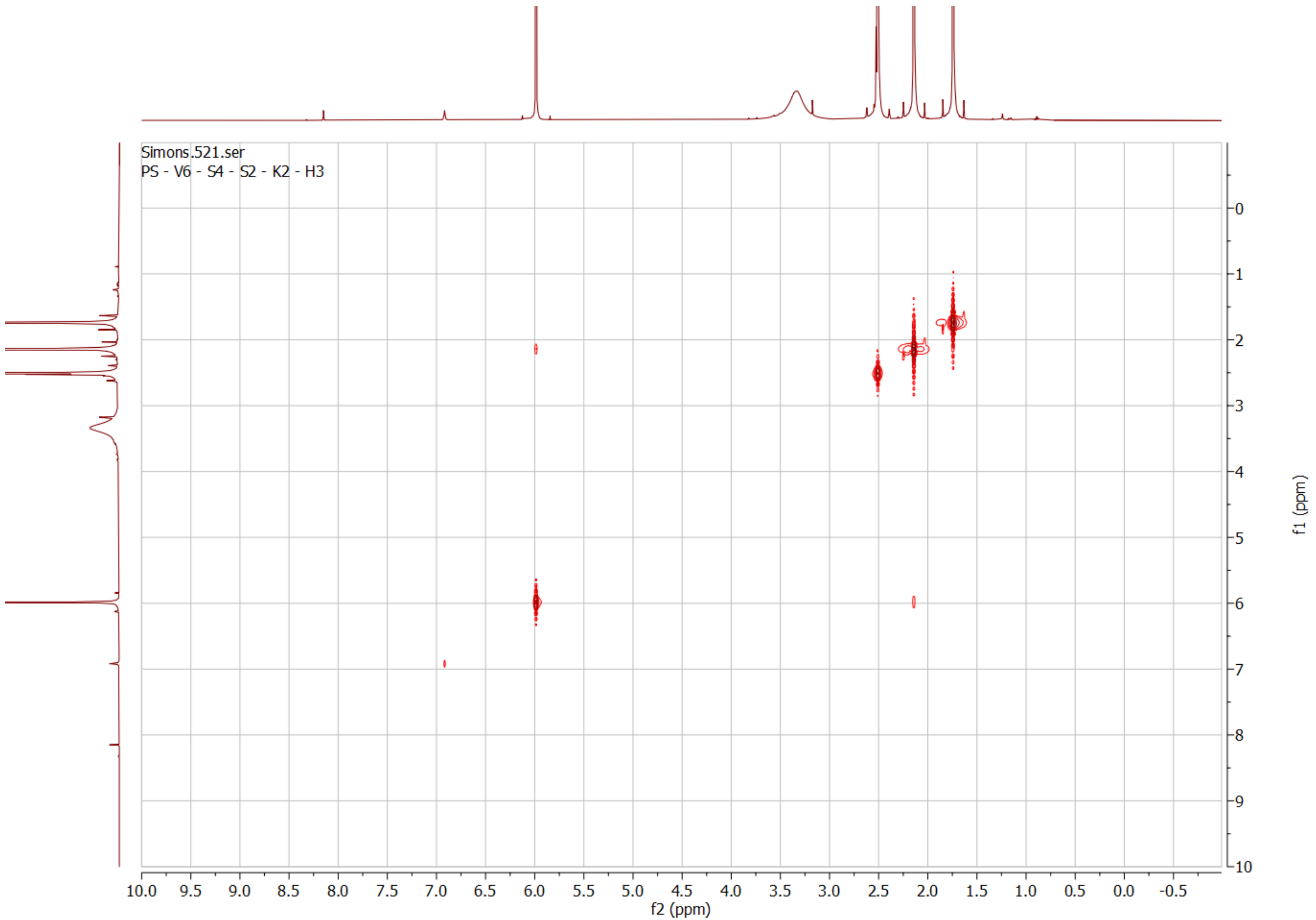

Figure **S31.** COSY NMR spectrum (DMSO-d6 – 600 MHz) of compound G

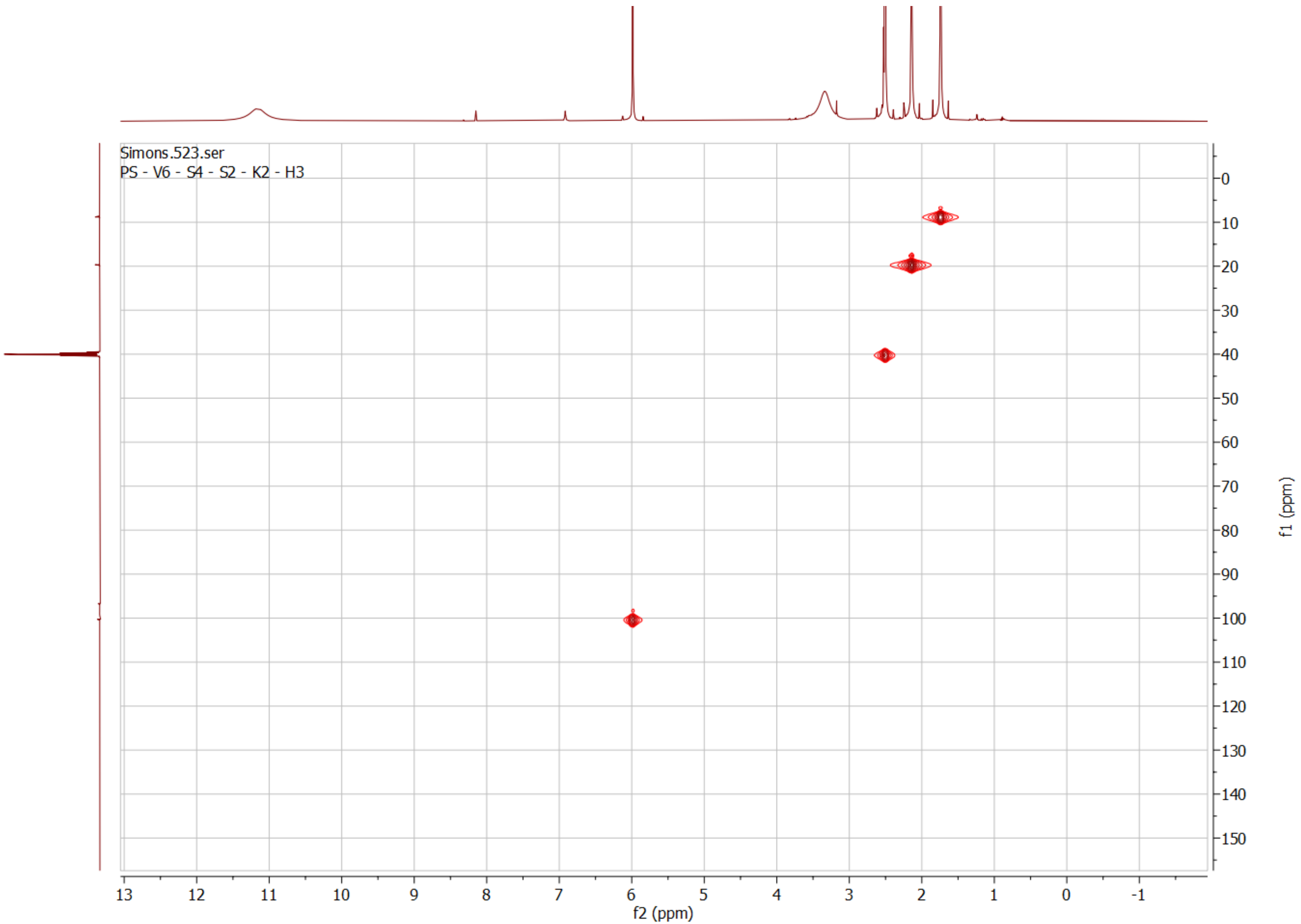

Figure **S32.** HSQC NMR spectrum (DMSO-d6 – 600 MHz) of compound G

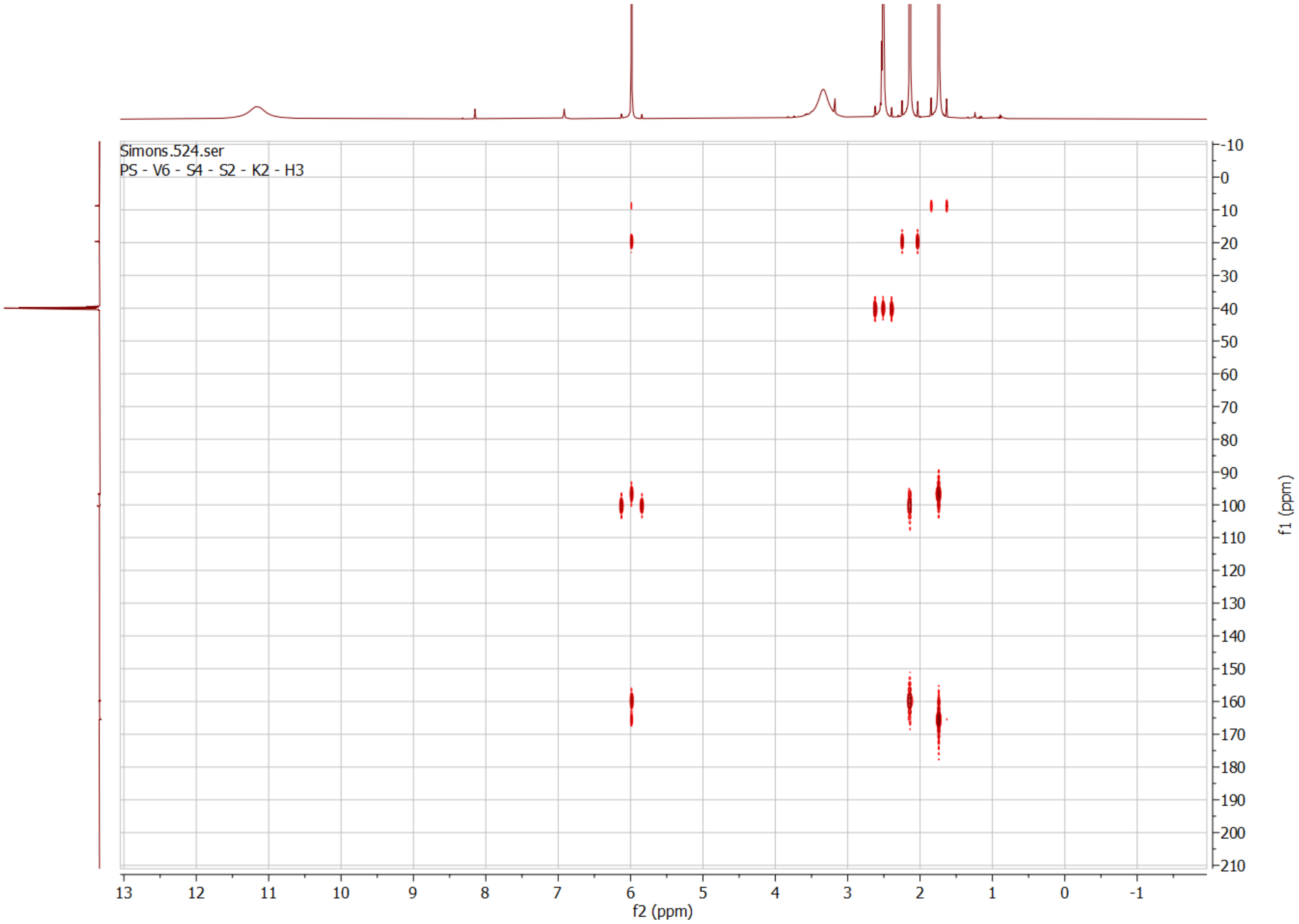

Figure **S33.** HMBC NMR spectrum (DMSO-d6 – 600 MHz) of compound G

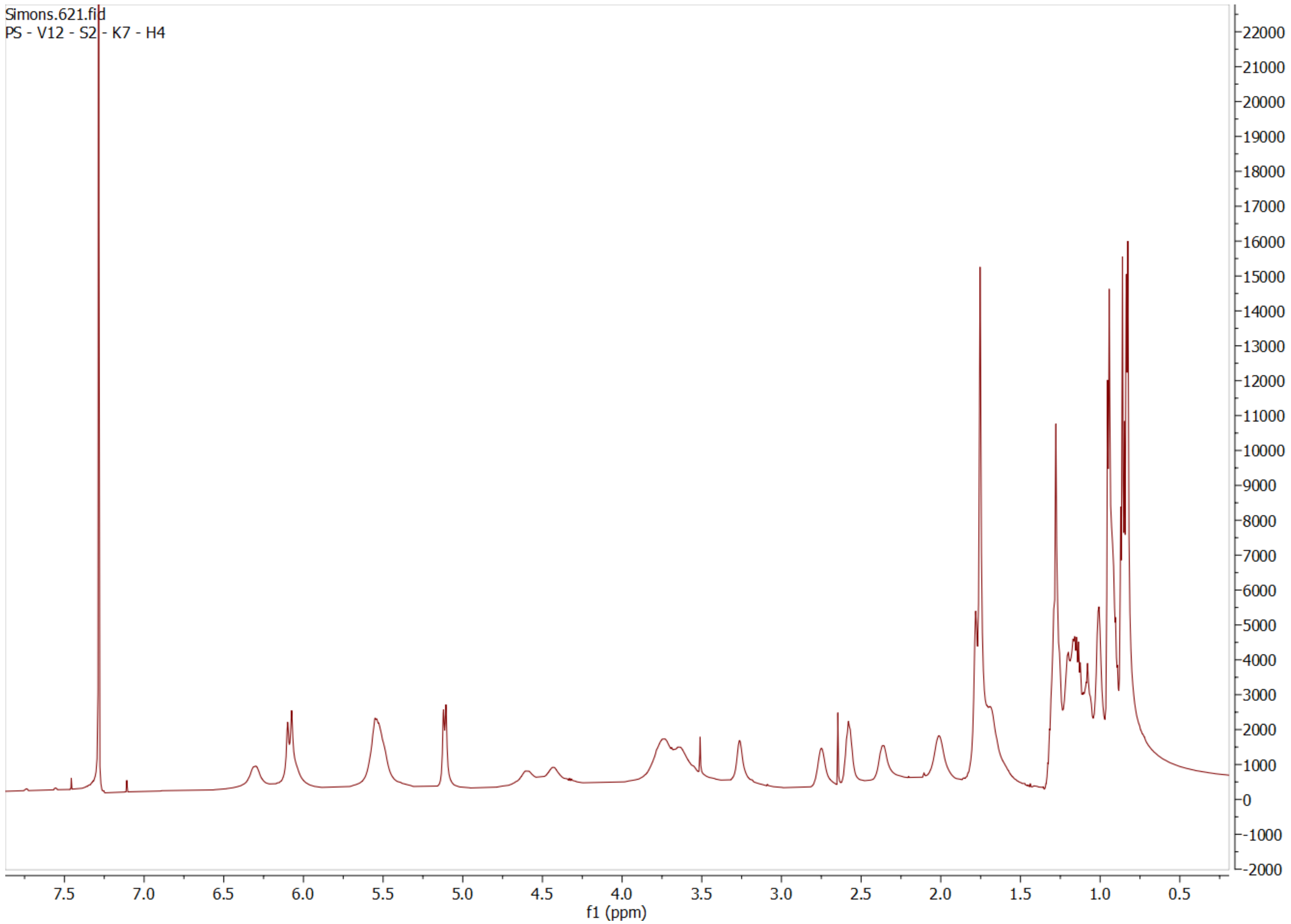

Figure **S34.** ^{1}H NMR spectrum ($CDCl_3$ - 600 MHz) of compound H

Acquisition Parameter

Source Type	ESI	Ion Polarity	Positive	Set Nebulizer	0.3 Bar
Focus	Not active	Set Capillary	4000 V	Set Dry Heater	180 °C
Scan Begin	50 m/z	Set End Plate Offset	-500 V	Set Dry Gas	4.0 l/min
Scan End	1500 m/z	Set Collision Cell RF	600.0 Vpp	Set Divert Valve	Source

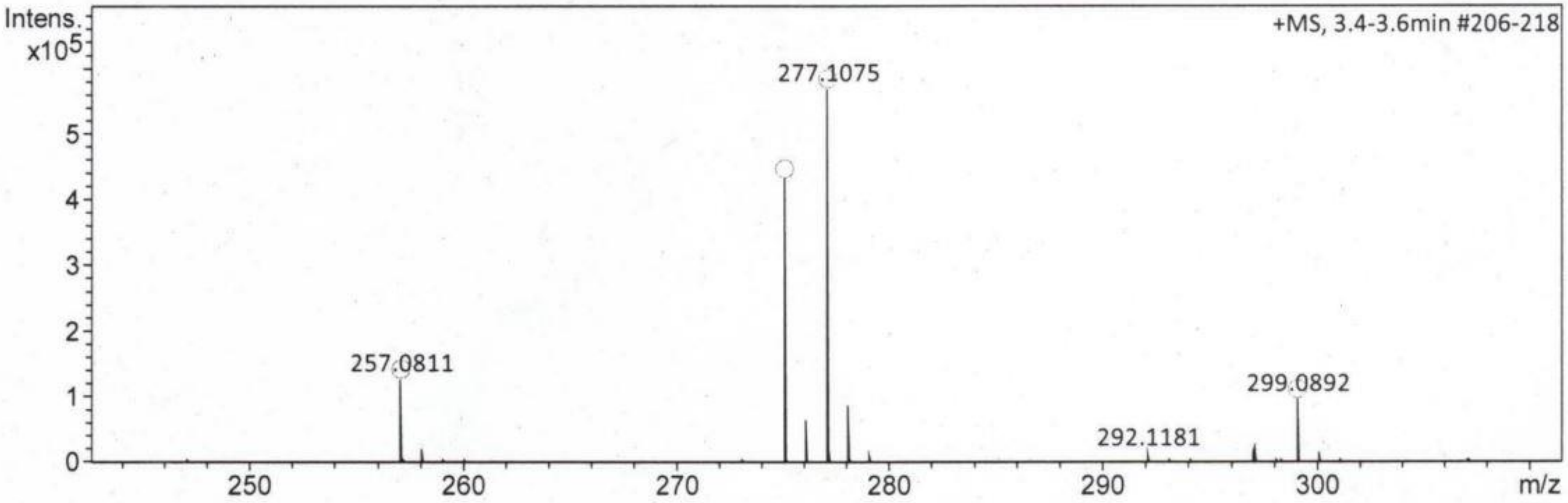

Meas. m/z	#	Ion Formula	m/z	err [ppm]	mSigma	# mSigma	Score	rdb	e⁻ Conf	N-Rule
257.0811	1	C15H13O4	257.0808	-1.1	7.2	1	100.00	9.5	even	ok
275.0919	1	C15H15O5	275.0914	-1.8	581.8	1	100.00	8.5	even	ok
277.1075	1	C15H17O5	277.1071	-1.6	8.4	1	100.00	7.5	even	ok
299.0892	1	C15H16NaO5	299.0890	-0.8	0.6	1	100.00	7.5	even	ok

Figure **S35.** HRESIMS (MeOH) of compound A

Acquisition Parameter

Source Type	ESI	Ion Polarity	Positive	Set Nebulizer	0.3 Bar		
Focus	Not active	Set Capillary	4000 V	Set Dry Heater	180 °C		
Scan Begin	50 m/z	Set End Plate Offset	-500 V	Set Dry Gas	4.0 l/min		
Scan End	1500 m/z	Set Collision Cell RF	600.0 Vpp	Set Divert Valve	Source		

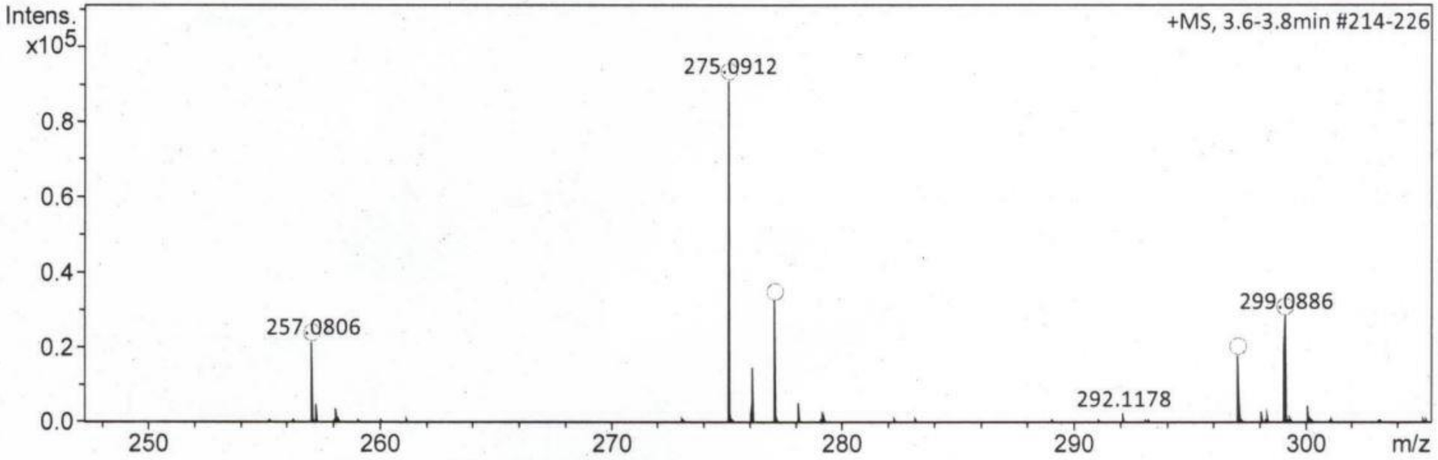

Meas. m/z	#	Ion Formula	m/z	err [ppm]	mSigma	# mSigma	Score	rdb	e⁻ Conf	N-Rule
257.0806	1	C15H13O4	257.0808	1.1	5.7	1	100.00	9.5	even	ok
275.0912	1	C15H15O5	275.0914	0.7	192.5	1	100.00	8.5	even	ok
277.1065	1	C15H17O5	277.1071	2.1	4.8	1	98.95	7.5	even	ok
	2	C14H14N4NaO	277.1060	-1.8	6.5	2	100.00	9.5	even	ok
297.0730	1	C13H9N6O3	297.0731	0.3	11.1	1	100.00	12.5	even	ok
	2	C15H14NaO5	297.0733	1.2	602.8	2	0.00	8.5	even	ok
299.0886	1	C13H11N6O3	299.0887	0.4	3.4	1	100.00	11.5	even	ok
	2	C15H16NaO5	299.0890	1.3	4.2	2	87.30	7.5	even	ok

Figure **S36.** HRESIMS (MeOH) of compound B

Acquisition Parameter

Source Type	ESI	Ion Polarity	Positive	Set Nebulizer	0.3 Bar
Focus	Not active	Set Capillary	4000 V	Set Dry Heater	180 °C
Scan Begin	50 m/z	Set End Plate Offset	-500 V	Set Dry Gas	4.0 l/min
Scan End	1500 m/z	Set Collision Cell RF	600.0 Vpp	Set Divert Valve	Source

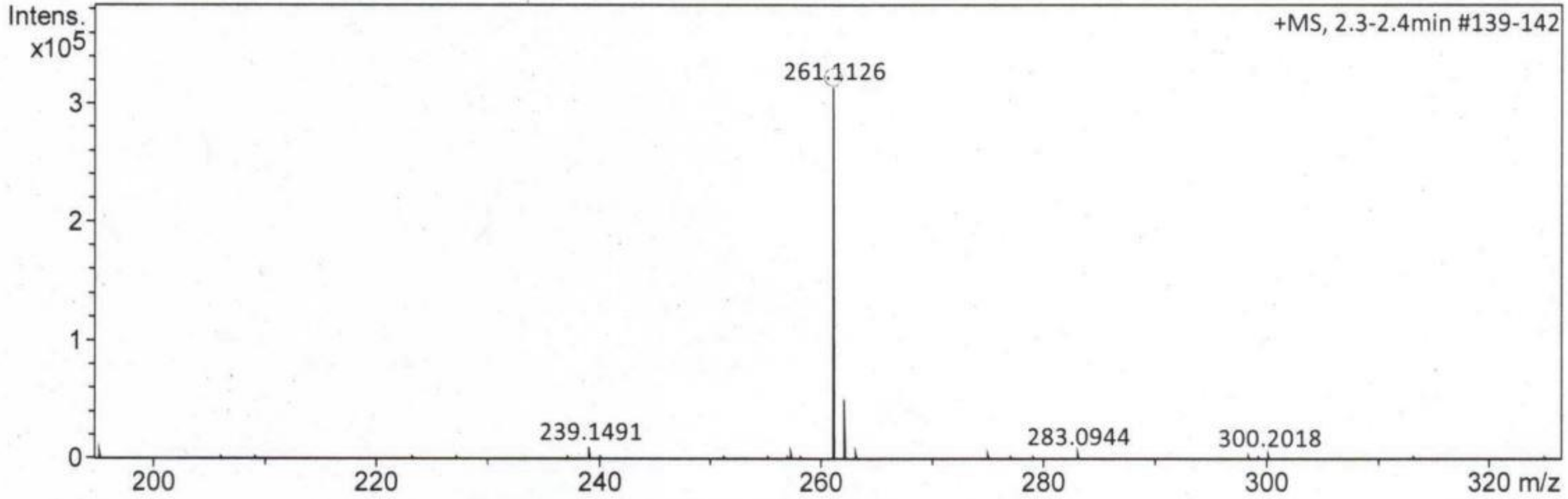

Meas. m/z	#	Ion Formula	m/z	err [ppm]	mSigma	# mSigma	Score	rdb	e⁻ Conf	N-Rule
261.1126	1	$C_{15}H_{17}O_4$	261.1121	-1.8	2.6	1	100.00	7.5	even	ok

Figure **S37.** HRESIMS (MeOH) of compound C

Acquisition Parameter

Source Type	ESI	Ion Polarity	Positive	Set Nebulizer	0.3 Bar
Focus	Not active	Set Capillary	4000 V	Set Dry Heater	180 °C
Scan Begin	50 m/z	Set End Plate Offset	-500 V	Set Dry Gas	4.0 l/min
Scan End	1500 m/z	Set Collision Cell RF	600.0 Vpp	Set Divert Valve	Source

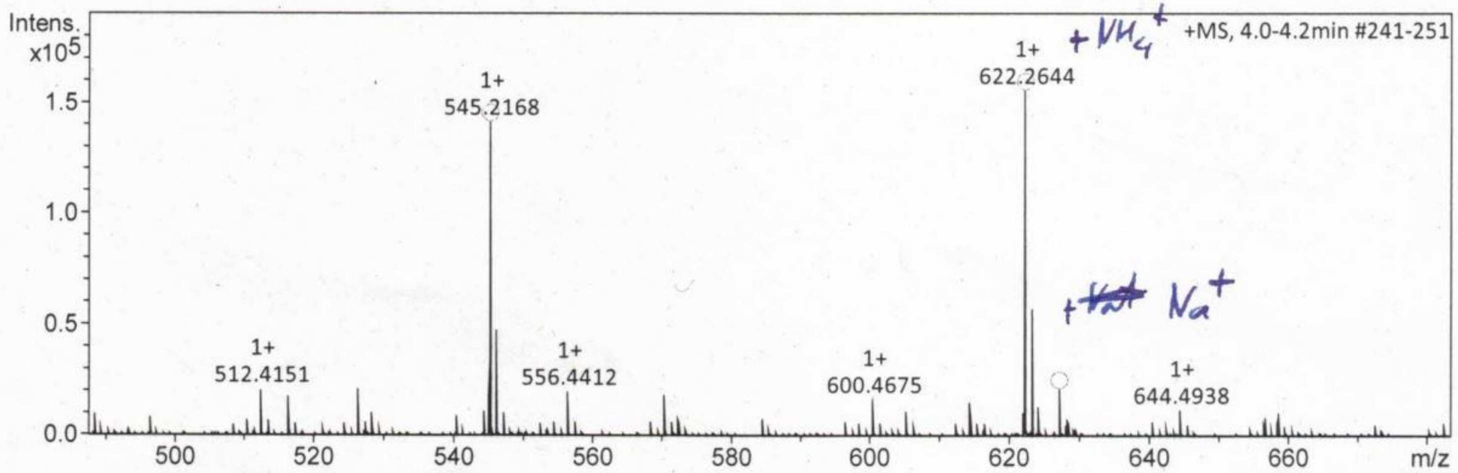

Meas. m/z	#	Ion Formula	m/z	err [ppm]	mSigma	# mSigma	Score	rdb	e⁻ Conf	N-Rule
545.2168	1	C32H33O8	545.2170	0.4	8.6	1	100.00	16.5	even	ok
	2	C29H25N10O2	545.2156	-2.1	10.0	2	60.03	22.5	even	ok
	3	C33H29N4O4	545.2183	2.8	20.2	3	38.17	21.5	even	ok
622.2644	1	C34H40NO10	622.2647	0.4	6.3	1	100.00	15.5	even	ok
	2	C31H32N11O4	622.2633	-1.8	7.7	2	63.41	21.5	even	ok
	3	C35H36N5O6	622.2660	2.6	17.9	3	38.03	20.5	even	ok
	4	C32H28N15	622.2647	0.4	18.6	4	79.33	26.5	even	ok
627.2193	1	C34H36NaO10	627.2201	1.2	11.0	1	100.00	16.5	even	ok
	2	C30H32N6NaO8	627.2174	-3.1	11.3	2	45.90	17.5	even	ok
	3	C31H28N10NaO4	627.2187	-1.0	14.8	3	98.78	22.5	even	ok
	4	C32H24N14Na	627.2201	1.2	24.2	4	76.55	27.5	even	ok

Figure **S38.** HRESIMS (MeOH) of compound D

Acquisition Parameter

Source Type	ESI	Ion Polarity	Positive	Set Nebulizer	0.3 Bar
Focus	Not active	Set Capillary	4000 V	Set Dry Heater	180 °C
Scan Begin	50 m/z	Set End Plate Offset	-500 V	Set Dry Gas	4.0 l/min
Scan End	1500 m/z	Set Collision Cell RF	600.0 Vpp	Set Divert Valve	Source

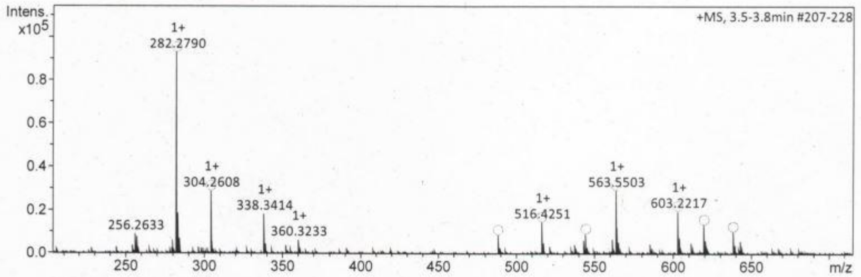

Meas. m/z	#	Ion Formula	m/z	err [ppm]	mSigma	# mSigma	Score	rdb	e⁻ Conf	N-Rule
282.2790	1	C18H36NO	282.2791	0.4	30.2	1	100.00	1.5	even	ok
304.2608	1	C18H35NNaO	304.2611	0.9	13.8	1	100.00	1.5	even	ok
	2	C15H34N3O3	304.2595	-4.5	20.4	2	48.40	0.5	even	ok
488.3939	1	C23H50N7O4	488.3919	-4.1	27.1	1	34.39	2.5	even	ok
	2	C24H46N11	488.3932	-1.4	27.8	2	79.11	7.5	even	ok
	3	C27H54NO6	488.3946	1.4	29.9	3	100.00	1.5	even	ok
	4	C26H51N5NaO2	488.3935	-0.8	30.4	4	85.24	3.5	even	ok
	5	C28H50N5O2	488.3959	4.1	39.6	5	24.93	6.5	even	ok
516.4251	1	C29H58NO6	516.4259	1.5	9.1	1	100.00	1.5	even	ok
	2	C28H55N5NaO2	516.4248	-0.6	11.1	2	90.44	3.5	even	ok
	3	C26H50N11	516.4245	-1.1	13.2	3	76.15	7.5	even	ok
	4	C25H54N7O4	516.4232	-3.7	24.7	4	26.14	2.5	even	ok
544.4564	1	C31H62NO6	544.4572	1.4	11.0	1	83.99	1.5	even	ok
	2	C27H58N7O4	544.4545	-3.5	13.4	2	39.01	2.5	even	ok
	3	C30H59N5NaO2	544.4561	-0.5	14.2	3	100.00	3.5	even	ok
	4	C28H54N11	544.4558	-1.1	14.5	4	65.37	7.5	even	ok
563.5503	1	C36H71N2O2	563.5510	1.3	54.5	1	100.00	2.5	even	ok
	2	C34H72N2NaO2	563.5486	-3.0	61.2	2	44.45	-0.5	even	ok
	3	C32H67N8	563.5483	-3.5	63.6	3	33.38	3.5	even	ok
603.2217	1	C34H35O10	603.2225	1.3	4.9	1	83.81	17.5	even	ok
	2	C33H32N4NaO6	603.2214	-0.5	7.7	2	100.00	19.5	even	ok
	3	C31H27N10O4	603.2211	-1.0	10.0	3	83.93	23.5	even	ok
	4	C34H28N8NaO2	603.2227	1.7	11.6	4	64.05	24.5	even	ok
	5	C32H23N14	603.2225	1.2	12.8	5	73.02	28.5	even	ok
	6	C30H24N14Na	603.2201	-2.7	13.7	6	41.54	25.5	even	ok
	7	C32H36NaO10	603.2201	-2.7	16.0	7	39.93	14.5	even	ok
	8	C30H31N6O8	603.2198	-3.2	17.6	8	31.61	18.5	even	ok
619.6126	1	C40H79N2O2	619.6136	1.6	28.2	1	100.00	2.5	even	ok
	2	C38H80N2NaO2	619.6112	-2.3	35.7	2	64.89	-0.5	even	ok
	3	C36H75N8	619.6109	-2.7	38.1	3	50.45	3.5	even	ok
638.2588	1	C32H41NNaO11	638.2572	-2.5	5.5	1	56.61	12.5	even	ok
	2	C30H36N7O9	638.2569	-3.0	7.4	2	44.83	16.5	even	ok
	3	C34H40NO11	638.2596	1.2	13.7	3	81.16	15.5	even	ok
	4	C33H37N5NaO7	638.2585	-0.4	15.4	4	100.00	17.5	even	ok
	5	C31H32N11O5	638.2582	-0.9	15.8	5	87.00	21.5	even	ok
	6	C30H29N15NaO	638.2572	-2.6	18.7	6	43.63	23.5	even	ok
	7	C32H28N15O	638.2596	1.2	26.8	7	61.67	26.5	even	ok
	8	C34H33N9NaO3	638.2599	1.6	26.8	8	52.80	22.5	even	ok
	9	C47H32N3	638.2591	0.4	87.5	9	10.66	33.5	even	ok

Figure **S39.** HRESIMS (MeOH) of compound E

Acquisition Parameter

Source Type	ESI	Ion Polarity	Positive	Set Nebulizer	0.3 Bar
Focus	Not active	Set Capillary	4000 V	Set Dry Heater	180 °C
Scan Begin	50 m/z	Set End Plate Offset	-500 V	Set Dry Gas	4.0 l/min
Scan End	1500 m/z	Set Collision Cell RF	600.0 Vpp	Set Divert Valve	Source

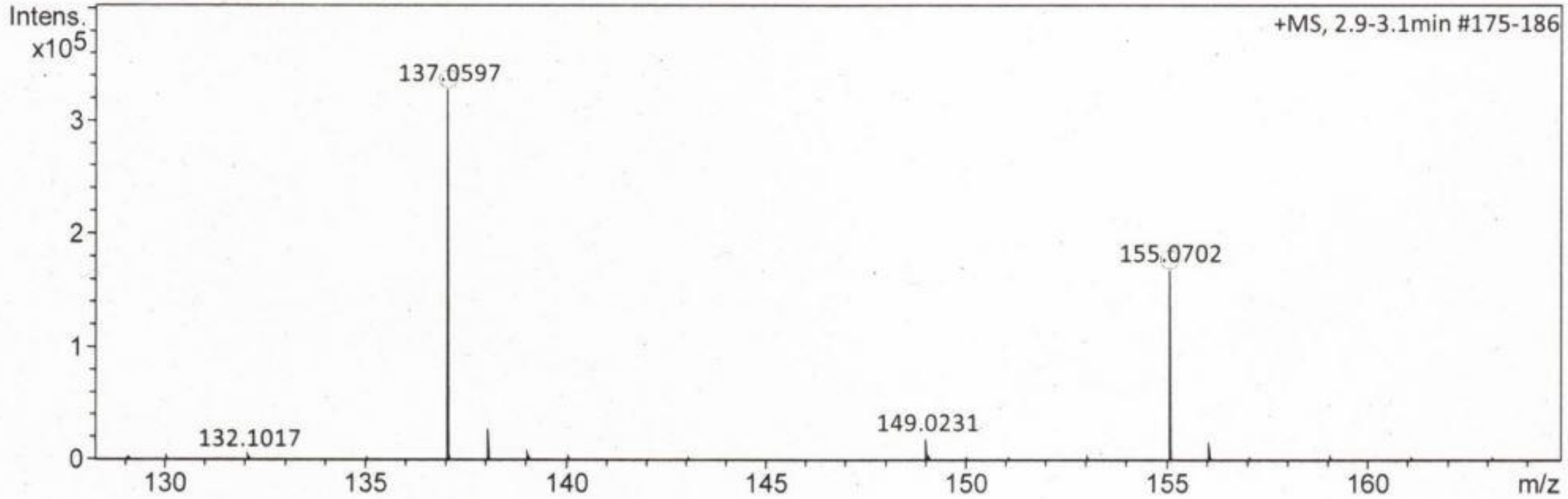

Meas. m/z	#	Ion Formula	m/z	err [ppm]	mSigma	# mSigma	Score	rdb	e⁻ Conf	N-Rule
137.0597	1	C8H9O2	137.0597	0.3	2.4	1	100.00	4.5	even	ok
155.0702	1	C8H11O3	155.0703	0.6	3.0	1	100.00	3.5	even	ok

Figure **S40.** HRESIMS (MeOH) of compound F

Acquisition Parameter

Source Type	ESI	Ion Polarity	Positive	Set Nebulizer	0.3 Bar
Focus	Not active	Set Capillary	4000 V	Set Dry Heater	180 °C
Scan Begin	50 m/z	Set End Plate Offset	-500 V	Set Dry Gas	4.0 l/min
Scan End	1500 m/z	Set Collision Cell RF	600.0 Vpp	Set Divert Valve	Source

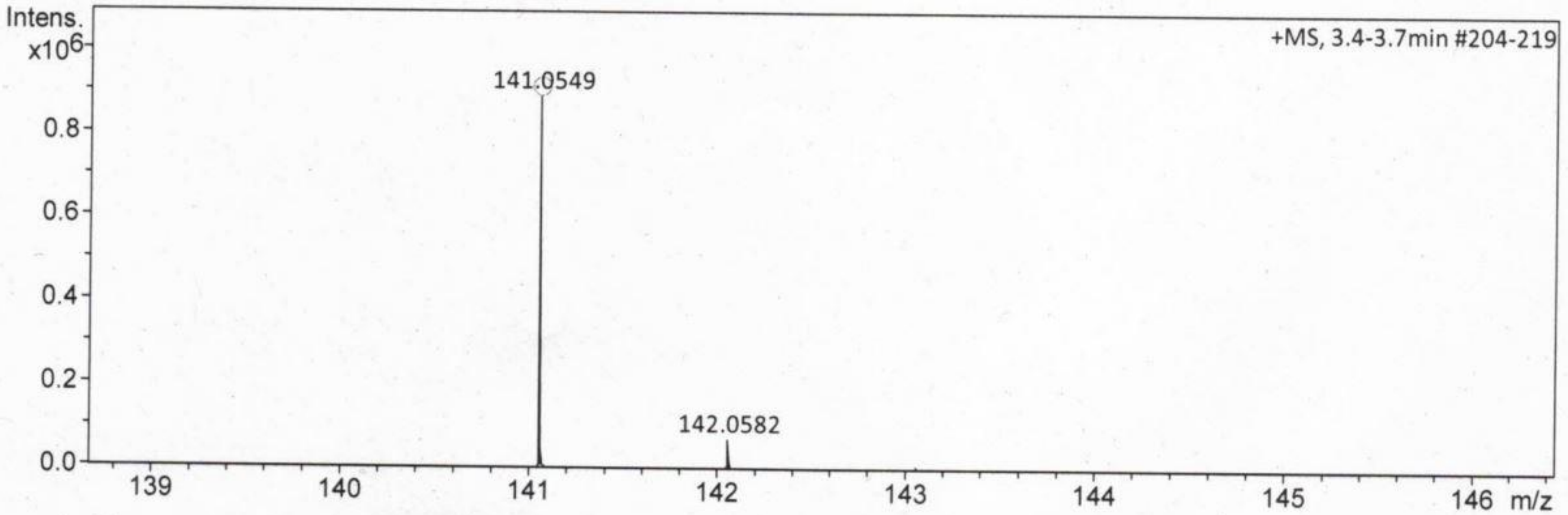

Meas. m/z	#	Ion Formula	m/z	err [ppm]	mSigma	# mSigma	Score	rdb	e¯ Conf	N-Rule
141.0549	1	C7H9O3	141.0546	-1.7	1.3	1	100.00	3.5	even	ok

Figure **S41.** HRESIMS (MeOH) of compound G

Acquisition Parameter

Source Type	ESI	Ion Polarity	Positive	Set Nebulizer	0.3 Bar		
Focus	Not active	Set Capillary	4000 V	Set Dry Heater	180 °C		
Scan Begin	50 m/z	Set End Plate Offset	-500 V	Set Dry Gas	4.0 l/min		
Scan End	1500 m/z	Set Collision Cell RF	600.0 Vpp	Set Divert Valve	Source		

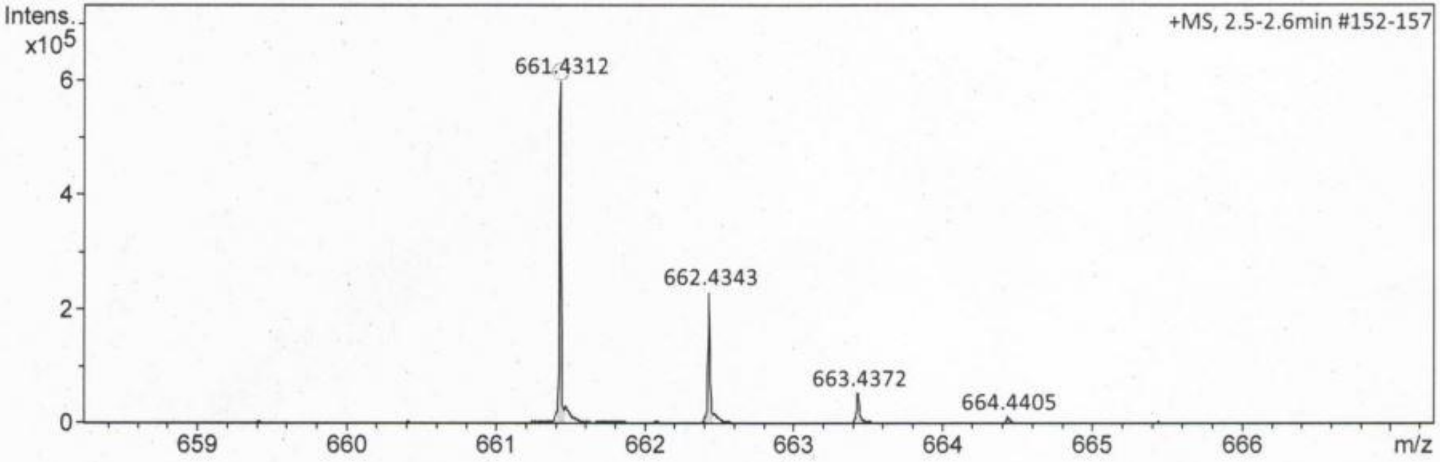

Meas. m/z	#	Ion Formula	m/z	err [ppm]	mSigma	# mSigma	Score	rdb	e⁻ Conf	N-Rule
661.4312	1	C38H61O9	661.4310	-0.3	20.0	1	100.00	8.5	even	ok
	2	C39H57N4O5	661.4323	1.7	31.3	2	48.27	13.5	even	ok

Figure **S42.** HRESIMS (MeOH) of compound H

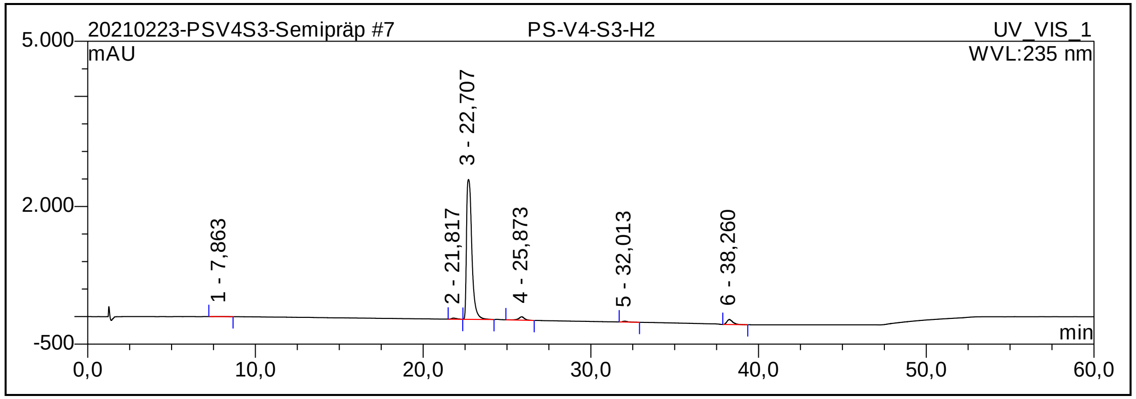

Figure **S43.** HPLC chromatogram (MeOH) of compound A

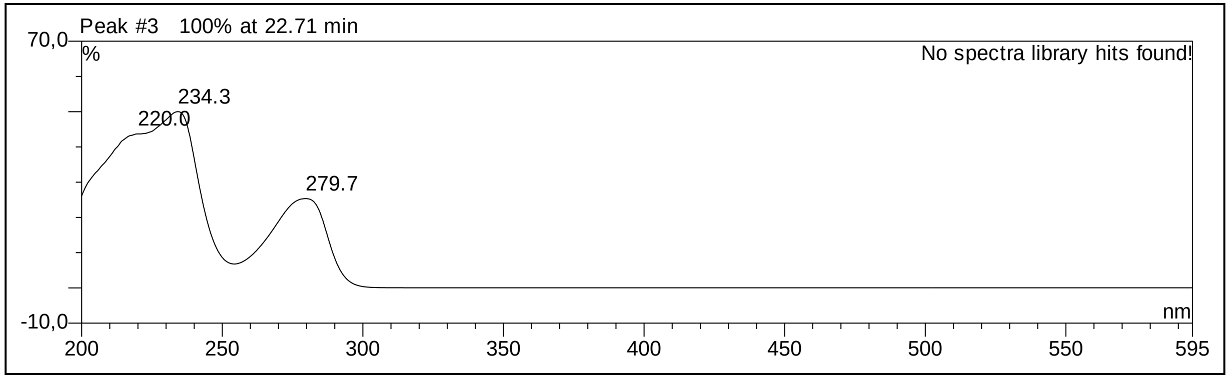

Figure **S44.** UV-Vis spectrum (MeOH) of compound A

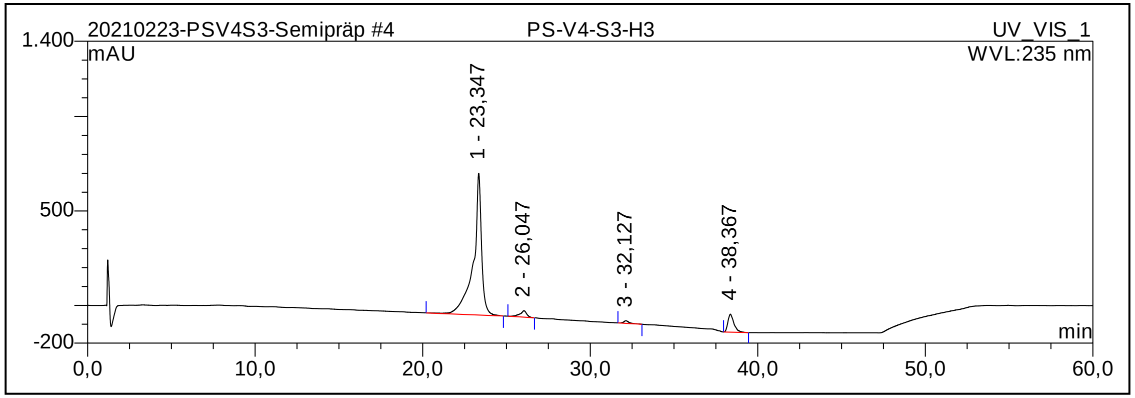

Figure **S45.** HPLC chromatogram (MeOH) of compound B

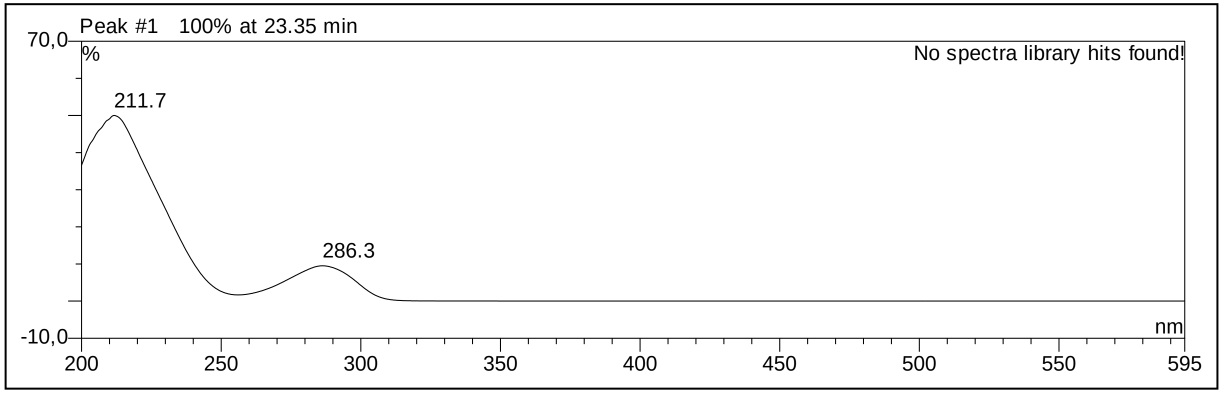

Figure **S46.** UV-Vis spectrum (MeoH) of compound B

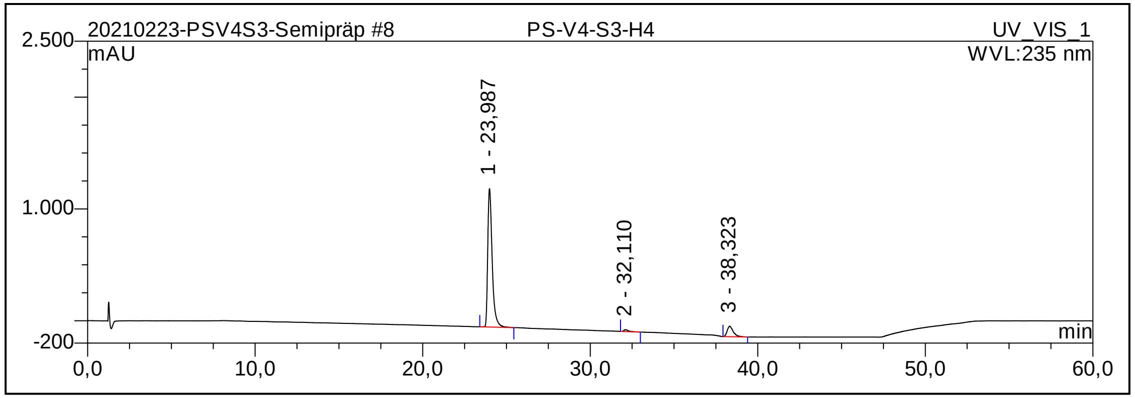

Figure **S47.** HPLC chromatogram (MeOH) of compound C

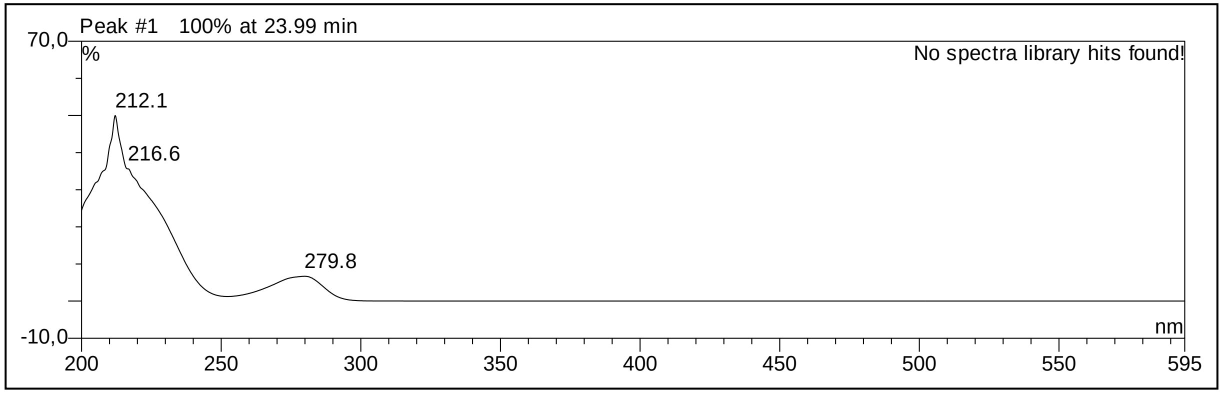

Figure **S48.** UV-Vis spectrum (MeOH) of compound C

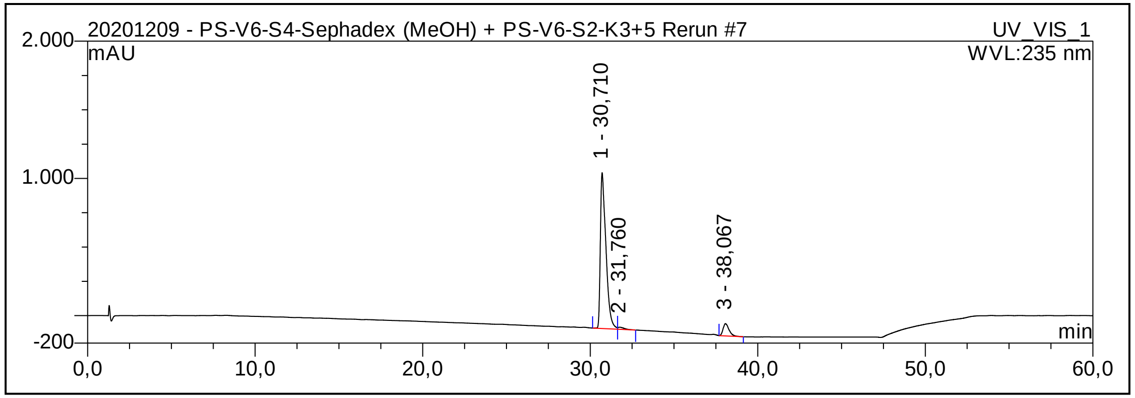

Figure **S49.** HPLC chromatogram (MeOH) of compound D

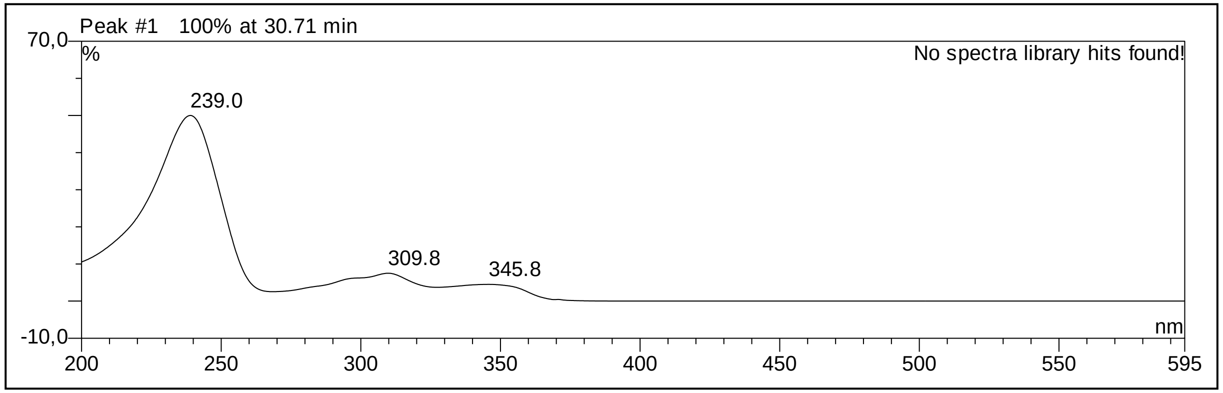

Figure **S50.** UV-Vis spectrum (MeOH) of compound D

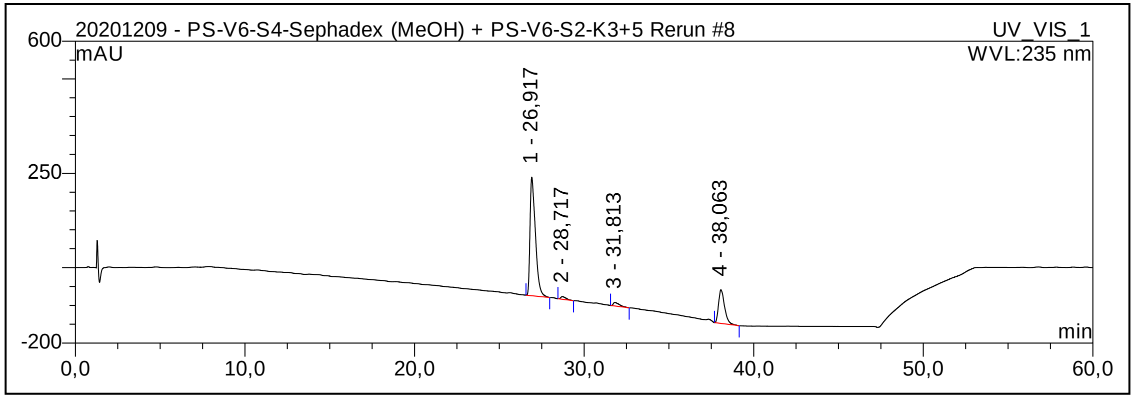

Figure **S51.** HPLC chromatogram (MeOH) of compound E

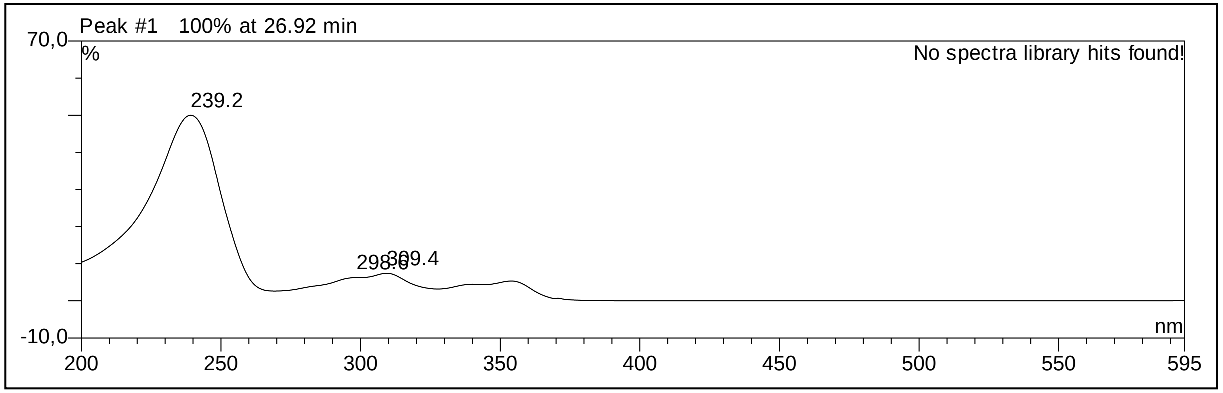

Figure **S52.** UV-Vis spectrum (MeOH) of compound E

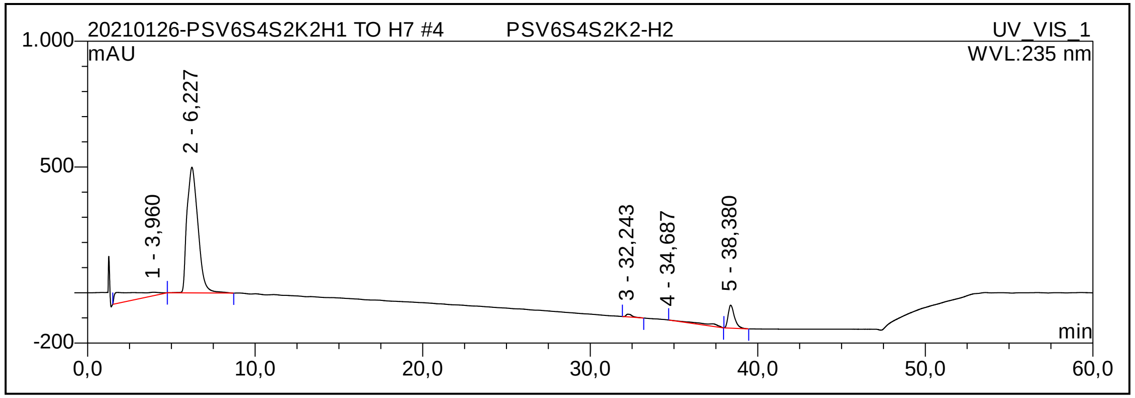

Figure **S53.** HPLC chromatogram (MeOH) of compound F

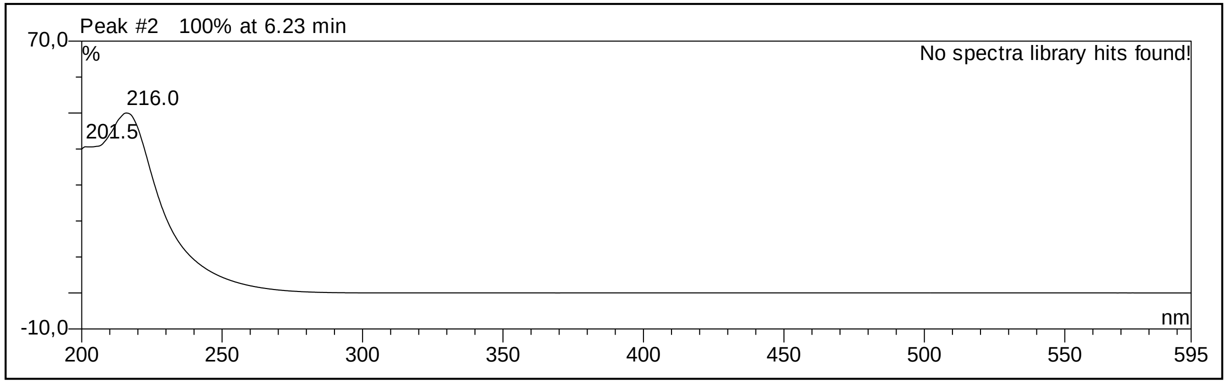

Figure **S54.** UV-Vis spectrum (MeOH) of compound F

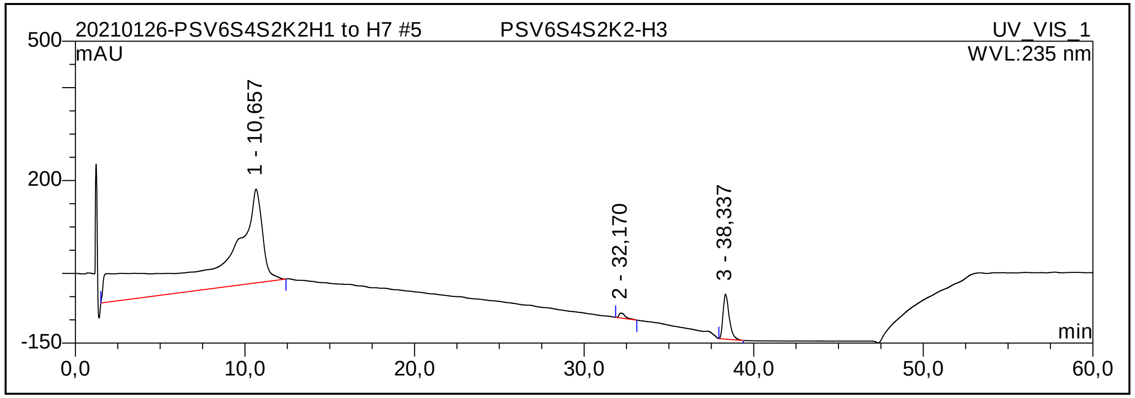

Figure **S55.** HPLC chromatogram (MeOH) of compound G

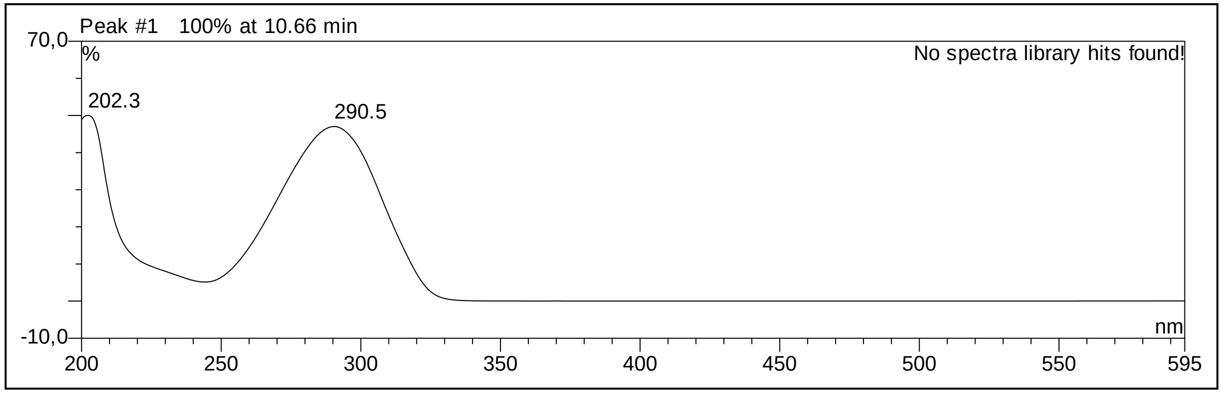

Figure **S56.** UV-Vis spectrum (MeOH) of compound G

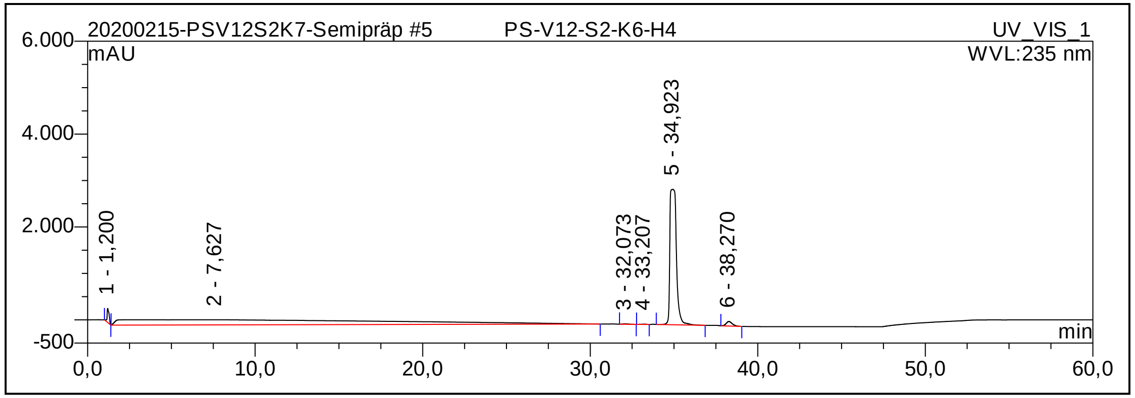

Figure **S57-** HPLC-chromatogram (MeOH) of compound H

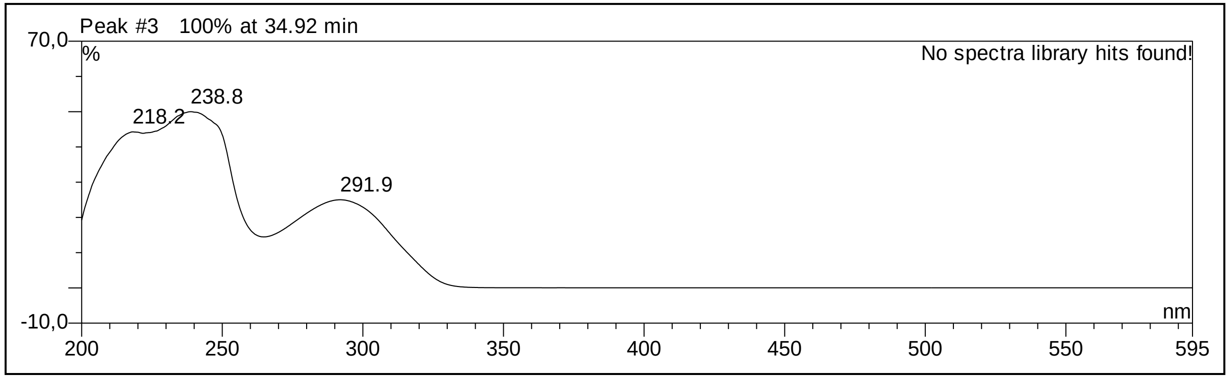

Figure **S58.** UV-Vis spectrum (MeOH) of compound H

Stereochemical studies of F

The ROESY spectrum of **F** showed correlation between the 5-H and 6-H protons of the adjacent stereogenic centers indicating their *cis* relative configuration. Therefore the conformational analysis and chiroptical calculations were performed on the arbitrarily selected *cis*-(5*S*,6*S*) stereoisomer. The Merck molecular force field (MMFF) conformational search produced 16 conformers in a 21 kJ/mol energy window which were re-optimized at the ωB97X/TZVP PCM/MeCN and the B3LYP/TZVP PCM/$CHCl_3$ levels, separately, yielding 10 and 8 low-energy conformers over 1% Boltzmann population, respectively (Figures S60 and S61). In the low-energy computed conformers, the 5,6-dihydro-α-pyrone moiety adopted a conformation, which oriented the C-5 hydroxyl group to axial and the C-6 prop-1-en-1-yl substituent to equatorial position. ECD spectra computed at four levels for the ωB97X conformers gave acceptable agreement with the experimental ECD spectrum (Figure S59) with CAM-B3LYP/TZVP giving the best agreement. Optical rotation calculations performed for the same conformers at four levels and PCM solvent model for EtOH reproduced the large experimental positive value {$[\alpha]^{24}_{D}$ *+172}* in the range of +81 – +99 (Table S1). VCD spectra computed at the B3LYP/TZVP PCM/$CHCl_3$ level for the same level optimized conformers gave good agreement with the experimental VCD spectrum (Figure X3). That is, all three applied chiroptical methods suggested (5*S*,6*S*) absolute configuration for **F** allowing elucidation of the absolute configuration as (5*S*,6*S*).

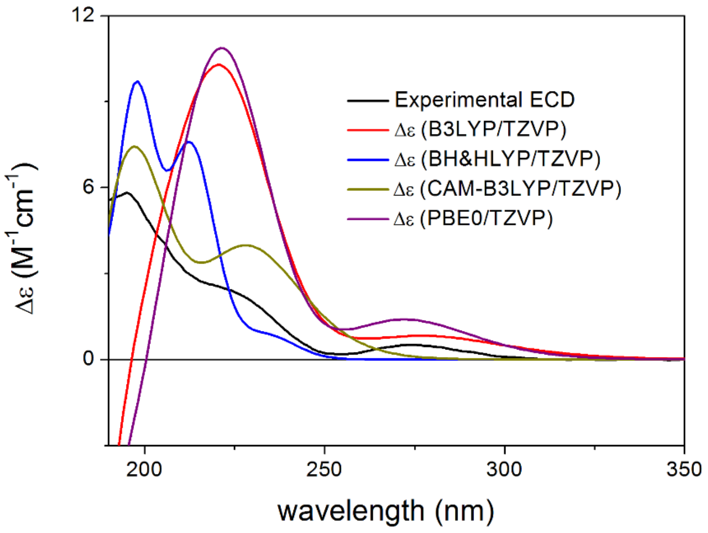

Figure S59. Comparison of the experimental ECD spectrum of **F** measured in MeCN and the calculated ECD spectra of (5*S*,6*S*)-**F** computed at various levels of theory for the 10 lowest-energy ωB97X/TZVP PCM/MeCN conformers. Black: experimental, red: B3LYP/TZVP PCM/MeCN with half-height width 4200 cm^{-1}, blue:

BH&HLYP/TZVP PCM/MeCN with half-height width 2100 cm^{-1}, olive: CAM-B3LYP/TZVP PCM/MeCN with half-height width 4200 cm^{-1}, purple: PBE0/TZVP PCM/MeCN with half-height width 4200 cm^{-1}.

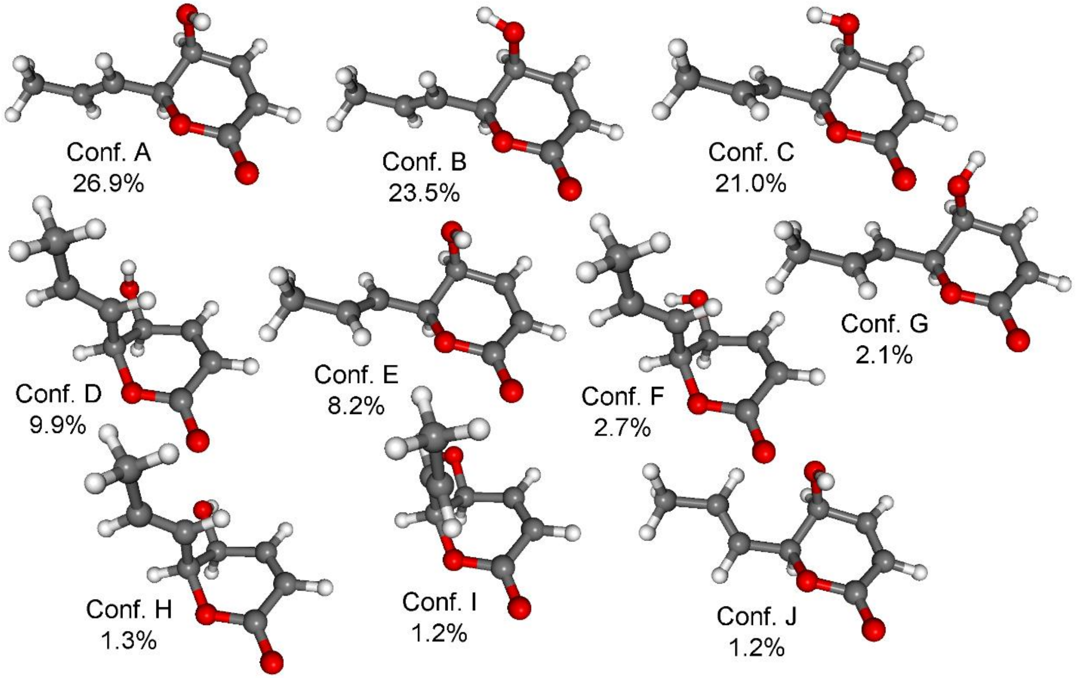

Figure S60. Geometries of the low-energy ωB97X/TZVP PCM/MeCN conformers of (5*S*,6*S*)-**F**.

Table S1. Boltzmann populations and specific optical rotations of the low-energy conformers of (5*S*,6*S*)-**F** computed at various levels for the low-energy ωB97X conformers.

Conformer	Boltzmann population	B3LYP/TZVP PCM/EtOH	BH&HLYP/TZVP PCM/EtOH	CAM-B3LYP/TZVP PCM/EtOH	PBE0/TZVP PCM/EtOH
Conf. A	26.93%	134.70	98.20	104.21	130.74
Conf. B	23.55%	29.58	5.44	9.46	29.68
Conf. C	20.95%	214.32	199.47	197.39	211.76

Conf. D	9.90%	-104.45	-80.52	-97.75	-93.21
Conf. E	8.24%	211.30	192.34	193.43	209.37
Conf. F	2.73%	-128.11	-87.40	-111.23	-111.39
Conf. G	2.09%	228.43	189.15	199.16	223.73
Conf. H	1.32%	-56.67	-26.86	-45.34	-45.81
Conf. I	1.24%	-155.48	-96.97	-122.07	-146.94
Conf. J	1.19%	263.77	211.06	223.80	260.09
Average	N/A	98.79	81.43	81.10	98.73

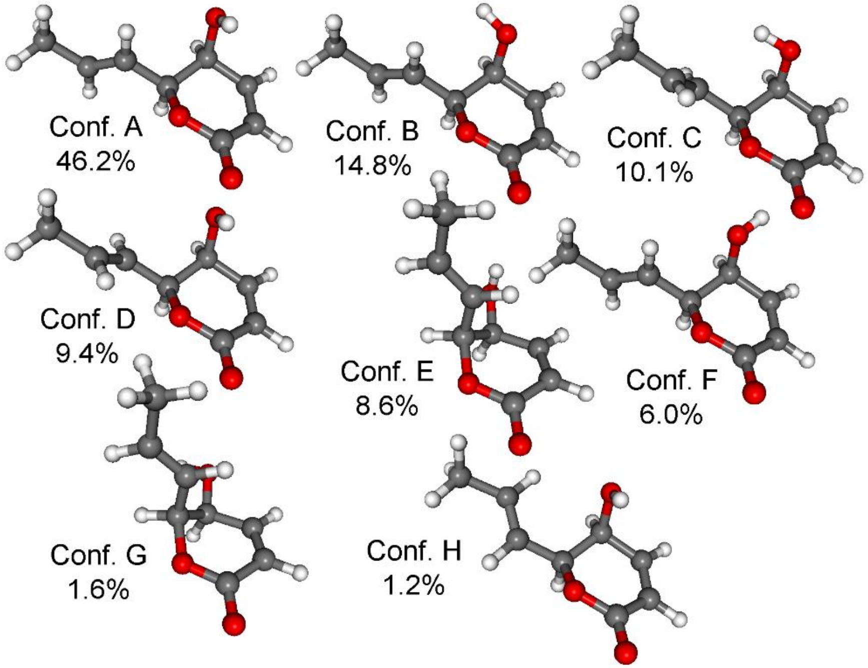

Figure S61. Geometries of the low-energy B3LYP/TZVP PCM/$CHCl_3$ conformers of (5*S*,6*S*)-**F**.

Computational details

Mixed torsional/low-frequency mode conformational searches were carried out by means of the Macromodel 10.8.011 software by using the MMFF with an implicit solvent model for $CHCl_3$ [1]. Geometry re-optimizations were carried out at the ωB97X/TZVP level with the PCM solvent model for MeCN and the B3LYP/TZVP level with PCM solvent model for $CHCl_3$. TDDFT-ECD and OR calculations were run with various functionals (B3LYP, BH&HLYP, CAMB3LYP, and PBE0) and the TZVP basis set as implemented in the Gaussian 09 package with the PCM/MeCN and the PCM/EtOH solvent models, respectively [2]. ECD spectra were generated as sums of Gaussians with 2100-4200 cm^{-1} width at half-height, using dipole-velocity-computed rotational strength values [3]. VCD spectra were generated with 8 cm^{-1} half-height width and scaled by a factor of 0.99. Boltzmann distributions were estimated from the ωB97X and the B3LYP energies. The Molekel software package was used for visualization of the results [4].

References

[1] MacroModel; Schrődinger LLC, 2015, http://www.schrodinger.com/MacroModel.

[2] Frisch, M. J.; Trucks, G. W.; Schlegel, H. B.; Scuseria, G. E.; Robb, M. A.; Cheeseman, J. R.; Scalmani, V.; Barone, G.; Mennucci, B.; Petersson, G. A.; Nakatsuji, H.; Caricato, M.; Li, X.; Hratchian, H. P.; Izmaylov, A. F.; Bloino, J.; Zheng, G.; Sonnenberg, J. L.; Hada, M.; Ehara, M.; Toyota, K.; Fukuda, R.; Hasegawa, J.; Ishida, M.; Nakajima, T.; Honda, Y.; Kitao, O.; Nakai, H.; Vreven, T.; Montgomery, J. A., Jr.; Peralta, J. E.; Ogliaro, F.; Bearpark, M.; Heyd, J. J.; Brothers, E.; Kudin, K. N.; Staroverov, V. N.; Kobayashi, R.; Normand, J.; Raghavachari, K.; Rendell, A.; Burant, J. C.; Iyengar, S. S.; Tomasi, J.; Cossi, M.; Rega, N.; Millam, J. M.; Klene, M.; Knox, J. E.; Cross, J. B.; Bakken, V.; Adamo, C.; Jaramillo, J.; Gomperts, R.; Stratmann, R. E.; Yazyev, O.; Austin, A. J.; Cammi, R.; Pomelli, C.; Ochterski, J. W.; Martin, R. L.; Morokuma, K.; Zakrzewski, V. G.; Voth, G. A.; Salvador, P.; Dannenberg, J. J.; Dapprich, S.; Daniels, A. D.; Farkas, Ö.; Foresman, J. B.; Ortiz, J. V.; Cioslowski, J.; Fox, D. J. Gaussian 09 (Revision E.01); Gaussian, Inc.: Wallingford, CT, 2013.

[3] Stephens, P. J.; Harada, N. Chirality 2009, 22, 229–233.

[4] Varetto, U. MOLEKEL 5.4; Swiss National Supercomputing Centre: Manno, Switzerland, 2009.
